# Disparities in burden of herpes simplex virus type 2 in China: systematic review, meta-analyses, and meta-regressions

**DOI:** 10.3389/fimmu.2024.1369086

**Published:** 2024-07-22

**Authors:** Yehua Wang, Xumeng Yan, Wei Ai, Yuanxi Jia, Chengxin Fan, Siyue Hu, Yifan Dai, Huachen Xue, Feifei Li, Weiming Tang

**Affiliations:** ^1^ Dermatology Hospital of Southern Medical University, Guangzhou, China; ^2^ Department of Pharmaceutical Outcomes and Policy, College of Pharmacy, University of Florida, Gainesville, FL, United States; ^3^ University of North Carolina at Chapel Hill Project-China, Guangzhou, China; ^4^ Shenzhen Institute of Advanced Technology, Chinese Academy of Science, Shenzhen, China; ^5^ School of Public Health, Nanjing Medical University, Nanjing, China

**Keywords:** herpes simplex virus type 2 (HSV-2), epidemiology, systematic review and meta-analysis, public health, prevalence

## Abstract

**Background:**

The rising prevalence of herpes simplex type 2 (HSV-2) infection poses a growing global public health challenge. A comprehensive understanding of its epidemiology and burden disparities in China is crucial for informing targeted and effective intervention strategies in the future.

**Methods:**

We followed Cochrane and PRISMA guidelines for a systematic review and included publications published in Chinese and English bibliographic systems until March 31^st^, 2024. We synthesized HSV-2 seroprevalence data across different population types. We used random-effects models for meta-analyses and conducted meta-regression to assess the association between population characteristics and seroprevalence.

**Results:**

Overall, 23,999 articles were identified, and 402 publications (1,203,362 participants) that reported the overall seroprevalence rates (858 stratified measures) were included. Pooled HSV-2 seroprevalence among the general population (lower risk) was 7.7% (95% CI: 6.8-8.7%). Compared to the general population, there is a higher risk of HSV-2 prevalence among intermediate-risk populations (14.8%, 95% CI: 11.0-19.1%), and key populations (31.7%, 95% CI: 27.4-36.1%). Female sexual workers (FSWs) have the highest HSV-2 risk (ARR:1.69, 95% CI: 1.61-1.78). We found northeastern regions had a higher HSV-2 seroprevalence than other regions (17.0%, 95% CI: 4.3-35.6%, ARR: 1.38, 95% CI: 1.26-1.50, Northern China as the reference group). This highlighted the disparity by population risk levels and regions. We also found lower HSV-2 prevalence estimates in publications in Chinese bibliographic databases than those in English databases among key populations (such as MSM and HIV-discordant populations).

**Conclusion:**

There is a gradient increase in HSV-2 prevalence risk stratification. We also identified region, population, and age disparities and heterogeneities by publication language in the HSV-2 burden. This study provides guidance for future HSV-2 prevention to eliminate disparities of HSV-2 infection and reduce overall HSV-2 burden.

**Systematic review registration:**

https://www.crd.york.ac.uk/prospero/display_record.php?RecordID=408108, identifier CRD42023408108.

## Introduction

Herpes simplex virus type 2 (HSV-2) is an incurable and recurring sexually transmitted infection (1). Often asymptomatic, people with HSV-2 can transmit the virus to their sexual partners without awareness of their infection (2, 3). Prior research has highlighted the complex interplay between HSV-2 and the host’s immune system, particularly the molecular mechanisms of viral immune evasion (4, 5), which is vital for understanding the persistence, spread, and impact of HSV-2. Given its contingency and impact on life quality and well-being (6), examining the epidemiology of HSV-2 is essential for informing future prevention. Moreover, HSV-2 poses a substantial health risk to infants because of mother-to-utero transmission during pregnancy, underscoring the importance of prenatal screening tests in maternal care (7). In addition, considering its synergy with HIV, reducing HSV-2 infection is beneficial to the goal of ending STI epidemics as major public health concerns by 2030 (8).

The latest estimate of HSV-2 global seroprevalence in 2016 was 13.2% (491 million) among people aged 15-49 worldwide (9). Recent meta-reviews have reported a wide range of seroprevalence estimates from different geographical regions, with an updated estimate of 12.1% in Asia in 2020 among the general population (10). Seroprevalences of HSV-2 among key populations such as male sex workers (MSW), men who have sex with men (MSM), and female sex workers (FSW) are substantially higher across regions, ranging from 20.6% to 74.8% for FSW and from 18.3% to 54.6% for MSM and MSW (10–15). Two additional meta-analyses that focused on key populations in China reported a pooled seroprevalence of 9.4% among MSM (16), 15.8% among FSWs (17).

Despite the existing literature, there are some gaps in achieving comprehensive pictures of HSV-2 epidemiology in China. The previous meta-analysis in Asia (10) did not delineate regional disparities across different provinces within China. Second, the search to English bibliographic databases, potentially missing studies published in Chinese bibliographic databases, such as data reporting HSV-2 seroprevalence among pregnancy screening populations (18). This might be due to topic innovation (regular screening report with standardized procedures), journal priority, study complexities (19), etc. Also, these English publications have a higher representativeness of certain provinces such as Guangdong and Yunnan than other provinces and certain populations such as female sex workers. Additionally, while the identified meta-analysis offered comparisons in estimates across various populations and subgroups in Asia, there is no risk-strata comparison specifically for China, leaving alone regional comparison. Our study aims to synthesize literature published in both Chinese and English bibliographic databases to ensure a more inclusive representation of available evidence and provide assessment of disparities in HSV-2 seroprevalence in China.

## Methods

The study protocol was registered in PROSPERO (PROSPERO ID: CRD42023408108).

### Data sources and search strategy

This systematic review was conducted under the guidance of the Cochrane Collaboration Handbook (20). We reported the findings following the Preferred Reporting Items for Systematic Reviews and Meta-Analyses (PRISMA) statement (21). We included four bibliography databases as sources, including PubMed, Embase, China National Knowledge Infrastructure (CNKI), and Wanfang. Considering the limited coverage of Chinese publications in PubMed and Embase, we included two major Chinese bibliography databases, CNKI and Wanfang (22). We searched the publications till March 31^st^, 2024.

### Eligibility criteria

We included the publications that reported primary data on HSV-2 seroprevalence, which was defined by the proportion of the included population who tested HSV-2 seropositive. We excluded case reports, case series, commentaries, reviews, and publications without access to the full text. We also excluded the studies that involved less than 10 participants. Those studies reporting HSV-related outcomes (including both HSV-1 and HSV-2) were excluded if we could not extract HSV-2 outcomes. If one study only reported HSV-2 seroprevalence from infants younger than six months, we excluded it due to the parental source antibody (23).

### Literature screening and data extraction

For each publication, two of the eight reviewers (FL, AW, XY, YW, SH, YD, HX, CF) independently screened the titles, abstracts, and full texts and identified eligible publications for data extraction. In disagreements between the two reviewers, a third reviewer (WT and YW) was consulted for reconciliation.

We extracted the variables containing information on publication year, data collection time, methods, testing assay, study population type, age, sex, sample size, and relevant study outcomes. We used pre-defined population types and risk factors to conduct stratified analyses of our study outcomes. The definition of population type and risk factors was included in the **Supplementary Materials** (**Supplementary Box S3** and **S4**). Specifically, general populations (populations at low risk) are those at lower risk of exposure to HSV-2, such as antenatal clinic attendees, blood donors, pregnant women, etc. Intermediate-risk populations are those who have frequent sexual contact with key populations and have a higher risk of exposure to HSV-2 than the general population, including truck drivers, clients of female sexual workers, bar and hotel workers, promiscuous populations or slums, and miners. Key populations are those at high risk of exposure to HSV-2 because of specific sexual risk behaviors, such as female sex workers, men who have sex with men, male sex workers, transgender populations, and injectable drug users.

“Publication” refers to an article reporting any outcome measure, while a “study” refers to details of a specific outcome measure, for example, HSV-2 seroprevalence. One publication might contain multiple study outcome measures (e.g., subgroup analyses). Duplicate or overlapping studies were included only once. Literature screening was completed by Covidence (24). Publication management was completed by Endnote X9 (25). Data extraction was completed by Microsoft Office Excel 2016.

### Quality assessment

We referred to a previously published study to assess the study quality (10). WT (University of North Carolina at Chapel Hill) is the team’s leading HSV-2 assay analysis assessment expert. Study precision was categorized into high and low based on the sample size (low: <200 vs. high ≥200). The risk of bias was assessed based on the sampling method (probability-based vs. non-probability-based) and response rate (low: <80% vs. high ≥80%). We consider studies using existing medical records as non-probability-based and of unclear response rates.

### Meta-analyses

We used the random-effects model to conduct the meta-analyses (26). Pooled means of HSV-2 seroprevalence and 95% confidence intervals were provided by using the Freeman-Tukey double arcsine transformation in the meta-analysis (27). We presented the I^2^ statistic to assess the between-study heterogeneity (28). We provided estimates of HSV-2 seroprevalence rates among different populations (i.e., general population, intermediate-risk population, key population, STI clinic attendees, HIV-positive individuals and couples, and other populations) stratified by sex. For the general population, we further provided estimates stratified by different characteristics, including age groups, regions, and year of data collection categories.

### Meta-regression

We conducted univariate and multivariable random-effects meta-regression analyses to assess the association between log-transformed seroprevalence and pre-decided factors. Variables included in the multivariable regressions were population type, age group, sex, regions, year of data collection, and study quality-related variables (i.e., assay type, sample size category, sampling method, response rate category). Two multivariable analyses were carried out, one using the year of data collection (categorical) and the other using the year of publication (categorical). A *p*-value < 0.05 (two sides) in the multivariable analysis indicated a statistically meaningful association.

### Language bias assessment

Pooled means of HSV-2 seroprevalence from studies published in Chinese and English bibliographic databases were calculated separately, stratified by population types, age groups, sex, and year of data collection categories. We further used meta-regressions to assess the association between HSV-2 seroprevalence and language of the bibliography systems (Chinese versus English (Reference group)) using relative risk as the measure within different demographic and risk strata. The regression models adjusted for the potential confounding factors related to study quality, including assay type, sample size category, sampling method, and response rate category. We hypothesized there would be a difference in HSV-2 seroprevalence estimation between the studies identified from Chinese and English bibliographic databases, suggesting the existence of publication bias related to language (29).

Meta-analyses, meta-regression, and mapping were conducted in R, version 4.3.0, using the ‘meta’ and ‘metafor’ packages (30, 31).

## Results

### Search results and scope of evidence

We identified 23,999 publications from four bibliographic databases (PubMed 1,170, Embase 16,074, CNKI 2,558, and Wanfang 4,197). Based on duplicate removal and the abstract and title screening, 1,085 publications were eligible for full-text screening, and 683 were further excluded, leaving 402 publications that met the eligibility criteria.

**Figure 1:**
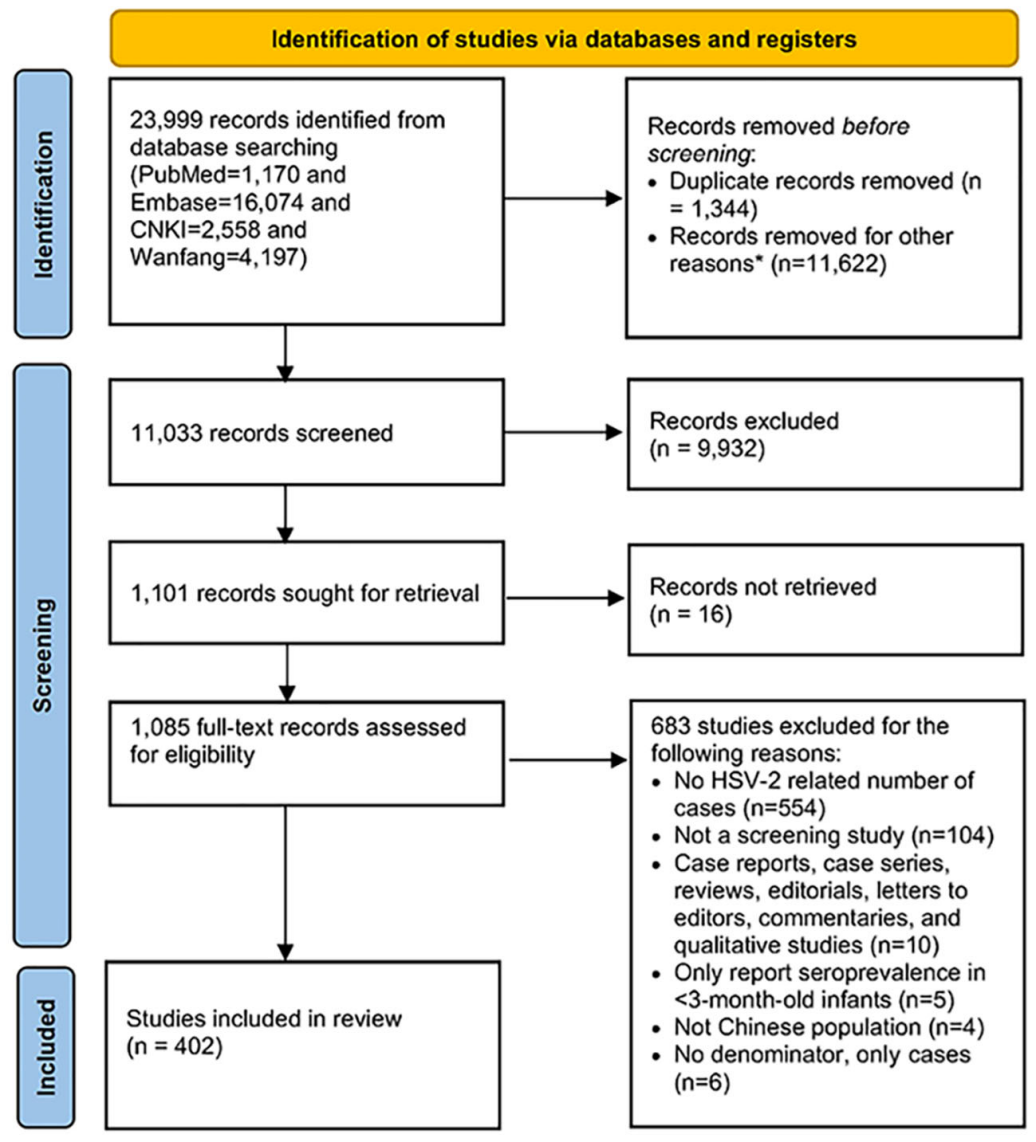
Preferred Reporting Items for Systematic Reviews and Meta-analysis (PRISMA) chart for the literature search.

The 402 unique publications (60 in English and 342 in Chinese) reported the 858 study measures of overall seroprevalence. The PRISMA flowchart of article selection is summarized in **Figure 1**. For study settings, 84.8% of the publications involved more than 200 participants, and 93.3% used non-probability-based methods. About one-fifth of publications had more than 80% response rates. Most of the studies used convenience sampling (n=349, 86.8%). Regarding data collection periods, most publications conducted their research after 2000, with 42.9% of publications from 2000-2010 and 44.8% after 2010. All seroprevalence measures are summarized in **Tables 1** and **2**.

**Table 1:** Pooled estimates for HSV-2 seroprevalence in China.

Population type	Outcome measures	Samples	HSV-2 seroprevalence (%)		Pooled HSV-2 seroprevalence	Heterogeneity measures
	Total n^a^	Total N ^b^	Range	Median	Mean (%) (95% CI)	I²^c^ (%) (95% CI)
**General populations**	488	1,066,101	0.0-85.3	6.8	7.7 (6.8-8.7)	99.6 (99.6-99.6)
Women	388	818,005	0.0-85.3	5.9	7.1 (6.1-8.2)	99.6 (99.6-99.6)
Men	50	44,917	0.0-42.1	6.8	6.8 (4.9-8.9)	96.0 (95.3-96.6)
Mixed sexes	50	203,179	0.0-59.2	11.6	13.9 (10.2-18.0)	99.3 (99.2-99.3)
**Intermediate-risk populations^d^**	29	13,131	3.9-40.5	12.0	14.8 (11.0-19.1)	96.6 (95.9-97.2)
Women	4	3,018	8.2-34.3	16.9	18.1 (8.1-30.9)	98.2 (97.0-98.9)
Men	25	10,113	3.9-40.4	12.0	14.3 (10.2-18.9)	96.4 (95.5-97.1)
Mixed sexes	–	–	–	–	–	–
**Key populations^e^**	155	62,607	0.0-88.0	28.9	31.7 (27.4-36.1)	99.2 (99.2-99.3)
Women	76	26,636	3.1-88.0	57.4	51.5 (46.1-56.9)	98.0 (97.8-98.2)
Men	68	34,767	0.0-78.7	10.8	12.4 (10.0-15.0)	96.8 (96.4-97.2)
Mixed sexes	11	1,204	4.8-62.5	47.4	39.4 (26.8-52.6)	95.7 (93.8-97.0)
**STI clinic attendees and symptomatic populations**	142	43,442	0.0-81.9	23.4	24.1 (20.9-27.4)	98.6 (98.5-98.7)
Women	33	13,982	0.0-71.8	26.7	26.6 (18.9-35.2)	99.3 (99.3-99.4)
Men	29	14,150	0.7-59.2	18.2	17.5 (12.2-23.6)	98.7 (98.6-98.9)
Mixed sexes	80	15,310	0.0-81.9	23.8	25.6 (21.7-29.7)	96.1 (95.6-96.5)
**HIV-positive individuals and individuals in HIV-discordant couples**	34	8,273	0.5-65.5	31.8	26.5 (20.3-33.1)	96.9 (96.3-97.4)
Women	–	–	–	–	–	–
Men	5	1,625	0.5-52.8	27.3	23.6 (6.8-46.3)	99.3 (99.0-99.5)
Mixed sexes	29	6,648	2.0-65.5	33.3	27.0 (20.7-33.8)	93.5 (91.7-94.9)
**Other populations^f^**	10	9,808	1.2-46.1	4.2	10.0 (3.1-20.1)	97.6 (96.7-98.2)
Women	1	89	46.1-46.1	46.1	46.1 (35.4-57.0)	—
Men	1	456	19.3-19.3	19.3	19.3 (15.8-23.1)	—
Mixed sexes	8	9,263	1.2-39.1	3.6	6.2 (1.5-13.8)	95.8 (93.6-97.3)

CI, Confidence interval; FSWs, Female sex workers; HIV, Human immunodeficiency virus; HSV-2, Herpes simplex virus type 2; MSM, Men who have sex with men; MSWs, Male sex workers; STI, Sexually transmitted infection. a Total n represents the number of studies. b Total N means the total sample size included in relevant studies. c I2: A measure that assesses the magnitude of between-study variation that is due to actual differences in HSV-2 seroprevalence across studies rather than chance. d Intermediate-risk populations include truck drivers, clients of FSW, bar and hotel workers, promiscuous populations/slums, and miners. e Key populations include FSWs, MSM/MSWs, and drug users. f Other populations include populations with an undetermined risk of acquiring HSV-2 infection such as patients with cervical cancer. No meta-analysis was done due to the small number of studies (n <3).

**Table 2:** Pooled estimates for HSV-2 seroprevalence in the general populations in China.

Population type	Outcomes	Samples	HSV-2 seroprevalence (%)		Pooled HSV-2 seroprevalence	Heterogeneity measures
Subgroup	Total n^a^	Total N^b^	Range	Median	Mean (%) (95% CI)	I²^c^ (%) (95% CI)
Region						
North	33	43,634	0.1-25.9	8.5	7.1 (5.1-9.4)	98.5 (98.3-98.7)
Northeastern	6	5,011	2.4-54.9	12.5	17.0 (4.3-35.6)	98.8 (98.4-99.2)
Eastern	184	490,239	0.0-85.3	6.9	7.1 (5.8-8.5)	99.6 (99.6-99.6)
Central-southern	126	282,995	0.0-71.0	8.0	9.5 (7.6-11.7)	99.6 (99.6-99.6)
Southwestern	54	107,924	0.1-74.6	4.0	7.4 (4.5-11.0)	99.7 (99.7-99.7)
Northwestern	48	129,566	0.0-84.2	3.3	3.3 (1.6-5.5)	99.6 (99.6-99.6)
SARs^d^ and Taiwan	30	1,363	0.0-42.1	13.1	10.4 (6.4-15.11)	84.6(79.0-88.7)
Multiple or unknown	7	5,369	7.3-68.2	18.5	23.2 (10.9-38.4)	98.4 (97.8-98.9)
Age group						
<20	37	18,052	0.0-60.0	4.9	6.0 (2.9-10.0)	99.2 (99.1-99.3)
20-29 years	72	122,872	0.0-31.1	4.6	5.3 (3.9-7.1)	99.0 (98.9-99.0)
30-39 years	46	62,911	0.0-29.3	8.4	8.0 (6.0-10.3)	99.2 (99.1-99.2)
40-49 years	26	16,992	0.0-29.0	11.5	9.5 (5.6-14.2)	99.0 (98.9-99.2)
50-59 years	12	1,232	8.5-24.5	15.9	16.7 (13.4-20.3)	49.3 (1.5-73.9)
60+ years	8	4,077	1.4-42.1	25.3	21.6 (10.3-35.3)	97.4 (96.3-98.2)
Mixed	287	839,965	0.0-85.3	6.6	7.8 (6.6-9.2)	99.7 (99.7-99.7)
Year of data collection category						
≤2000	27	22,312	0.0-84.2	10.6	14.1 (7.2-22.8)	99.5 (99.4-99.5)
2001-2010	190	321,481	0.0-71.0	5.6	6.7 (5.3-8.2)	99.5 (99.5-99.5)
>2010	238	704,959	0.0-85.3	6.7	6.9 (5.9-8.0)	99.6 (99.6-99.6)
Unknown	33	17,349	0.7-74.6	10.1	17.0 (10.9-23.9)	99.3 (99.2-99.3)

CI, Confidence interval; HSV-2, Herpes simplex virus type 2. a Total n represents the number of studies. b Total N means the total sample size included in relevant studies. c I^2^: A measure that assesses the magnitude of between-study variation that is due to actual differences in HSV-2 seroprevalence across studies rather than chance. d SARs: Special administrative regions, including Hong Kong and Macau.

### Seroprevalence disparities

#### Population disparities

Pooled mean HSV-2 seroprevalence was highest at 31.7% (n=155, 95% CI: 27.4%-36.1%) among key populations (i.e., FSM, MSM/MSW, and drug users), followed by 26.5% (n=34, 95% CI: 20.3%-33.18%) among people living with HIV (PLWH) and their couples, 24.1% (n=142, 95% CI: 20.9%-27.4%) among STI clinic attendees and symptomatic populations, 14.8% (n=29, 95% CI: 11.0%-19.1%) among intermediate-risk populations, and 7.7% (n=488, 95% CI: 6.8%-8.7%) among general population. In all populations except for HIV-positive and individuals in HIV-discordant couples, women had higher pooled seroprevalence than men (**Table 1**).

#### Regional disparities

Considering the study population size, we summarized stratified HSV-2 seroprevalence of the general populations by region, age group, and year of data collection category in **Table 2**. Pooled seroprevalence was higher in the Northeast region at 17.0% (n=6, 95% CI: 4.3-35.6%), followed by the special administrative regions (SARs, Hongkong and Macau) and Taiwan region at 10.4% (n=30, 95% CI: 6.4-15.1%).

#### Age disparities

The pooled seroprevalence increased with age from the age group 20-29 years. Pooled seroprevalence was 5.3% (95% CI:3.9-7.1%) among 20-29-year-olds (n=72), followed by 8.0% (95% CI: 6.0-10.3%) among 30-39-year-olds (n=46), 9.5% (95% CI: 5.6-14.2%) in those aged 40-49 years (n=26), 16.7% (95% CI: 13.4-20.3%) in those aged 50-59 years (n=12), and 21.6% (95% CI: 10.3-35.3%) in those aged ≥60 years (n=8).

#### Time disparities

Pooled seroprevalence decreased with the year of data collection. Pooled seroprevalence was 14.1% (95% CI:7.2-22.8%) for data collected in 2000 and earlier (n=27), followed by 6.7% (95% CI: 5.3-8.2%) during 2001-2010 (n=190) and 6.9% (95% CI: 5.9-8.0%) after 2010 (n=238).

High and significant heterogeneity was found between studies (p-value <0.001, I^2^>50%). Forest plots of the HSV-2 seroprevalence of different strata were displayed in **Supplementary Figure S1**.

### Meta-regression results

The results of univariate and multivariable meta-regression analyses further confirmed the disparities regarding the HSV-2 burden in China (**Table 3**).

**Table 3:** Univariate and multivariable meta-regression analyses for HSV-2 seroprevalence in China.

		Total n ^a^	Total N ^b^	Outcome measures RR (95%CI)	Univariable analysis LR test p-value	Adjusted R2 (%)	Multivariable analysis Model 1^c^	Multivariable analysis Model 2^d^
							ARR (95% CI)	ARR (95% CI)
Population characteristics								
**Population type**	General population	488	1,066,101	1.0	<0.001	39.80	1.00	1.00
	Intermediate risk population	29	13,131	1.1 (1.0-1.2)			**1.14 (1.06-1.25)**	**1.15 (1.06-1.25)**
	FSWs	71	25,799	1.7 (1.6-1.8)			**1.69 (1.61-1.78)**	**1.69 (1.60-1.78)**
	MSM/MSWs	67	33,837	1.1 (1.0-1.1)			**1.11 (1.04-1.19)**	**1.09 (1.02-1.16)**
	Other key populations	16	2,359	1.6 (1.4-1.8)			**1.51 (1.37-1.67)**	**1.55 (1.40-1.71)**
	STI clinics attendees	142	43,442	1.3 (1.2-1.3)			**1.18 (1.13-1.24)**	**1.17 (1.12-1.22)**
	HIV positive population and individual in HIV discordant couples	34	8,273	1.3 (1.2-1.4)			**1.20 (1.10-1.30)**	**1.18 (1.09-1.28)**
	Other populations^e^	10	9,808	1.0 (0.9-1.2)			0.98 (0.87-1.11)	0.98 (0.86-1.11)
**Age group**	<20	77	24,245	1.0	0.091	0.54	1.00	1.00
	20-29 years	114	134,103	1.0 (0.9-1.1)			1.04 (0.98-1.10)	1.04 (0.99-1.11)
	30-39 years	75	69,017	1.1 (1.0-1.2)			**1.11 (1.05-1.18)**	**1.12 (1.05-1.19)**
	40-49 years	40	19,035	1.1 (1.0-1.2)			**1.11 (1.03-1.20)**	**1.11 (1.03-1.20)**
	50-59 years	18	1,764	1.2 (1.0-1.3)			**1.23 (1.11-1.36)**	**1.21 (1.10-1.35)**
	60+ years	16	4,770	1.2 (1.0-1.4)			**1.25 (1.12-1.39)**	**1.23 (1.10-1.38)**
	Mixed	518	950,428	1.0 (1.0- 1.1)			**1.09 (1.04-1.15)**	**1.09 (1.04-1.15)**
**Sex**	Women	502	861,730	1.0	<0.001	3.34	1.00	1.00
	Men	178	106,028	1.0 (1.0-1.0)			**0.95 (0.91-0.997)**	0.97 (0.92-1.01)
	Mixed sexes	178	235,604	1.1 (1.1-1.2)			**1.07 (1.02-1.11)**	**1.06 (1.01-1.10)**
**Regions**	North	66	60,394	1.0	<0.001	10.33	1.00	1.00
	Northeastern	27	12,841	1.4 (1.3-1.6)			**1.38 (1.26-1.50)**	**1.37 (1.26-1.50)**
	Eastern	299	552,658	1.1 (1.0-1.1)			**1.05 (1.00-1.11)**	1.05 (0.99-1.10)
	Central-southern	203	312,450	1.0 (1.0-1.1)			**1.09 (1.03-1.15)**	**1.08 (1.02-1.14)**
	Southwestern	149	140,511	1.2 (1.1-1.3)			**1.10 (1.04-1.16)**	**1.09 (1.03-1.15)**
	Northwestern	57	134,556	0.9 (0.8-1.0)			0.95 (0.89-1.02)	0.96 (0.89-1.02)
	SARs^f^ and Taiwan	33	1,553	1.1 (1.0-1.2)			1.06 (0.96-1.17)	1.04 (0.94-1.15)
	Multiple or unknown	24	18,399	1.2 (1.1-1.3)			**1.18 (1.07-1.29)**	**1.17 (1.06-1.28)**
Temporal variables								
**Year of data collection**	≤2000	46	25,468	1.0	<0.001	2.17	1.00	–
	2001-2010	368	385,432	0.9 (0.9-1.0)			**0.89 (0.83-0.95)**	–
	>2010	384	765,706	0.9 (0.8-1.0)			**0.91 (0.85-0.97)**	–
	Unknown	60	26,756	1.0 (0.9-1.1)			1.00 (0.93-1.09)	–
**Year of publication**	≤**2005**	117	104,754	1.0	<0.001	3.85	–	1.00
	**2006-2015**	484	457,461	1.0 (1.0-1.1)			–	1.00 (0.95-1.04)
	**>2015**	257	641,147	0.9 (0.9-1.0)			–	1.00 (0.94-1.04)

ARR, Adjusted risk ratio; CI, Confidence interval; HIV, Human immunodeficiency virus; HSV-2, Herpes simplex virus type 2; LR, Likelihood ratio; RR, Risk ratio; STI, Sexually transmitted infection. a Total n represents the number of studies. b Total N means the total sample size included in relevant studies. c Variance explained by multivariable model 1 (adjusted R2) = 49.72%. d Variance explained by multivariable model 2 (adjusted R2) = 47.80%. Model 1 used the year of data collection as continuous and model 2 used the year of publication as categorical. e Other populations include populations with an undetermined risk of acquiring HSV-2 infection such as patients with cervical cancer. f SARs: Special administrative regions, including Hong Kong and Macau. g Variables assessing study methodology characteristics were included in multivariable analyses as covariates, including assay type, sample size category, sampling method, and response rate category.Bold values mean statistically significant results.

The first multivariable model (using the categorical data collection year as the temporal variable) explained 49.7% of the variation in seroprevalence. Compared to general populations, FSWs had the highest HSV-2 seroprevalence with an adjusted risk ratio (ARR) of 1.69 (95% CI: 1.61- 1.78), followed by other key populations (mainly drug users, ARR=1.51, 95% CI: 1.37-1.67), PLWH and HIV-negative individual in HIV discordant couples (ARR=1.20, 95% CI: 1.10-1.3), intermediate-risk populations (ARR=1.14, 95% CI: 1.06-1.25), STI clinic attendees (ARR=1.18, 95% CI: 1.13-1.24), and MSM/MSWs (ARR=1.11, 95% CI: 1.04-1.19).

Compared to those aged <20 years, HSV-2 seropositivity was highest in those aged ≥60 years (ARR=1.25, 95% CI: 1.12-1.39) and 50-59 years (ARR=1.23, 95% CI: 1.11-1.36). Compared to people living in the northern part of China (reference group), people living in the Northeast have the highest risk of HSV-2 (ARR=1.38, 95% CI: 1.26-1.50), followed by those living in the Southwest (ARR=1.10, 95% CI: 1.04-1.16) and Central-southern regions (ARR=1.09, 95% CI: 1.03-1.15). Men have a similar but slightly lower HSV-2 seroprevalence compared to women (ARR=0.95, 95% CI: 0.91-0.997). Compared to data collected before 2000, data collected during 2001-2010 (ARR=0.89, 95% CI: 0.83-0.95) and after 2010 (ARR=0.91, 95% CI: 0.85-0.97) had lower HSV-2 seroprevalence. Having identified the Northeastern region with the highest ARR, we conducted additional multivariable meta-regressions within each available risk stratum, altering the region variable into Northeastern region vs. non-Northeastern regions. Results revealed that individuals in the Northeastern region have significantly higher HSV-2 seroprevalence than those in non-Northeastern regions within STI clinic attendees (ARR=1.31 95% CI: 1.17-1.47) and key populations (ARR=1.51, 95% CI: 1.28-1.78).

The second model (using the categorical publication year as the temporal variable) explained 47.8% of the variation in HSV-2 seroprevalence, yielding results similar to those of the first model. The seroprevalence did not vary by year of publication.

### Language bias between publications identified in Chinese and English bibliographic databases

We summarized the differences in pooled HSV-2 seroprevalence between publications identified in Chinese and English bibliographic databases in **Table 4**. More publications were identified in Chinese bibliographic databases (342 identified in CNKI and Wanfang *vs*. 60 identified in PubMed and Embase).

**Table 4:** Comparing pooled estimates and adjusted odds ratio of HSV-2 seroprevalence in Chinese and English databases in China.

		Pooled HSV-2 seroprevalence Mean (%) (95% CI)		Multivariable analysis model^a^
		English	Chinese	Chinese vs. English (Ref) ARR (95% CI)
Population characteristics				
**Population type**	General population	9.0 (7.1, 11.0)	7.5 (6.4, 8.5)	0.99 (0.93, 1.05)
	Intermediate risk population	9.6 (6.3, 13.5)	19.7 (13.9, 26.3)	**1.17 (1.06, 1.29)**
	FSWs	60.4 (55.0, 65.7)	41.5 (33.3, 49.9)	0.88 (0.73, 1.06)
	MSM/MSWs	16.8 (12.6, 21.5)	9.1 (6.7, 11.7)	**0.87 (0.80, 0.93)**
	Other key populations	30.6 (17.4, 45.6)	63.1 (56.7, 69.2)	**1.41 (1.10, 1.79)**
	STI clinics attendees	21.2 (8.2, 38.1)	24.2 (21.0, 27.6)	1.02 (0.85, 1.23)
	HIV positive population and individual in HIV discordant couples	32.1 (28.2, 36.0)	24.4 (16.4, 33.4)	0.87 (0.71, 1.08)
	Other populations^b^	46.1 (35.8, 56.5)	7.4 (2.3, 14.9)	0.77 (0.48, 1.23)
**Age group**	<20	8.7 (2.0, 18.9)	12.0 (8.0, 16.6)	0.88 (0.73, 1.05)
	20-29 years	15.9 (8.9, 24.4)	10.9 (7.8, 14.4)	**0.84 (0.75, 0.95)**
	30-39 years	16.8 (9.0, 26.3)	16.7 (11.5, 22.5)	0.93 (0.79, 1.10)
	40-49 years	10.7 (4.9, 18.3)	19.6 (12.9, 27.2)	0.97 (0.78, 1.21)
	50-59 years	15.4 (11.9, 19.1)	27.1 (18.8, 36.2)	0.91 (0.80, 1.04)
	60+ years	32.0 (24.6, 39.9)	17.4 (7.6, 29.8)	**0.65 (0.43, 0.97)**
	Mixed	27.3 (22.7, 32.2)	12.4 (10.9, 14.0)	**0.82 (0.78, 0.87)**
**Sex**	Women	26.7 (21.4, 32.4)	10.4 (8.9, 12.0)	**0.74 (0.70, 0.80)**
	Men	11.0 (8.7, 13.5)	13.1 (10.8, 15.6)	1.00 (0.94, 1.07)
	Mixed sexes	33.5 (28.0, 39.3)	20.6 (17.8, 23.6)	**0.83 (0.74, 0.93)**
Temporal variables				
**Year of data collection**	<=2000	32.8 (18.6, 48.8)	13.8 (5.9, 24.1)	**0.72 (0.55, 0.95)**
	2001-2010	20.7 (16.7, 24.9)	13.2 (11.1, 15.4)	**0.78 (0.73, 0.84)**
	>2010	17.6 (13.1, 22.6)	11.3 (9.8, 12.8)	**0.91 (0.85, 0.97)**
	Unknown	11.4 (3.1, 23.9)	22.8 (16.9, 29.2)	**1.35 (1.07, 1.71)**

RR, Risk ratio; CI, Confidence interval; HIV, Human immunodeficiency virus; HSV-2, Herpes simplex virus type 2; STI, Sexually transmitted infection; FSW, female sexual worker; MSW, male sexual worker; MSM, men who have sex with men. a Variables assessing study methodology characteristics were included in multivariable analyses as covariates, including assay type, sample size category, sampling method, and response rate category. b Other populations include populations with an undetermined risk of acquiring HSV-2 infection such as patients with cervical cancer.Bold values mean statistically significant results.

Within different risk population strata, we found the HSV-2 seroprevalence was significantly lower in publications from Chinese databases compared to those from English databases among MSM/MSWs (9.1% vs. 16.8%, ARR=0.87, 95% CI: 0.80-0.93). The pooled mean of HSV-2 seroprevalence among PLWH and HIV-negative individuals in HIV-discordant couples (24.4% vs. 32.1%), and FSWs (41.5% vs. 60.4%) were also lower in publications from Chinese databases, but the RRs were non-significant after accounting for assay type, sample size, sampling method, and response rate. In comparison, we observed a higher pooled seroprevalence among intermediate-risk populations (e.g., truck drivers, sexual workers’ clients, etc.) in publications identified from Chinese bibliographic databases than those from English databases (19.7% vs. 9.6%, ARR=1.15, 95% CI: 1.02-1.29) and other key populations (mainly drug users) (63.1% vs. 30.6%, ARR=1.41, 95% CI: 1.10-1.79). No significant difference in HSV-2 seroprevalence between Chinese and English databases was observed in the general population.

Within demographic variable strata, the pooled seroprevalence was lower in Chinese publications compared to English publications (reference group) among individuals aged 20-29 years (10.9% vs. 15.9%, RR=0.84, 95% CI: 0.75-0.95) and those aged 60 years or older (17.4% vs. 32.0%, RR=0.65, 95% CI: 0.43-0.97). Moreover, publications from Chinese databases also yielded significantly lower pooled seroprevalence among females (10.4% vs. 26.7%, ARR=0.74, 95% CI: 0.70-0.80) and populations with mixed sexes (20.6% vs. 33.5%, ARR=0.83, 95% CI: 0.74-0.93). Across three distinct data collection periods (i.e., before 2000, from 2001 to 2010, and after 2010), the pooled seroprevalence in publications from English databases consistently surpassed that in publications from Chinese databases (before 2000: RR=0.72, 95% CI: 0.55-0.95; 2001-2010: RR=0.78, 95% CI: 0.73-0.84; after 2010: RR=0.91, 95% CI: 0.85-0.97, respectively).

### Quality assessment

The quality assessment of 402 seroprevalence publications was summarized in **Supplementary Table S10**. 341 publications (84.8%) demonstrated high precision in assessing seroprevalence measures, with a higher proportion of high-precision publications found in English databases compared to Chinese ones (90.0% vs. 83.9%, p=0.01). 27 publications (6.7%) had low risk of bias (ROB) in the sampling method domain, and 84 publications (20.9%) had low ROB in the response rate domain. Publications from the English bibliographic database had lower ROBs than those from the Chinese database in sampling method (p<0.001) and response rate domains (p=0.001). Only 12 publications (3.0%) had low ROB in both quality domains, and nine publications (2.2%) had high ROB in both domains. The proportion of publications with low ROB in both quality domains was higher in the English bibliographic database than in the Chinese database, and the proportion of publications with high ROB in both domains was also higher than that in the Chinese database.

## Discussion

This systematic review included publications identified from major Chinese and English bibliography databases and involved over one million study participants. It adds to the existing literature by providing a more updated, detailed synthesis of HSV-2 epidemiology in China by comparing the disease burdens across different characteristic strata to assess the potential disparities. We also assessed the potential language bias existing between Chinese and English publications.

The HSV-2 seroprevalence among the general population is 7.7% (6.8-8.8%), similar to the overall seroprevalence of HSV-2 in Asia (10), lower than in Europe, Sub-Saharan Africa, and Australia but higher than the Middle East and North Africa (11, 12, 14, 15). Chronologically, through pooled estimates and meta-regression, we observed that the HSV-2 seroprevalence in China decreased after 2000, related to improved STI education among the general population nationwide (32, 33). The seroprevalence stayed constant after 2010. It can partially be explained by the expansion of pregnancy health examinations promoted by the National Free Preconception Health Examination Project starting from 2010, capturing more previously undetected cases (34). It also reveals the potential unmet needs for HSV-2 prevention among key populations.

Our comprehensive analysis revealed nuanced patterns in HSV-2 seroprevalence stratified by various factors. Stratification based on the level of risk demonstrated a gradient increase in HSV-2 seroprevalence from low to key populations, using the general population as the reference group. This finding, obtained through meta-regressions, revealed sustained HSV-2 transmission within key populations (17). Despite comparable HSV-2 seroprevalence between men and women overall in multivariable meta-regressions, we observed numerically higher HSV-2 seroprevalence among women than men in multiple risk subgroups (**Table 1**). Notably, among key populations, we found FSW were more vulnerable to HSV-2 than MSM/MSW (ARR 1.69 (1.61-1.78) vs. 1.11 (1.04-1.19)). This not only emphasized the necessity to continue healthcare and behavioral intervention to reduce HSV-2 disease burdens among vulnerable populations but also indicated sex disparities regarding HSV-2 vulnerabilities. Stratifying by age group showed a steady increase in HSV-2 seroprevalence with age, consistent with increasing cumulative exposure risk to the virus over the sexual life span and incurability of HSV-2: once infected, the antibody test will always be positive.

Geographically, we found that the Northeastern region (Liaoning, Jilin, and Heilongjiang) had the highest pooled HSV-2 seroprevalence. This pattern is likely to be driven by the high proportion of STI clinic attendees with suspected genital herpes symptoms and MSM living with HIV within this sample (7,766/12,841, 60.5%). To account for this factor, we did risk-level stratification and adjusted for demographic and study quality factors using multivariable meta-regression. Within each risk stratum, we still observed a consistently higher HSV-2 seroprevalence in the Northeastern region compared to other non-Northeastern regions. This may suggest a higher HSV-2 burden in the Northeastern region, revealing a potential unmet health need.

Stratified by the publication languages, we found several heterogeneities between the publications identified in Chinese and English bibliographic databases. First, the composition of the study population pronouncedly differed between the Chinese and English databases. The sample size of general populations in publications from Chinese databases is more than 15-fold that from English bibliography databases, mainly attributable to the large-scale toxoplasmosis, rubella cytomegalovirus, herpes simplex, and HIV (TORCH) screening among pre-pregnancy and pregnancy women in clinical and community settings. Second, studies identified from Chinese bibliography databases were more geographically diverse, providing HSV-2 seroprevalence not just in southern China (e.g., Guangdong and Yunnan) but around the country. The observed heterogentities might be due to several reasons: (1) The regular TORCH screening results among the general population were harder to publish in journals indexed in English bibliographic databases than in Chinese databases (35). (2) Key populations had higher HSV-2 vulnerabilities and potentially higher public health significance (36). Investigators promoted their results in journals indexed in English databases for wider attention and higher citations (37). We also compared the quality of studies and found publications from English databases have a lower risk of bias than those from Chinese databases (**Supplementary Table S10**). Multivariable meta-regressions yielded consistently higher estimates of HSV-2 seroprevalence in publications from English databases except within the intermediate risk group. This suggests potential language bias that overestimated the HSV-2 seroprevalence by only including publications published in English bibliography databases. While the Cochrane Handbook for Systematic Reviews and the United States Institute of Medicine Guidelines for Systematic Reviews recommend including non-English-language literature published in English bibliographic databases in the review (20, 38), our study revealed that excluding non-English databases for literature search can also affect the disease burden estimation. Such language bias may distort synthesized results, leading to misinterpretation of the disease burden and suboptimal distribution of public health resources in HSV-2 prevention across different populations.

There are some limitations to be noted. First, we did not include all Chinese bibliographic databases in the literature search scope. There are other databases, such as Weipu Database. However, we have included the two most popular Chinese bibliographic databases in this review and have yet to observe differences regarding the impact of journals published in different Chinese databases. Thus, the potential publication bias of excluding other Chinese databases is insignificant. Secon, due to heterogeneities in variable categorization (such as age group), some studies’ subgroups cannot be extracted and are categorized into mixed groups or other populations. This will lead to loss of information. We try to set each age interval to be 10 years to enable more study results to fit into these categories and to reduce the number of studies grouped into “mixed-age”. Third, the included studies had an overall low quality, with 29.4% of them having high ROB in both quality domains, and only 4.0% of the studies having low ROB in both domains. Future studies should employ probability-based sampling methods to improve study quality.

## Conclusion

There is a gradient increase in HSV-2 prevalence risk stratification. We also identified region, population, and age disparities and heterogeneities by publication language in the HSV-2 burden. This study provides health policy implications for future HSV-2 prevention to eliminate disparities of HSV-2 infection and reduce overall HSV-2 burden.

## Data availability statement

The original contributions presented in the study are included in the article/**Supplementary Material**. Further inquiries can be directed to the corresponding author.

## Author contributions

YW: Conceptualization, Data curation, Formal analysis, Investigation, Methodology, Project administration, Software, Supervision, Validation, Writing – original draft, Writing – review & editing. XY: Conceptualization, Data curation, Formal analysis, Funding acquisition, Investigation, Methodology, Software, Supervision, Visualization, Writing – original draft, Writing – review & editing. WA: Conceptualization, Data curation, Investigation, Methodology, Software, Supervision, Writing – original draft, Writing – review & editing. YJ: Conceptualization, Methodology, Writing – review & editing. CF: Data curation, Formal analysis, Writing – review & editing. SH: Data curation, Formal analysis, Writing – review & editing. YD: Data curation, Formal analysis, Writing – review & editing. HX: Data curation, Formal analysis, Writing – review & editing. FL: Data curation, Formal analysis, Writing – review & editing. WT: Conceptualization, Data curation, Formal analysis, Investigation, Methodology, Project administration, Resources, Supervision, Validation, Writing – original draft, Writing – review & editing.
