# Supplementary material for: Disparities in burden of herpes simplex virus type 2 in China: systematic review, meta-analyses, and meta-regressions
Source: Front Immunol. 2024 Jul 22;15:1369086. doi: 10.3389/fimmu.2024.1369086 (PMC11298463; doi:10.3389/fimmu.2024.1369086)
Supplement: Supplementary file 1 [file DataSheet_1.docx]

**Supplementary Material**

**Epidemiology of herpes simplex virus type 2 in China: A systematic review, meta-analysis, and meta-regression**

Table of Contents

[**Supplementary Table S1.** Preferred Reporting Items for Systematic Reviews and Meta-analyses (PRISMA) checklist 1.^1^ 3](#_Toc163948749)

[**Supplementary Table S2.** Data sources and search criteria for systematically reviewing HSV-2 epidemiology in China. 5](#_Toc163948750)

[**Supplementary Box S1.** The provinces divided by regions in China. 6](#_Toc163948751)

[**Supplementary Box S2.** Variables extracted from relevant reports meeting the inclusion criteria. 7](#_Toc163948752)

[**Supplementary Box S3.** Definitions of population type classifications. 8](#_Toc163948753)

[**Supplementary Box S4.** Factors (variables) selected *a priori* and included in univariable and multivariable meta-regression analyses. 9](#_Toc163948754)

[**Supplementary Table S3.** Studies reporting HSV-2 seroprevalence among general populations in China. 10](#_Toc163948755)

[**Supplementary Table S4.** Studies reporting HSV-2 seroprevalence among intermediate-risk populations in China. 24](#_Toc163948756)

[**Supplementary Table S5.** Studies reporting HSV-2 seroprevalence among key populations in China. 26](#_Toc163948757)

[**Supplementary Table S6.** Studies reporting HSV-2 seroprevalence among HIV positive population and individual in HIV discordant couples in China. 31](#_Toc163948758)

[**Supplementary Table S7.** Studies reporting HSV-2 seroprevalence among STI clinic attendees and symptomatic populations in China. 33](#_Toc163948759)

[**Supplementary Table S8.** Studies reporting HSV-2 seroprevalence among other populations in China. 37](#_Toc163948760)

[**Supplementary Table S9.** Distribution of publications on HSV-2 seroprevalence across different groups in China in English and Chinese databases 37](#_Toc163948761)

[**Supplementary Figure S1.** Forest plots presenting outcomes of the pooled mean HSV-2 seroprevalence among different populations in China. 39](#_Toc163948762)

[A) General populations 39](#_Toc163948763)

[B) Intermediate-risk populations 41](#_Toc163948764)

[C) Key populations 42](#_Toc163948765)

[D) STI clinic attendees and symptomatic populations 45](#_Toc163948766)

[E) HIV-positive individuals and individuals in HIV-discordant couples 48](#_Toc163948767)

[F) Other populations 49](#_Toc163948768)

[**Supplementary Table S10.** Summary of precision assessment and risk of bias (ROB) assessment for studies reporting HSV-2 seroprevalence in China. 50](#_Toc163948769)

# **Supplementary Table S1.** Preferred Reporting Items for Systematic Reviews and Meta-analyses (PRISMA) checklist 1.^1^

| **Section/topic** | **#** | **Checklist item** | **Reported in main text on** |
| --- | --- | --- | --- |
| **Title** | | |  |
| Title | 1 | Identify the report as a systematic review, meta-analysis, or both. | p. 1 |
| **Abstract** | | |  |
| Structured summary | 2 | Provide a structured summary including, as applicable: background; objectives; data sources; study eligibility criteria, participants, and interventions; study appraisal and synthesis methods; results; limitations; conclusions and implications of key findings; systematic review registration number. | p. 2-3 |
| **Introduction** | | |  |
| Rationale | 3 | Describe the rationale for the review in the context of what is already known. | p. 6-7 |
| Objectives | 4 | Provide an explicit statement of questions being addressed with reference to participants, interventions, comparisons, outcomes, and study design (PICOS). | p. 6-7 |
| **Methods** | | |  |
| Protocol and registration | 5 | Indicate if a review protocol exists, if and where it can be accessed (e.g., Web address), and, if available, provide registration information including registration number. | NA |
| Eligibility criteria | 6 | Specify study characteristics (e.g., PICOS, length of follow-up) and report characteristics (e.g., years considered, language, publication status) used as criteria for eligibility, giving rationale. | p. 7-8 |
| Information sources | 7 | Describe all information sources (e.g., databases with dates of coverage, contact with study authors to identify additional studies) in the search and date last searched. | p. 7 |
| Search | 8 | Present full electronic search strategy for at least one database, including any limits used, such that it could be repeated. | Supplementary Table 2 |
| Study selection | 9 | State the process for selecting studies (i.e., screening, eligibility, included in systematic review, and, if applicable, included in the meta-analysis). | p. 7-8 |
| Data collection process | 10 | Describe method of data extraction from reports (e.g., piloted forms, independently, in duplicate) and any processes for obtaining and confirming data from investigators. | p. 8; Supplementary Box 2 |
| Data items | 11 | List and define all variables for which data were sought (e.g., PICOS, funding sources) and any assumptions and simplifications made. | p. 8; Supplementary Box 2 |
| Risk of bias in individual studies | 12 | Describe methods used for assessing risk of bias of individual studies (including specification of whether this was done at the study or outcome level), and how this information is to be used in any data synthesis. | p. 8-9 |
| Summary measures | 13 | State the principal summary measures (e.g., risk ratio, difference in means). | p. 9 |
| Synthesis of results | 14 | Describe the methods of handling data and combining results of studies, if done, including measures of consistency (e.g., I^2^) for each meta-analysis. | p. 9 |
| Risk of bias across studies | 15 | Specify any assessment of risk of bias that may affect the cumulative evidence (e.g., publication bias, selective reporting within studies). | p. 8-9 |
| Additional analyses | 16 | Describe methods of additional analyses (e.g., sensitivity or subgroup analyses, meta-regression), if done, indicating which were pre-specified. | p. 9 |
| **Results** | | |  |
| Study selection | 17 | Give numbers of studies screened, assessed for eligibility, and included in the review, with reasons for exclusions at each stage, ideally with a flow diagram. | p. 10; Figure 1 |
| Study characteristics | 18 | For each study, present characteristics for which data were extracted (e.g., study size, PICOS, follow-up period) and provide the citations. | p. 10-12; Supplementary Tables 3-7,9 |
| Risk of bias within studies | 19 | Present data on risk of bias of each study and, if available, any outcome level assessment (see item 12). | p. 13; Supplementary Table 10 |
| Results of individual studies | 20 | For all outcomes considered (benefits or harms), present, for each study: (a) simple summary data for each intervention group (b) effect estimates and confidence intervals, ideally with a forest plot. | p. 10-13; Tables 1,2 and 4; Supplementary Figures 1 and 2 |
| Synthesis of results | 21 | Present results of each meta-analysis done, including confidence intervals and measures of consistency. | p. 10-13;  Tables 1, 2 and 4 |
| Risk of bias across studies | 22 | Present results of any assessment of risk of bias across studies (see Item 15). | p. 13; Supplementary Table 10 |
| Additional analysis | 23 | Give results of additional analyses, if done (e.g., sensitivity or subgroup analyses, meta-regression [see Item 16]). | p. 11-12; Table 3; Supplementary Table 8 |
| **Discussion** | | |  |
| Summary of evidence | 24 | Summarize the main findings including the strength of evidence for each main outcome; consider their relevance to key groups (e.g., healthcare providers, users, and policy makers). | p. 13-14 |
| Limitations | 25 | Discuss limitations at study and outcome level (e.g., risk of bias), and at review-level (e.g., incomplete retrieval of identified research, reporting bias). | p. 15 |
| Conclusions | 26 | Provide a general interpretation of the results in the context of other evidence, and implications for future research. | p. 15 |
| **funding** | | |  |
| Funding | 27 | Describe sources of funding for the systematic review and other support (e.g., supply of data); role of funders for the systematic review. | p. 16 |

Abbreviations: NA = Not applicable, p = page.

# **Supplementary Table S2.** Data sources and search criteria for systematically reviewing HSV-2 epidemiology in China.

| **PubMed (last searched March 28, 2024):** |
| --- |
| (Simplexvirus[MeSH] OR Herpes Simplex[MeSH] OR Herpes Genitalis[MeSH] OR Herpes Hominis[Text] OR HSV type-2[Text] OR HSV type 2[Text] OR HSV2[Text] OR HSV-2[Text] OR HSV [Text] OR Human herpes virus[Text] OR Herpes simplex virus type 2[Text] OR Herpes simplex virus type-2[Text] OR herpes simplex virus 2[Text] OR herpes simplex virus-2[Text] OR herpes simplex type 2[Text] OR herpes simplex type-2[Text] OR herpes simplex 2[Text] OR herpes simplex-2[Text] OR Herpesvirus type 2[Text] OR Herpesvirus type-2[Text] OR Herpesvirus 2[Text] OR Herpesvirus-2[Text] OR Herpes virus type 2[Text] OR Herpes virus type-[Text] OR Herpes virus [Text] OR Herpes virus-2[Text] OR genital herpes[Text] OR Herpes Genitalis[Text] OR Stomatitis Herpetic[Text] OR Herpes Labialis[Text]) AND (China[Text] OR Chinese[Text] OR Hong Kong[Text] OR Maca*[Text] OR Tibet*[Text] OR Taiwan*[Text]) |
| **Embase (last searched March 28 2024):** |
| (('herpes simplex' OR 'herpes simplex virus' OR 'hsv type-2' OR 'hsv type 2' OR 'hsv2' OR 'hsv-2' OR 'hsv 2' OR 'human herpes virus' OR 'herpes simplex virus type 2' OR 'herpes simplex virus type-2' OR 'herpes simplex virus 2' OR 'herpes simplex virus-2' OR 'herpes simplex type 2' OR 'herpes simplex type-2' OR 'herpes simplex 2' OR 'herpes simplex-2' OR 'herpesvirus type 2' OR 'herpesvirus type-2' OR 'herpesvirus 2' OR 'herpesvirus-2' OR 'herpes virus type 2' OR 'herpes virus type-2' OR 'herpes virus 2' OR 'herpes virus-2' OR 'genital herpes' OR 'herpes genitalis' OR 'herpes labialis' OR 'herpetic stomatitis') OR ('herpes simplex'/exp OR 'herpesviridae'/exp)) AND ('china' OR 'chinese' OR 'hong kong' OR 'maca*' OR 'tibet*' OR 'taiwan*') AND ([article]/lim OR [article in press]/lim OR [conference abstract]/lim OR [conference paper]/lim OR [conference review]/lim OR [data papers]/lim OR [review]/lim OR [preprint]/lim) AND [embase]/lim |
| **CNKI (last searched March 28, 2024):** |
| TKA=(单纯疱疹病毒2型+单纯疱疹病毒Ⅱ型+单纯疱疹病毒-2+单纯疱疹病毒-Ⅱ型+2型单纯疱疹病毒+Ⅱ型单纯疱疹病毒+单纯疱疹Ⅱ型病毒+HSV2+HSV-Ⅱ+HSV-2+殖器疱疹+生殖道单纯疱疹病毒) AND TKA=(流行病学+流行情况+患病+发病率+血清学+感染) |
| **Wanfang (last searched March 28, 2024):** |
| 主题:(单纯疱疹病毒2型 or 单纯疱疹病毒Ⅱ型 or 单纯疱疹病毒-2 or 单纯疱疹病毒-Ⅱ型 or 2型单纯疱疹病毒 or Ⅱ型单纯疱疹病毒 or 单纯疱疹Ⅱ型病毒 or HSV2 or HSV-Ⅱ or HSV-2 or 生殖器疱疹 or 生殖道单纯疱疹病毒) and 主题:(流行病学 or 流行情况 or 患病 or 发病率 or 血清学 or 感染) |

Abbreviations: HSV-2 = Herpes simplex virus type

# **Supplementary Box S1.** The provinces divided by regions in China.

- **North:** Beijing, Tianjin, Hebei, Shanxi, Inner Mongolia.
- **Northeastern:** Liaoning, Jilin, Heilongjiang.
- **Eastern:** Shanghai, Jiangsu, Zhejiang, Anhui, Fujian, Jiangxi, Shandong.
- **Central-southern:** Henan, Hubei, Hunan, Guangdong, Guangxi, Hainan.
- **Southwestern:** Chongqing, Sichuan, Guizhou, Yunnan, Tibet.
- **Northwestern:** Shaanxi, Gansu, Qinghai, Ningxia, Xinjiang
- **Special administrative regions (SARs) and Taiwan:** Hong Kong, Macau, Taiwan

# **Supplementary Box S2.** Variables extracted from relevant reports meeting the inclusion criteria.

1. Author(s)
2. Publication title
3. Publication year
4. Year(s) of data collection
5. Region
6. Province
7. City
8. Study site
9. Study design
10. Study sampling method
11. Study population type
12. Population characteristics (e.g., sex and age)
13. Diagnostic assay
14. Sample size
15. HSV-2 outcome measures
16. Response rate

Abbreviations: HSV-2 = Herpes simplex virus type 2

# **Supplementary Box S3.** Definitions of population type classifications.

| 1. **General populations** (populations at low risk): these include populations at lower risk of exposure to HSV-2, such as antenatal clinic attendees, blood donors, and pregnant women, among others. 2. **Intermediate-risk populations**: these include populations who presumably have frequent sexual contact with high-risk populations and have therefore a higher risk of exposure to HSV-2 than the general population. These include truck drivers, clients of female sexual workers, bar and hotel workers, promiscuous populations or slums, and miners. 3. **Key populations**: these include populations at high risk of exposure to HSV-2 because of specific sexual risk behaviors such as female sex workers, men who have sex with men, male sex workers, transgender populations, and people who inject drugs. 4. **STI clinic attendees and symptomatic populations**: these include patients attending STI clinics, or have clinical manifestations related to an STI. 5. **HIV-positive individuals and individuals in HIV-discordant couples**: these include populations who are HIV-positive or are in a spousal relationship with an HIV-positive individual. 6. **Other populations**: these include populations not satisfying the above definitions, or populations with an undetermined risk of acquiring HSV-2 infection. |
| --- |

Abbreviations: HSV-2 = Herpes simplex virus type 2, STI = Sexually transmitted infection, HIV = Human immunodeficiency virus.

# **Supplementary Box S4.** Factors (variables) selected *a priori* and included in univariable and multivariable meta-regression analyses.

| 1. Population type as defined in Box S3 2. Sex 3. Age groups classified to best fit reported data as:  - <20 years old - 20-29 years old - 30-39 years old - 40-49 years old - 50-59 years old - ≥60 years old - Mixed age bands  1. Regions as defined in Box S1 2. Assay type:  - Western Blot - ELISA - Immunofluorescent - PCR - Other  1. Sample size:  - <200 - ≥200  1. Sampling method:  - Probability-based sampling - Non-probability-based sampling  1. Response rate:  - ≥80% - <80% - Unclear  1. Year of publication category:  - ≤2005 - 2006-2015 - >2015  1. Year of publication as a linear term 2. Year of data collection category^*^  - ≤2000 - 2001-2010 - >2010  1. Year of data collection as a linear term |
| --- |

^*^ The categories were set based on the observed median time between the year of publication and year of data collection of 4 approximated to 5 to have 5-year brackets.

Abbreviations: ELISA = Enzyme-linked immunosorbent assay, PCR = Polymerase chain reaction.

# **Supplementary Table S3.** Studies reporting HSV-2 seroprevalence among general populations in China.

| **Author, year** | **Year(s) of data collection** | **Region** | **Study site** | **Original study design^a^** | **Sampling method** | **Population** | **HSV-WB serological assay** | **Sample size** | **HSV-2 seroprevalence (%)** |
| --- | --- | --- | --- | --- | --- | --- | --- | --- | --- |
| Bai, 2019 | 2015-17 | Northwestern | Outpatient clinics | CS | Conv | Women of childbearing age | IF | 8128 | 3.33 |
| Bai, 2021 | 2019-20 | Eastern | Outpatient clinics | Cohort | Conv | Subfertile men | ELISA | 195 | 1.03 |
| Cao, 2005 | 2003-04 | Eastern | Outpatient clinics | CS | RS | Pregnant women | ELISA | 3262 | 1.26 |
| Chang, 2013 | 2012 | Eastern | Community | CS | RS | Married woman of childbearing age | ELISA | 4765 | 0.52 |
| Chen, 2006b | 2003-06 | Eastern | Outpatient clinics | CS | Conv | Pregnant women | WB | 2425 | 1.07 |
| Chen, 2007 | 2002 | Eastern | Outpatient clinics | CS | Conv | Pregnant women | ELISA | 504 | 10.71 |
| Chen, 2010b | 2008 | Eastern | Community | CS | RS | Healthy adults/children/adolescent aged 1-5 years | ELISA | 49 | 10.20 |
| Chen, 2010b | 2008 | Eastern | Community | CS | RS | Healthy adults/children/adolescent aged 5-10 years | ELISA | 115 | 0.00 |
| Chen, 2010b | 2008 | Eastern | Community | CS | RS | Healthy adults/children/adolescent aged 10-15 years | ELISA | 109 | 0.00 |
| Chen, 2010b | 2008 | Eastern | Community | CS | RS | Healthy adults/children/adolescent aged 15-20 years | ELISA | 167 | 7.19 |
| Chen, 2010b | 2008 | Eastern | Community | CS | RS | Healthy adults/children/adolescent aged 20-25 years | ELISA | 194 | 10.31 |
| Chen, 2010b | 2008 | Eastern | Community | CS | RS | Healthy adults/children/adolescent aged 25-30years | ELISA | 247 | 9.72 |
| Chen, 2010b | 2008 | Eastern | Community | CS | RS | Healthy adults/children/adolescent aged 30-35 years | ELISA | 289 | 11.76 |
| Chen, 2010b | 2008 | Eastern | Community | CS | RS | Healthy adults/children/adolescent aged 35-40 years | ELISA | 298 | 15.10 |
| Chen, 2010b | 2008 | Eastern | Community | CS | RS | Healthy adults/children/adolescent aged 40-45 years | ELISA | 247 | 15.79 |
| Chen, 2010b | 2008 | Eastern | Community | CS | RS | Healthy adults/children/adolescent aged 45-50 years | ELISA | 186 | 24.19 |
| Chen, 2010b | 2008 | Eastern | Community | CS | RS | Healthy adults/children/adolescent aged 50-55 years | ELISA | 183 | 12.57 |
| Chen, 2010b | 2008 | Eastern | Community | CS | RS | Healthy adults/children/adolescent aged 55-60 years | ELISA | 106 | 16.04 |
| Chen, 2010c | 2007-09 | Eastern | Outpatient clinics | CS | Conv | Healthy adults/children/adolescent aged 19-25 years | ELISA | 230 | 9.13 |
| Chen, 2010c | 2007-09 | Eastern | Outpatient clinics | CS | Conv | Healthy adults/children/adolescent aged 26-30 years | ELISA | 763 | 6.42 |
| Chen, 2010c | 2007-09 | Eastern | Outpatient clinics | CS | Conv | Healthy adults/children/adolescent aged 31-43 years | ELISA | 386 | 7.25 |
| Chen, 2012 | 2009-11 | Southwestern | Outpatient clinics | CS | Conv | Women of childbearing age before or during early pregnancy | ELISA | 3829 | 0.63 |
| Chen, 2013b | 2001-09 | Eastern | Outpatient clinics | CS | Conv | Healthy adults aged 18-25 years | ELISA | 643 | 0.31 |
| Chen, 2013b | 2001-09 | Eastern | Outpatient clinics | CS | Conv | Healthy adults aged 26-32 years | ELISA | 2677 | 0.19 |
| Chen, 2013b | 2001-09 | Eastern | Outpatient clinics | CS | Conv | Healthy adults aged 33-39 years | ELISA | 1272 | 0.31 |
| Chen, 2013b | 2001-09 | Eastern | Outpatient clinics | CS | Conv | Healthy adults aged 40-45 years | ELISA | 206 | 0.00 |
| Chen, 2014 | 2009-13 | Eastern | Outpatient clinics | CS | Conv | Married women of childbearing age | ELISA | 22913 | 6.44 |
| Chen, 2019 | 2016-17 | North | Outpatient clinics | Cohort | Conv | Women before pregnancy | ELISA | 10669 | 4.75 |
| Chen, 2022 | 2018-20 | Eastern | Outpatient clinics | CS | Conv | Healthy adults aged <35 years | IF | 2235 | 10.69 |
| Chen, 2022 | 2018-20 | Eastern | Outpatient clinics | CS | Conv | Healthy adults aged ≥35 years | IF | 835 | 14.01 |
| Cui, 2016 | 2008-15 | North | Outpatient clinics | CS | Conv | Pregnant women | ELISA | 1063 | 9.88 |
| Cui, 2016 | 2008-15 | North | Outpatient clinics | CS | Conv | Infertile women | ELISA | 189 | 15.34 |
| Cui, 2016 | 2008-15 | North | Outpatient clinics | CS | Conv | Women with a history of poor pregnancy | ELISA | 1434 | 9.55 |
| Cui, 2016 | 2008-15 | North | Outpatient clinics | CS | Conv | Women with recent abnormal pregnancies | ELISA | 147 | 10.20 |
| Cui, 2016 | 2008-15 | North | Outpatient clinics | CS | Conv | Examine the woman before pregnancy | ELISA | 71 | 8.45 |
| Cui, 2016 | 2008-15 | North | Outpatient clinics | CS | Conv | Health women | ELISA | 694 | 17.44 |
| Dai, 2009 | 2006 | Central-southern | Other | CS | Conv | Migrant female workers | ELISA | 295 | 14.92 |
| Deng, 1999 | 1994-95 | Eastern | Outpatient clinics | CS | RS | Pregnant women | ELISA | 335 | 48.06 |
| Deng, 2015 | 2013-14 | Central-southern | Outpatient clinics | CS | Conv | Pregnant women | ELISA | 2760 | 17.86 |
| Ding, 2015 | 2011-13 | Eastern | Outpatient clinics | CS | Conv | Pregnant women | ELISA | 3090 | 10.00 |
| Ding, 2015 | 2011-13 | Eastern | Outpatient clinics | CS | Conv | Maternal women | ELISA | 1868 | 16.70 |
| Dong, 1998 | － | Multilple or unknown | Outpatient clinics | CS | Conv | Childbirth pregnant women | ELISA | 233 | 68.24 |
| Dong, 2000 | － | North | Outpatient clinics | CS | Conv | Early pregnant women | ELISA | 282 | 12.77 |
| Duan, 2010 | 2006 | Southwestern | Community | CS | Conv | Hani population | ELISA | 1001 | 43.76 |
| Duan, 2011 | 2007-11 | Eastern | Outpatient clinics | CS | Conv | Preconception women | ELISA | 958 | 0.10 |
| Fan, 2008 | 2005-06 | Central-southern | Outpatient clinics | CS | Conv | Pregnant women | ELISA | 3650 | 8.60 |
| Feng, 2014 | 2011-13 | Central-southern | Outpatient clinics | CS | Conv | Pregnant women | ELISA | 3072 | 1.43 |
| Fu, 2015 | 2011-12 | Central-southern | Outpatient clinics | CS | Conv | Pregnant women | ELISA | 1073 | 1.68 |
| Gao, 2014 | 2012-13 | Central-southern | Outpatient clinics | CS | Conv | Pregnant women | ELISA | 530 | 11.32 |
| Gao, 2021 | 2019 | Southwestern | Other | CS | Conv | Healthy unpaid blood donors aged 18-24 years | ELISA | 172 | 4.65 |
| Gao, 2021 | 2019 | Southwestern | Other | CS | Conv | 25–34 Healthy unpaid blood donors | ELISA | 349 | 4.87 |
| Gao, 2021 | 2019 | Southwestern | Other | CS | Conv | Healthy unpaid blood donors aged 35-44 years | ELISA | 345 | 6.38 |
| Gao, 2021 | 2019 | Southwestern | Other | CS | Conv | Healthy unpaid blood donors aged 45-60 years | ELISA | 433 | 4.39 |
| Gong, 2005 | 2001-03 | Central-southern | Outpatient clinics | CS | Conv | Pregnant women | ELISA | 1912 | 7.37 |
| Guan, 2010a | 2008-09 | Central-southern | Outpatient clinics | CS | Conv | Pregnant women | ELISA | 2862 | 7.79 |
| Guan, 2010b | 2005-07 | Northeastern | Outpatient clinics | CC | Conv | Cases inpatients with AMI | ELISA | 102 | 54.90 |
| Guan, 2010b | 2005-07 | Northeastern | Outpatient clinics | CC | RS | Control 1s who visited the hospital for | ELISA | 150 | 38.00 |
| Guo, 2008a | 2005-07 | Central-southern | Outpatient clinics | CS | Conv | Pregnant women | ELISA | 890 | 1.57 |
| Guo, 2008b | 2005-07 | Southwestern | Outpatient clinics | CS | Conv | Pregnancy | ELISA | 5219 | 0.33 |
| Guo, 2009a | 2006-07 | North | Outpatient clinics | CS | Conv | Pregnant women | ELISA | 1051 | 1.05 |
| Guo, 2009b | 2006-07 | Central-southern | Outpatient clinics | CS | Conv | Pregnancy | ELISA | 1982 | 8.93 |
| Guo, 2010 | 2000-07 | Central-southern | Community | CS | Conv | Women of childbearing age | ELISA | 35620 | 0.52 |
| Han, 2002 | － | Northeastern | Outpatient clinics | CS | Conv | Pregnancy | ELISA | 532 | 3.95 |
| Han, 2021 | 2016 | Northwestern | Community | CS | MSCS | Non-pregnant women with history of sexual intercourse | Other | 10038 | 23.59 |
| He, 2005 | 1998-03 | Southwestern | Outpatient clinics | CS | Conv | Pre-pregnancy and antenatal females | ELISA | 1949 | 10.42 |
| He, 2009 | － | Eastern | Community | CS | Conv | Male migrant workers aged <25 years | ELISA | 179 | 3.91 |
| He, 2009 | － | Eastern | Community | CS | Conv | 26-35 years ols male migrant workers | ELISA | 419 | 5.25 |
| He, 2009 | － | Eastern | Community | CS | Conv | >36 years old male migrant workers | ELISA | 296 | 6.76 |
| He, 2014 | 2011-13 | Southwestern | Community | CS | Conv | Antenatal women | ELISA | 782 | 3.45 |
| Hu, 2007 | － | Southwestern | Outpatient clinics | CS | Conv | Infertility | ELISA | 185 | 74.59 |
| Hu, 2012 | 2009-11 | Central-southern | Outpatient clinics | CS | Conv | Pregnancy | ELISA | 410 | 13.66 |
| Hu, 2014 | 2009-13 | Eastern | Community | CS | Conv | Women of childbearing age before pregnancy | ELISA | 1523 | 7.16 |
| Hu, 2021 | 2017-19 | Eastern | Outpatient clinics | CS | Conv | Male 28days -17 | Other | 405 | 6.91 |
| Hu, 2021 | 2017-19 | Eastern | Outpatient clinics | CS | Conv | Male 18-29 | Other | 460 | 6.74 |
| Hu, 2021 | 2017-19 | Eastern | Outpatient clinics | CS | Conv | Male 30-39 | Other | 460 | 11.96 |
| Hu, 2021 | 2017-19 | Eastern | Outpatient clinics | CS | Conv | Male 40-49 | Other | 356 | 20.79 |
| Hu, 2021 | 2017-19 | Eastern | Outpatient clinics | CS | Conv | Male 50-59 | Other | 318 | 22.96 |
| Hu, 2021 | 2017-19 | Eastern | Outpatient clinics | CS | Conv | Male >=60 | Other | 497 | 14.29 |
| Hu, 2021 | 2017-19 | Eastern | Outpatient clinics | CS | Conv | Female 28days -17 | Other | 304 | 4.93 |
| Hu, 2021 | 2017-19 | Eastern | Outpatient clinics | CS | Conv | Female 18-29 | Other | 9310 | 8.70 |
| Hu, 2021 | 2017-19 | Eastern | Outpatient clinics | CS | Conv | Female 30-39 | Other | 6495 | 16.35 |
| Hu, 2021 | 2017-19 | Eastern | Outpatient clinics | CS | Conv | Female 40-49 | Other | 748 | 25.27 |
| Hu, 2021 | 2017-19 | Eastern | Outpatient clinics | CS | Conv | Female 50-59 | Other | 237 | 24.47 |
| Hu, 2021 | 2017-19 | Eastern | Outpatient clinics | CS | Conv | Female >=60 | Other | 3399 | 1.38 |
| Huai, 2019 | 2016 | Eastern | Community | CS | MSCS | 18-24 years old males | ELISA | 251 | 2.39 |
| Huai, 2019 | 2016 | Eastern | Community | CS | MSCS | 25-29 years old males | ELISA | 786 | 1.53 |
| Huai, 2019 | 2016 | Eastern | Community | CS | MSCS | 30-34 years old males | ELISA | 503 | 1.99 |
| Huai, 2019 | 2016 | Eastern | Community | CS | MSCS | 35-39 years old males | ELISA | 566 | 3.36 |
| Huai, 2019 | 2016 | Eastern | Community | CS | MSCS | 40-44 years old males | ELISA | 721 | 3.74 |
| Huai, 2019 | 2016 | Eastern | Community | CS | MSCS | 45-49 years old males | ELISA | 697 | 2.01 |
| Huai, 2019 | 2016 | Eastern | Community | CS | MSCS | 18-24 years old females | ELISA | 195 | 4.62 |
| Huai, 2019 | 2016 | Eastern | Community | CS | MSCS | 25-29 years old females | ELISA | 914 | 3.39 |
| Huai, 2019 | 2016 | Eastern | Community | CS | MSCS | 30-34 years old females | ELISA | 559 | 5.90 |
| Huai, 2019 | 2016 | Eastern | Community | CS | MSCS | 35-39 years old females | ELISA | 591 | 4.91 |
| Huai, 2019 | 2016 | Eastern | Community | CS | MSCS | 40-44 years old females | ELISA | 742 | 4.85 |
| Huai, 2019 | 2016 | Eastern | Community | CS | MSCS | 45-49 years old females | ELISA | 729 | 3.84 |
| Huang, 2010 | 2008-09 | Northwestern | Outpatient clinics | CS | Conv | Pregnancy | ELISA | 3100 | 0.35 |
| Huang, 2015a | 2013-14 | Northwestern | Outpatient clinics | CS | Conv | Pregnancy in 2013 | ELISA | 3284 | 0.03 |
| Huang, 2015a | 2013-14 | Northwestern | Outpatient clinics | CS | Conv | Pregnancy in 2014 | ELISA | 2163 | 0.14 |
| Huang, 2015b | 2010-14 | Northwestern | Outpatient clinics | CS | Conv | Pregnancy | ELISA | 569 | 6.68 |
| Huang, 2015d | 2013-14 | Eastern | Outpatient clinics | CS | Conv | Healthy adults | ELISA | 2560 | 72.97 |
| Huang, 2020 | 2015-18 | Central-southern | Outpatient clinics | CS | Conv | Pregnancy aged <20 years | ELISA | 257 | 5.06 |
| Huang, 2020 | 2015-18 | Central-southern | Outpatient clinics | CS | Conv | Pregnancy aged 20-30 years | ELISA | 1490 | 4.90 |
| Huang, 2020 | 2015-18 | Central-southern | Outpatient clinics | CS | Conv | Pregnancy aged ≥30 years | ELISA | 514 | 4.28 |
| Jiang, 2003 | 1999-01 | Southwestern | Outpatient clinics | CS | Conv | Pregnancy | ELISA | 4274 | 1.24 |
| Jiang, 2011 | 2011 | Eastern | Outpatient clinics | CS | Conv | Pregnancy | ELISA | 800 | 12.13 |
| Jiang, 2019a | 2016-17 | Central-southern | Outpatient clinics | CS | Conv | Healthy adults aged <20 years | Other | 68 | 0.00 |
| Jiang, 2019a | 2016-17 | Central-southern | Outpatient clinics | CS | Conv | Healthy adults aged 20-34 years | Other | 3709 | 1.13 |
| Jiang, 2019a | 2016-17 | Central-southern | Outpatient clinics | CS | Conv | Healthy adults aged ≥35 years | Other | 795 | 0.88 |
| Jiang, 2019b | 2017 | Central-southern | Outpatient clinics | CS | Conv | Healthy adults aged <20 years | Other | 13 | 7.69 |
| Jiang, 2019b | 2017 | Central-southern | Outpatient clinics | CS | Conv | Healthy adults aged 20-29 years | Other | 695 | 1.29 |
| Jiang, 2019b | 2017 | Central-southern | Outpatient clinics | CS | Conv | Healthy adults aged 30-39 years | Other | 289 | 0.69 |
| Jiang, 2019b | 2017 | Central-southern | Outpatient clinics | CS | Conv | Healthy adults aged 40-44 years | Other | 9 | 0.00 |
| Jin, 1995 | 1984-95 | Northwestern | Outpatient clinics | CS | Conv | Pregnancy | ELISA | 959 | 3.02 |
| Jin, 2016 | 2015 | North | Outpatient clinics | CS | Conv | Women of childbearing age | ELISA | 970 | 14.64 |
| Jin, 2021 | 2019-20 | Southwestern | Outpatient clinics | CS | Conv | Healthy adults aged <35 years | Other | 1725 | 0.58 |
| Jin, 2021 | 2019-20 | Southwestern | Outpatient clinics | CS | Conv | Healthy adults aged ≥35 years | Other | 1340 | 0.07 |
| Lai, 2006 | 2000-04 | Central-southern | Outpatient clinics | CS | Conv | Pregnancy | ELISA | 8418 | 2.40 |
| Lai, 2023 | 2018-21 | Eastern | Outpatient clinics | CS | Conv | Healthy children/adolescent aged 1-3 years | IF | 373 | 12.33 |
| Lai, 2023 | 2018-21 | Eastern | Outpatient clinics | CS | Conv | Healthy children/adolescent aged 3-14 years | IF | 927 | 7.77 |
| Li, 2002b | 1996-00 | Northwestern | Outpatient clinics | CS | Conv | Pregnancy | ELISA | 1768 | 3.51 |
| Li, 2003a | － | Northeastern | Outpatient clinics | CS | Conv | Pregnancy | ELISA | 2184 | 2.38 |
| Li, 2005a | 2004-05 | Central-southern | Outpatient clinics | CS | RS | Pregnancy aged 20-25 years | ELISA | 74 | 16.22 |
| Li, 2005a | 2004-05 | Central-southern | Outpatient clinics | CS | RS | Pregnancy aged 26-30 years | ELISA | 270 | 31.11 |
| Li, 2005a | 2004-05 | Central-southern | Outpatient clinics | CS | RS | Pregancy aged 31-35 years | ELISA | 130 | 24.62 |
| Li, 2005a | 2004-05 | Central-southern | Outpatient clinics | CS | RS | Pregancy aged 36-38 years | ELISA | 46 | 17.39 |
| Li, 2005b | － | Central-southern | Outpatient clinics | CS | Conv | Sterile women | ELISA | 148 | 16.89 |
| Li, 2005c | 2001-03 | Central-southern | Outpatient clinics | CS | Conv | Pregnancy | ELISA | 1912 | 7.37 |
| Li, 2006a | 2004-06 | Central-southern | Outpatient clinics | CS | Conv | Pregnancy aged 21-25 years | ELISA | 412 | 18.45 |
| Li, 2006a | 2004-06 | Central-southern | Outpatient clinics | CS | Conv | Pregnancy aged 26-30 years | ELISA | 878 | 27.79 |
| Li, 2006a | 2004-06 | Central-southern | Outpatient clinics | CS | Conv | Pregancy aged 31-35 years | ELISA | 330 | 21.52 |
| Li, 2006a | 2004-06 | Central-southern | Outpatient clinics | CS | Conv | Pregancy aged 36-39 years | ELISA | 120 | 15.83 |
| Li, 2009 | 2008 | Eastern | Community | CS | Conv | Pre-pregnant women in Yutai | Other | 4880 | 0.25 |
| Li, 2009 | 2008 | Eastern | Community | CS | Conv | Pre-pregnant women in Jinxiang | Other | 2930 | 0.24 |
| Li, 2009 | 2008 | Eastern | Community | CS | Conv | Pre-pregnant women in Zoucheng | Other | 2118 | 0.57 |
| Li, 2010 | 2006-09 | Southwestern | Outpatient clinics | CS | Conv | Women of childbearing age | ELISA | 2695 | 26.09 |
| Li, 2011 | 2004-06 | Multilple or unknown | Outpatient clinics | CS | Conv | 20-25 years old pregnant women | ELISA | 412 | 18.45 |
| Li, 2011 | 2004-06 | Multilple or unknown | Outpatient clinics | CS | Conv | 26-30 years old preganat women | ELISA | 878 | 27.79 |
| Li, 2011 | 2004-06 | Multilple or unknown | Outpatient clinics | CS | Conv | 31-35 years old pregnant women | ELISA | 330 | 21.52 |
| Li, 2011 | 2004-06 | Multilple or unknown | Outpatient clinics | CS | Conv | 36-39 years old preganat women | ELISA | 120 | 15.83 |
| Li, 2012d | 2011-12 | Eastern | Outpatient clinics | CS | Conv | Women of childbearing age | ELISA | 37 | 18.92 |
| Li, 2012e | 2009-11 | Central-southern | Outpatient clinics | CS | Conv | Women of childbearing age who had both TORCH IgM and IgG tests | ELISA | 2320 | 71.03 |
| Li, 2012e | 2009-11 | Central-southern | Outpatient clinics | CS | Conv | Women of childbearing age who had only TORCH IgM test | ELISA | 1320 | 0.91 |
| Li, 2012f | 2011 | Eastern | Outpatient clinics | CS | Conv | Women of childbearing age | ELISA | 2334 | 85.30 |
| Li, 2012g | 2010-11 | Eastern | Outpatient clinics | CS | Conv | Pre-pregnant women | IF | 848 | 2.59 |
| Li, 2012g | 2010-11 | Eastern | Outpatient clinics | CS | Conv | Pregnancy | IF | 357 | 2.52 |
| Li, 2013a | 2011 | Central-southern | Outpatient clinics | CS | Conv | Pregnancy women | ELISA | 2535 | 0.16 |
| Li, 2014b | 2011-12 | Eastern | Outpatient clinics | CS | Conv | Women of childbearing age | ELISA | 4644 | 0.06 |
| Li, 2016a | 2014-16 | Southwestern | Outpatient clinics | CS | Conv | Pregnancy | IF | 1286 | 13.22 |
| Li, 2016b | 2014-15 | Central-southern | Outpatient clinics | CS | Conv | Pregnancy | ELISA | 1938 | 4.44 |
| Li, 2016c | 2013-14 | North | Community | CS | Conv | Healthy women before and during pregnancy | ELISA | 1050 | 16.10 |
| LI, 2017a | 2015-16 | Eastern | Outpatient clinics | CS | RS | Pregnancy | ELISA | 574 | 0.00 |
| Li, 2017b | － | Eastern | Outpatient clinics | CS | Conv | Pregnancy women | IF | 273 | 6.96 |
| Li, 2018a | 2016-18 | Central-southern | Inpatient | CS | Conv | Healthy adults aged 20.0-23.9 years | ELISA | 24 | 16.67 |
| Li, 2018a | 2016-18 | Central-southern | Inpatient | CS | Conv | Healthy adults aged 24.0-27.9 years | ELISA | 53 | 18.87 |
| Li, 2018a | 2016-18 | Central-southern | Inpatient | CS | Conv | Healthy adults aged >28.0 years | ELISA | 15 | 20.00 |
| Li, 2018b | 2014-15 | Central-southern | Community | CS | Conv | Healthy adults living inYiyang | ELISA | 3124 | 16.26 |
| Li, 2018b | 2014-15 | Central-southern | Community | CS | Conv | Healthy adults living in Changsha | ELISA | 2387 | 10.72 |
| Li, 2018b | 2014-15 | Central-southern | Community | CS | Conv | Healthy adults living in Yueyang | ELISA | 2222 | 18.86 |
| Li, 2018b | 2014-15 | Central-southern | Community | CS | Conv | Healthy adults living in Zhuzhou | ELISA | 2179 | 20.74 |
| Li, 2018b | 2014-15 | Central-southern | Community | CS | Conv | Healthy adults living in Chenzhou | ELISA | 2077 | 12.57 |
| Li, 2018b | 2014-15 | Central-southern | Community | CS | Conv | Healthy adults living in Shaoyang | ELISA | 1325 | 11.85 |
| Li, 2018b | 2014-15 | Central-southern | Community | CS | Conv | Healthy adults living in Huaihua | ELISA | 774 | 12.27 |
| Li, 2018b | 2014-15 | Central-southern | Community | CS | Conv | Healthy adults living in Hengyang | ELISA | 505 | 15.05 |
| Li, 2018b | 2014-15 | Central-southern | Community | CS | Conv | Healthy adults living in Loudi | ELISA | 302 | 14.24 |
| Li, 2018b | 2014-15 | Central-southern | Community | CS | Conv | Healthy adults living in Yongzhou | ELISA | 267 | 13.11 |
| Li, 2018b | 2014-15 | Central-southern | Community | CS | Conv | Healthy adults living in Changde | ELISA | 193 | 15.03 |
| Li, 2018b | 2014-15 | Central-southern | Community | CS | Conv | Healthy adults living in Xiangtan | ELISA | 161 | 14.91 |
| Li, 2018b | 2014-15 | Central-southern | Community | CS | Conv | Healthy adults living in Jishou | ELISA | 157 | 12.10 |
| Li, 2018c | 2013-17 | North | Outpatient clinics | CS | Conv | Pregnancy | ELISA | 2617 | 8.75 |
| Liang, 2017 | 2016 | Eastern | Outpatient clinics | CS | Conv | Women of child-bearing age | IF | 3866 | 11.61 |
| Liang, 2020 | 2017-18 | Central-southern | Outpatient clinics | CS | Conv | Healthy adults aged <35 years | IF | 2875 | 6.64 |
| Liang, 2020 | 2017-18 | Central-southern | Outpatient clinics | CS | Conv | Healthy adults aged ≥35 years | IF | 991 | 7.37 |
| Lin, 2000 | 1996-97 | Eastern | Outpatient clinics | CS | RS | Pregnancy women | Other | 6000 | 4.13 |
| Lin, 2002 | 1999-00 | Central-southern | Outpatient clinics | CS | Conv | Health adults | Other | 180 | 50.00 |
| Lin, 2009a | 2007-09 | Central-southern | Outpatient clinics | CS | Conv | Pregnancy women | ELISA | 476 | 15.34 |
| Lin, 2011 | 2006 | Eastern | Community | CS | CRS | 5-9 years old males | ELISA | 75 | 0.00 |
| Lin, 2011 | 2006 | Eastern | Community | CS | CRS | 5-9 years old females | ELISA | 40 | 0.00 |
| Lin, 2011 | 2006 | Eastern | Community | CS | CRS | Males aged 10-14 years | ELISA | 64 | 0.00 |
| Lin, 2011 | 2006 | Eastern | Community | CS | CRS | Females aged 10-14 years | ELISA | 45 | 0.00 |
| Lin, 2011 | 2006 | Eastern | Community | CS | CRS | 15-19 years old males | ELISA | 89 | 5.62 |
| Lin, 2011 | 2006 | Eastern | Community | CS | CRS | 15-19 yeasr old females | ELISA | 78 | 8.97 |
| Lin, 2011 | 2006 | Eastern | Community | CS | CRS | 20-24 years old males | ELISA | 93 | 8.60 |
| Lin, 2011 | 2006 | Eastern | Community | CS | CRS | 20-24 years old females | ELISA | 101 | 11.88 |
| Lin, 2011 | 2006 | Eastern | Community | CS | CRS | 25-29 years old males | ELISA | 112 | 5.36 |
| Lin, 2011 | 2006 | Eastern | Community | CS | CRS | 25-29 years old females | ELISA | 135 | 13.33 |
| Lin, 2011 | 2006 | Eastern | Community | CS | CRS | 30-34 years old males | ELISA | 137 | 10.22 |
| Lin, 2011 | 2006 | Eastern | Community | CS | CRS | 30-34 years old females | ELISA | 152 | 13.16 |
| Lin, 2011 | 2006 | Eastern | Community | CS | CRS | 35-39 years old males | ELISA | 144 | 13.89 |
| Lin, 2011 | 2006 | Eastern | Community | CS | CRS | 35-39 years old females | ELISA | 154 | 16.23 |
| Lin, 2011 | 2006 | Eastern | Community | CS | CRS | 40-44 years old males | ELISA | 118 | 13.56 |
| Lin, 2011 | 2006 | Eastern | Community | CS | CRS | 40-44 years old females | ELISA | 129 | 17.83 |
| Lin, 2011 | 2006 | Eastern | Community | CS | CRS | 45-49 year sold males | ELISA | 89 | 23.60 |
| Lin, 2011 | 2006 | Eastern | Community | CS | CRS | 45-49 year sold females | ELISA | 97 | 24.74 |
| Lin, 2011 | 2006 | Eastern | Community | CS | CRS | 50-54 years old males | ELISA | 82 | 8.54 |
| Lin, 2011 | 2006 | Eastern | Community | CS | CRS | 50-54 years old females | ELISA | 101 | 15.84 |
| Lin, 2011 | 2006 | Eastern | Community | CS | CRS | 55-60 years old males | ELISA | 62 | 16.13 |
| Lin, 2011 | 2006 | Eastern | Community | CS | CRS | 55-60 years old females | ELISA | 44 | 15.91 |
| Lin, 2013 | 2012-13 | Central-southern | Outpatient clinics | CS | Conv | Child-bearing age and pregnant women | ELISA | 2368 | 12.92 |
| Lin, 2017 | 2016 | Central-southern | Outpatient clinics | CS | Conv | Pregnancy women | ELISA | 400 | 11.50 |
| Lin, 2019 | 2016-17 | Northeastern | Outpatient clinics | CS | Conv | Healthy adults aged <35 years | IF | 1104 | 8.88 |
| Lin, 2019 | 2016-17 | Northeastern | Outpatient clinics | CS | Conv | Healthy adults aged ≥35 years | IF | 939 | 16.19 |
| Lin, 2022a | 2021-22 | Eastern | Outpatient clinics | CS | Conv | Women of child-bearing age | ELISA | 60 | 3.33 |
| Lin, 2022b | 2019-21 | Central-southern | Outpatient clinics | CS | Conv | Healthy adults aged ≤25 years | IF | 583 | 8.75 |
| Lin, 2022b | 2019-21 | Central-southern | Outpatient clinics | CS | Conv | Healthy adults aged 26-34 years | IF | 991 | 6.66 |
| Lin, 2022b | 2019-21 | Central-southern | Outpatient clinics | CS | Conv | Healthy adults aged ≥35 years | IF | 346 | 7.51 |
| Liu, 2008 | 2004-05 | Eastern | Community | CS | Conv | Pre-pregnancy women | Other | 20000 | 0.79 |
| Liu, 2009a | 2008-09 | Eastern | Outpatient clinics | CS | Conv | Pregnancy women | ELISA | 2525 | 0.67 |
| Liu, 2014 | 2012-13 | North | Outpatient clinics | CS | Conv | Pregnancy women | ELISA | 105 | 2.86 |
| Liu, 2015 | 2013 | Northwestern | Community | CS | CRS | Women of child-bearing age | ELISA | 550 | 15.82 |
| Liu, 2017c | 2013-16 | Central-southern | Outpatient clinics | CS | Conv | Healthy adults aged <35 years | ELISA | 5876 | 4.22 |
| Liu, 2017c | 2013-16 | Central-southern | Outpatient clinics | CS | Conv | Healthy adults aged ≥35 years | ELISA | 1246 | 9.63 |
| Liu, 2020 | 2015-19 | North | Outpatient clinics | CS | Conv | Healthy adults aged <35 years | IF | 1146 | 4.10 |
| Liu, 2020 | 2015-19 | North | Outpatient clinics | CS | Conv | Healthy adults aged ≥35 years | IF | 179 | 9.50 |
| Lo, 1999 | 1995 | SARs and Taiwan | Outpatient clinics | CS | Conv | >25 years old females from general population | WB | 76 | 18.42 |
| Lo, 1999 | 1995 | SARs and Taiwan | Outpatient clinics | CS | Conv | >25 years old males from general population | WB | 75 | 17.33 |
| Lo, 1999 | 1995 | SARs and Taiwan | Outpatient clinics | CS | Conv | Antenatal women in Hong kong | WB | 78 | 12.82 |
| Lo, 1999 | 1995 | SARs and Taiwan | Outpatient clinics | CS | Conv | Antenatal women in southren China | WB | 62 | 3.23 |
| Lu, 2001 | － | Central-southern | Community | CS | Conv | Women of child-bearing age | ELISA | 1358 | 3.98 |
| Lu, 2002 | 2000-01 | Central-southern | Other | CS | Conv | Blood donors | ELISA | 91 | 8.79 |
| Luo, 2002b | 1999-01 | Southwestern | Outpatient clinics | CS | Conv | Pregnancy women | ELISA | 1751 | 0.46 |
| Luo, 2011a | 2008-10 | Central-southern | Outpatient clinics | CS | Conv | Pregnancy women | ELISA | 5086 | 0.79 |
| Luo, 2016 | 2011-14 | Eastern | Outpatient clinics | CS | Conv | Women of child-bearing age | IF | 5985 | 0.27 |
| Ma, 2006 | 2003-05 | Central-southern | Outpatient clinics | CS | Conv | Pregnancy women | ELISA | 1810 | 44.97 |
| Ma, 2007 | 2000-07 | Northwestern | Outpatient clinics | CS | Conv | Pregnancy women | ELISA | 5859 | 0.05 |
| Ma, 2019 | 2015-17 | Southwestern | Outpatient clinics | CS | Conv | Pregnancy women | ELISA | 1022 | 11.35 |
| Mei, 2017 | 2013-16 | Eastern | Outpatient clinics | CS | Conv | Healthy adults aged ≤29 years | ELISA | 1305 | 8.58 |
| Mei, 2017 | 2013-16 | Eastern | Outpatient clinics | CS | Conv | Healthy adults aged 30-34 years | ELISA | 517 | 16.05 |
| Mei, 2017 | 2013-16 | Eastern | Outpatient clinics | CS | Conv | Healthy adults aged ≥35 years | ELISA | 150 | 21.33 |
| Nie, 2005 | 2004-05 | Eastern | Outpatient clinics | CS | Conv | Pregnancy women | ELISA | 3700 | 0.43 |
| Nie, 2015 | 2012-14 | North | Outpatient clinics | CS | Conv | Pregnancy women | ELISA | 2365 | 0.42 |
| Nong, 2002 | 1998-01 | Central-southern | Outpatient clinics | CS | Conv | Pregnancy women | ELISA | 1285 | 2.49 |
| Pan, 2016a | 2013-14 | Southwestern | Outpatient clinics | CS | Conv | Pregnancy women | ELISA | 4371 | 1.08 |
| Pan, 2016b | 2011-13 | Central-southern | Outpatient clinics | CS | Conv | Pregnancy women | ELISA | 600 | 0.50 |
| Pang, 2012 | 2010-11 | Central-southern | Outpatient clinics | CS | Conv | Pregnant women of childbearing age | ELISA | 2617 | 4.74 |
| Pang, 2016 | － | Central-southern | Outpatient clinics | CS | Conv | Pregnant women | IF | 2002 | 8.19 |
| Peng, 1991 | 1987-88 | Southwestern | Outpatient clinics | CC | Conv | women without invasive cervical cancer | WB | 142 | 28.87 |
| Peng, 2007 | 2005-06 | Eastern | Outpatient clinics | CS | Conv | Pregnant women | ELISA | 1716 | 1.98 |
| Peng, 2013 | 2011-12 | Southwestern | Outpatient clinics | CS | Conv | Pregnant women | ELISA | 1358 | 17.82 |
| Qi, 2004 | － | Eastern | Outpatient clinics | CS | Conv | Conscripted male youth for re-examination | ELISA | 356 | 15.17 |
| Qian, 2016 | 2013-15 | Central-southern | Outpatient clinics | CS | Conv | <35 years old pregnant women | IF | 1728 | 0.58 |
| Qian, 2016 | 2013-15 | Central-southern | Outpatient clinics | CS | Conv | Pregnant women aged ≥35 years | IF | 628 | 1.43 |
| Qiao, 2010 | 2002-09 | Central-southern | Outpatient clinics | CS | Conv | Pregnant women | ELISA | 4538 | 8.13 |
| Qiao, 2013 | 2012 | North | Outpatient clinics | CS | Conv | Pregnant women | Other | 974 | 0.10 |
| Qin, 2006a | 2003-05 | Central-southern | Outpatient clinics | CS | Conv | Pregnant women | ELISA | 1530 | 1.83 |
| Qiu, 2009a | 2007 | Eastern | Community | CS | CRS | Male college Students | ELISA | 586 | 15.70 |
| Qiu, 2009a | 2007 | Eastern | Community | CS | CRS | Female college Students | ELISA | 534 | 17.42 |
| Qiu, 2009b | 2006-08 | Central-southern | Outpatient clinics | CS | Conv | Pregnant women | ELISA | 2862 | 7.79 |
| Qu, 1997 | 1994-96 | Northwestern | Outpatient clinics | CS | Conv | Pregnant women | ELISA | 430 | 84.19 |
| Qu, 2009 | 2006-08 | Central-southern | Outpatient clinics | CS | Conv | women of child-bearing age | ELISA | 2305 | 0.26 |
| Que, 2014 | 2014 | Central-southern | Outpatient clinics | CS | Conv | Pregnant women | ELISA | 978 | 14.42 |
| Ran, 2014 | 2013 | Southwestern | Outpatient clinics | CS | Conv | Women of childbearing age in the first 2 months of pregnancy and in the first 1-3 months of pregnancy | ELISA | 796 | 0.63 |
| Ren, 2023 | 2015-19 | North | Outpatient clinics | CS | Conv | <35 years old pregnant women | IF | 1556 | 5.21 |
| Ren, 2023 | 2015-19 | North | Outpatient clinics | CS | Conv | Pregnant women aged ≥35 years | IF | 233 | 10.30 |
| Ruan, 2014 | 2010-13 | Southwestern | Outpatient clinics | CS | Conv | 18-25 years old women of child-bearing age | Other | 482 | 3.53 |
| Ruan, 2014 | 2010-13 | Southwestern | Outpatient clinics | CS | Conv | 26-35 years old women of child-bearing age | Other | 611 | 3.11 |
| Ruan, 2014 | 2010-13 | Southwestern | Outpatient clinics | CS | Conv | 36-55 years old women of child-bearing age | Other | 783 | 3.19 |
| Shading, 2005 | 2003 | Northwestern | Outpatient clinics | CS | Conv | Pregnant women | ELISA | 185 | 1.08 |
| Shao, 2019 | 2015-16 | Eastern | Outpatient clinics | CS | Conv | Women of child-bearing age | Other | 825 | 6.30 |
| Shen, 2010 | 2007-09 | Central-southern | Outpatient clinics | CS | Conv | Women of child-bearing age | ELISA | 4860 | 0.62 |
| Shen, 2015 | 2007 | SARs and Taiwan | Community | CS | RS | <15 years of age men | ELISA | 163 | 0.00 |
| Shen, 2015 | 2007 | SARs and Taiwan | Community | CS | RS | 15-20 years old men | ELISA | 45 | 4.44 |
| Shen, 2015 | 2007 | SARs and Taiwan | Community | CS | RS | 20-25 years of age men | ELISA | 31 | 3.23 |
| Shen, 2015 | 2007 | SARs and Taiwan | Community | CS | RS | 25-30 years of age men | ELISA | 16 | 0.00 |
| Shen, 2015 | 2007 | SARs and Taiwan | Community | CS | RS | 30-35 years of age men | ELISA | 11 | 0.00 |
| Shen, 2015 | 2007 | SARs and Taiwan | Community | CS | RS | 35-40 years of age men | ELISA | 18 | 16.67 |
| Shen, 2015 | 2007 | SARs and Taiwan | Community | CS | RS | 40-45 years of age men | ELISA | 12 | 0.00 |
| Shen, 2015 | 2007 | SARs and Taiwan | Community | CS | RS | 45-50 years of age men | ELISA | 11 | 0.00 |
| Shen, 2015 | 2007 | SARs and Taiwan | Community | CS | RS | 15-20 years old men | ELISA | 13 | 15.38 |
| Shen, 2015 | 2007 | SARs and Taiwan | Community | CS | RS | 55-65 years of age men | ELISA | 21 | 14.29 |
| Shen, 2015 | 2007 | SARs and Taiwan | Community | CS | RS | 65-70 years of age men | ELISA | 29 | 17.24 |
| Shen, 2015 | 2007 | SARs and Taiwan | Community | CS | RS | 70-75 years of age men | ELISA | 20 | 15.00 |
| Shen, 2015 | 2007 | SARs and Taiwan | Community | CS | RS | >75 years of age men | ELISA | 19 | 42.11 |
| Shen, 2015 | 2007 | SARs and Taiwan | Community | CS | RS | <15 years of age women | ELISA | 162 | 1.23 |
| Shen, 2015 | 2007 | SARs and Taiwan | Community | CS | RS | 15-20 years of age women | ELISA | 70 | 1.43 |
| Shen, 2015 | 2007 | SARs and Taiwan | Community | CS | RS | 20-25 years of age women | ELISA | 26 | 3.85 |
| Shen, 2015 | 2007 | SARs and Taiwan | Community | CS | RS | 25-30 years of age women | ELISA | 50 | 4.00 |
| Shen, 2015 | 2007 | SARs and Taiwan | Community | CS | RS | 30-35 years of age women | ELISA | 63 | 11.11 |
| Shen, 2015 | 2007 | SARs and Taiwan | Community | CS | RS | 35-40 years of age women | ELISA | 37 | 8.11 |
| Shen, 2015 | 2007 | SARs and Taiwan | Community | CS | RS | 40-45 years of age women | ELISA | 30 | 13.33 |
| Shen, 2015 | 2007 | SARs and Taiwan | Community | CS | RS | 45-50 years of age women | ELISA | 47 | 21.28 |
| Shen, 2015 | 2007 | SARs and Taiwan | Community | CS | RS | 50-55 years of age women | ELISA | 38 | 21.05 |
| Shen, 2015 | 2007 | SARs and Taiwan | Community | CS | RS | 55-60 years of age women | ELISA | 27 | 14.81 |
| Shen, 2015 | 2007 | SARs and Taiwan | Community | CS | RS | 60-65 years of age women | ELISA | 35 | 37.14 |
| Shen, 2015 | 2007 | SARs and Taiwan | Community | CS | RS | 65-70 years of age women | ELISA | 39 | 33.33 |
| Shen, 2015 | 2007 | SARs and Taiwan | Community | CS | RS | >75 years of age women | ELISA | 39 | 38.46 |
| Sheng, 1999 | 1998 | Central-southern | Outpatient clinics | CS | Conv | Health women(22-40 years old) | Other | 170 | 48.82 |
| Sheng, 1999 | 1998 | Central-southern | Outpatient clinics | CS | Conv | Health children/adolescent(2 months～8 years old) | Other | 50 | 40.00 |
| Shi, 2012b | 2010-11 | Eastern | Outpatient clinics | CS | Conv | Pregnant women(20-41 years old) | ELISA | 2403 | 3.29 |
| Shi, 2012b | 2010-11 | Eastern | Outpatient clinics | CS | Conv | Health children/adolescent(0-3 years old) | ELISA | 357 | 1.96 |
| Shi, 2016a | 2016 | Eastern | Outpatient clinics | CS | Conv | Pregnant women | ELISA | 216 | 12.04 |
| Shi, 2016b | 2014-15 | Northwestern | Outpatient clinics | CS | Conv | Pregnant women in 2014 | ELISA | 5306 | 4.05 |
| Shi, 2016b | 2014-15 | Northwestern | Outpatient clinics | CS | Conv | Pregnant women in 2015 | ELISA | 5067 | 5.82 |
| Shu, 1997 | 1995-96 | Southwestern | Outpatient clinics | CS | Conv | 20-23.9 years old pregnant women | ELISA | 65 | 16.92 |
| Shu, 1997 | 1995-96 | Southwestern | Outpatient clinics | CS | Conv | 24-27.9 years old pregnant women | ELISA | 144 | 18.06 |
| Shu, 1997 | 1995-96 | Southwestern | Outpatient clinics | CS | Conv | ≥28 years old pregnant women | ELISA | 41 | 21.95 |
| Song, 2002 | 2000-01 | Northwestern | Outpatient clinics | CS | Conv | Pregnant women | ELISA | 2718 | 4.82 |
| Song, 2012 | 2011 | North | Outpatient clinics | CS | Conv | Pregnant women | ELISA | 693 | 9.96 |
| Sun, 2002 | 2000 | Eastern | Outpatient clinics | CS | Conv | ≤20 years old early pregnant women | Other | 26 | 0.00 |
| Sun, 2002 | 2000 | Eastern | Outpatient clinics | CS | Conv | 21-30 years old early pregnant women | Other | 374 | 2.94 |
| Sun, 2002 | 2000 | Eastern | Outpatient clinics | CS | Conv | 31-40 years old early pregnant women | Other | 69 | 2.90 |
| Sun, 2002 | 2000 | Eastern | Outpatient clinics | CS | Conv | ≥41years old early pregnant women | Other | 17 | 0.00 |
| Sun, 2011 | 2008-09 | Central-southern | Outpatient clinics | CS | Conv | Pregnant women | ELISA | 5172 | 1.22 |
| Sun, 2013 | 2009-11 | Central-southern | Outpatient clinics | CS | TLS | Pregnant women in 2009 | ELISA | 2503 | 21.53 |
| Sun, 2013 | 2009-11 | Central-southern | Outpatient clinics | CS | TLS | Pregnant women in 2010 | ELISA | 2965 | 27.76 |
| Sun, 2013 | 2009-11 | Central-southern | Outpatient clinics | CS | TLS | Pregnant women in 2011 | ELISA | 3629 | 35.82 |
| Tang, 2008 | 2005-06 | Central-southern | Community | CS | Conv | Mobile population | ELISA | 300 | 14.57 |
| Tang, 2008 | 2005-06 | Central-southern | Outpatient clinics | CS | Conv | Gynecology Clinic | ELISA | 280 | 18.66 |
| Tang, 2008 | 2005-06 | Central-southern | Community | CS | Conv | College entrance examination physical examination students | ELISA | 500 | 3.40 |
| Tang, 2009b | 2003-04 | Southwestern | Community | CS | MSCS | 20-24 years old rural married women | ELISA | 202 | 4.46 |
| Tang, 2009b | 2003-04 | Southwestern | Community | CS | MSCS | 25-29 years old rural married women | ELISA | 379 | 1.06 |
| Tang, 2009b | 2003-04 | Southwestern | Community | CS | MSCS | 30-34 years old rural married women | ELISA | 528 | 2.27 |
| Tang, 2009b | 2003-04 | Southwestern | Community | CS | MSCS | 35-39 years old rural married women | ELISA | 394 | 1.27 |
| Tang, 2009b | 2003-04 | Southwestern | Community | CS | MSCS | 40-44 years old rural married women | ELISA | 231 | 1.30 |
| Tang, 2009b | 2003-04 | Southwestern | Community | CS | MSCS | 45-49 years old rural married women | ELISA | 266 | 2.26 |
| Tang, 2011 | － | Southwestern | Community | CS | Conv | High school girls in school | ELISA | 856 | 60.05 |
| Tang, 2021 | 2019 | Southwestern | Outpatient clinics | CS | Conv | 19-25 years old women of child-bearing age | IF | 5239 | 1.05 |
| Tang, 2021 | 2019 | Southwestern | Outpatient clinics | CS | Conv | 26-35 years old women of child-bearing age | IF | 8045 | 0.89 |
| Tang, 2021 | 2019 | Southwestern | Outpatient clinics | CS | Conv | ≥36 years old women of child-bearing age | IF | 1989 | 0.75 |
| Tao, 2012 | 2009-11 | Southwestern | Outpatient clinics | CS | Conv | Newly married women of childbearing age | Other | 6215 | 0.14 |
| Tian, 2005 | 2003-04 | Southwestern | Outpatient clinics | CS | Conv | Pregnant women | ELISA | 560 | 2.32 |
| Wang, 2000 | 1995-98 | North | Outpatient clinics | CS | Conv | Pregnant women | ELISA | 886 | 1.02 |
| Wang, 2003 | 2001-02 | Eastern | Community | CS | Conv | Pre-pregnant woman | ELISA | 1791 | 2.51 |
| Wang, 2004 | 2003-04 | Eastern | Community | CS | Conv | Pregnant women | ELISA | 2052 | 1.12 |
| Wang, 2006a | 2004-06 | Central-southern | Outpatient clinics | CS | Conv | Pregnant women | ELISA | 521 | 3.26 |
| Wang, 2006b | 2003-05 | Northwestern | Outpatient clinics | CS | Conv | Pregnant women | ELISA | 2560 | 0.63 |
| Wang, 2008a | 2007-08 | Eastern | Outpatient clinics | CS | Conv | Pregnant women | ELISA | 124 | 5.65 |
| Wang, 2010 | 2008-09 | Central-southern | Outpatient clinics | CS | Conv | Pregnant women | ELISA | 1200 | 1.42 |
| Wang, 2012a | 2010 | Central-southern | Outpatient clinics | CS | Conv | Pregnant women | ELISA | 2456 | 0.90 |
| Wang, 2012b | 2010-11 | Central-southern | Outpatient clinics | CS | Conv | Pregnant women | ELISA | 1826 | 2.35 |
| Wang, 2012d | 2011-12 | Eastern | Outpatient clinics | CS | Conv | Pregnant women | ELISA | 1173 | 1.28 |
| Wang, 2013b | 2009-11 | Northwestern | Outpatient clinics | CS | Conv | Pregnant women in 2009 | ELISA | 6082 | 0.25 |
| Wang, 2013b | 2009-11 | Northwestern | Outpatient clinics | CS | Conv | Pregnant women in 2010 | ELISA | 5811 | 0.14 |
| Wang, 2013b | 2009-11 | Northwestern | Outpatient clinics | CS | Conv | Pregnant women in 2011 | ELISA | 6682 | 0.19 |
| Wang, 2013c | 2008-12 | Central-southern | Outpatient clinics | CS | Conv | Married woman | ELISA | 800 | 3.88 |
| Wang, 2017b | － | Northwestern | Community | CS | Conv | ≤20 years old Women of child-bearing age | ELISA | 18 | 11.11 |
| Wang, 2017b | － | Northwestern | Community | CS | Conv | 21-30 years old Women of child-bearing age | ELISA | 44 | 4.55 |
| Wang, 2017b | － | Northwestern | Community | CS | Conv | 31-40 years old Women of child-bearing age | ELISA | 57 | 5.26 |
| Wang, 2017b | － | Northwestern | Community | CS | Conv | 41-50 years old Women of child-bearing age | ELISA | 41 | 9.76 |
| Wang, 2017b | － | Northwestern | Community | CS | Conv | ≥51 years old Women of child-bearing age | ELISA | 34 | 5.88 |
| Wang, 2018a | 2015 | Northwestern | Outpatient clinics | CS | Conv | Pregnant women in 2015 | ELISA | 3339 | 9.13 |
| Wang, 2018a | 2015 | Northwestern | Outpatient clinics | CS | Conv | Healthy adults during January 2013 - December 2013 | ELISA | 3284 | 0.03 |
| Wang, 2018a | 2015 | Northwestern | Outpatient clinics | CS | Conv | Healthy adults during January 2014 - June 2014 | ELISA | 2163 | 0.14 |
| Wang, 2019a | 2015 | Northwestern | Outpatient clinics | CS | Conv | 20-25 years ole women | ELISA |  | 6.24 |
| Wang, 2019a | 2016 | Northwestern | Outpatient clinics | CS | Conv | 26-29 years old womne | ELISA |  | 6.00 |
| Wang, 2019a | 2017 | Northwestern | Outpatient clinics | CS | Conv | >=30 years old women | ELISA |  | 6.22 |
| Wang, 2019b | 2011-17 | Eastern | Outpatient clinics | CS | Conv | Pregnant women | ELISA | 2267 | 7.59 |
| Wang, 2019b | 2011-17 | Eastern | Outpatient clinics | CS | Conv | Newborn | ELISA | 2614 | 5.66 |
| Wang, 2022 | 2019 | Central-southern | Outpatient clinics | CS | Conv | Males | ELISA | 8988 | 7.39 |
| Wang, 2022 | 2019 | Central-southern | Outpatient clinics | CS | Conv | Famales | ELISA | 11597 | 14.26 |
| Warnecke, 2020 | 2017-18 | Multilple or unknown | Outpatient clinics | CS | Conv | Women of childbearing age | ELISA | 232 | 7.33 |
| Wei, 2009 | 2007-18 | Central-southern | Outpatient clinics | CS | RS | Observation group: Pregnant women with adverse pregnancy | ELISA | 550 | 13.45 |
| Wei, 2009 | 2007-18 | Central-southern | Outpatient clinics | CS | RS | Control group: Pregnant women without adverse pregnancy | ELISA | 550 | 2.73 |
| Wen, 1998 | － | Southwestern | Outpatient clinics | CS | RS | Pregnant women | ELISA | 94 | 22.34 |
| Wen, 2009 | 2008 | Central-southern | Outpatient clinics | CS | Conv | Pregnant women | ELISA | 2521 | 0.83 |
| Wu, 1999 | 1995-96 | North | Outpatient clinics | CS | RS | Pregnant women | ELISA | 464 | 10.56 |
| Wu, 2003 | 2000-02 | Central-southern | Outpatient clinics | CS | Conv | Pregnant women | ELISA | 495 | 2.42 |
| Wu, 2004 | 2001-03 | Southwestern | Outpatient clinics | CS | Conv | Pregnant women | ELISA | 2308 | 1.04 |
| Wu, 2009 | 2008 | Central-southern | Outpatient clinics | CS | Conv | Pregnant women | ELISA | 558 | 6.45 |
| Wu, 2013a | 2010-12 | Eastern | Outpatient clinics | CS | Conv | Pregnant women of appropriate age | ELISA | 2586 | 0.73 |
| Wu, 2013a | 2010-12 | Eastern | Outpatient clinics | CS | Conv | Older pregnant woman | ELISA | 259 | 0.77 |
| Wu, 2014 | 2012-14 | Central-southern | Outpatient clinics | CS | Conv | pre-pregnant and pregnant woman | ELISA | 1126 | 0.62 |
| Wu, 2016 | 2010-13 | Northwestern | Outpatient clinics | CS | Conv | Childbearing age females | Other | 336 | 1.49 |
| Wu, 2017 | 2010-16 | Eastern | Outpatient clinics | CS | Conv | Healthy adults aged 17-22 years | Other | 335 | 11.94 |
| Wu, 2017 | 2010-16 | Eastern | Outpatient clinics | CS | Conv | Healthy adults aged 23-28 years | Other | 3205 | 8.74 |
| Wu, 2017 | 2010-16 | Eastern | Outpatient clinics | CS | Conv | Healthy adults aged 29-34 years | Other | 1735 | 13.26 |
| Wu, 2017 | 2010-16 | Eastern | Outpatient clinics | CS | Conv | Healthy adults aged >35 years | Other | 935 | 16.04 |
| Wu, 2020 | 2017-19 | Southwestern | Outpatient clinics | CS | Conv | Childbearing age females | IF | 13774 | 20.29 |
| Wu, 2020 | 2017-19 | Southwestern | Outpatient clinics | CS | Conv | Minors | IF | 263 | 4.94 |
| Wu, 2020 | 2017-19 | Southwestern | Outpatient clinics | CS | Conv | Adult | IF | 13511 | 20.59 |
| Xia, 2018 | 2015-17 | Eastern | Outpatient clinics | CS | Conv | Pregnant women | ELISA | 1000 | 1.30 |
| Xiao, 2006 | 2003-05 | Central-southern | Outpatient clinics | CS | Conv | Pregnant women | ELISA | 2364 | 2.37 |
| Xie, 2009 | 2008-09 | Central-southern | Outpatient clinics | CS | Conv | Pregnant women | ELISA | 250 | 6.00 |
| Xie, 2015 | 2011-13 | Northwestern | Outpatient clinics | CS | Conv | Healthy adults in 2012 | ELISA | 4425 | 3.50 |
| Xie, 2015 | 2011-13 | Northwestern | Outpatient clinics | CS | Conv | Healthy adults in 2013 | ELISA | 4913 | 3.38 |
| Xie, 2023a | 2020-22 | Eastern | Outpatient clinics | CS | Conv | Healthy children aged 29d-6 years | Other | 5567 | 2.96 |
| Xie, 2023a | 2020-22 | Eastern | Outpatient clinics | CS | Conv | Healthy adolescent aged 7-17 years | Other | 1281 | 1.87 |
| Xie, 2023a | 2020-22 | Eastern | Outpatient clinics | CS | Conv | Healthy adults aged 18-29 years | Other | 20696 | 5.79 |
| Xie, 2023a | 2020-22 | Eastern | Outpatient clinics | CS | Conv | Healthy adults aged 30-39 years | Other | 16198 | 13.25 |
| Xie, 2023a | 2020-22 | Eastern | Outpatient clinics | CS | Conv | Healthy adults aged 40-49 years | Other | 5674 | 27.16 |
| Xie, 2023a | 2020-22 | Eastern | Outpatient clinics | CS | Conv | Healthy adults aged ≥50 years | Other | 8798 | 19.85 |
| Xie, 2023a | 2020-22 | Eastern | Outpatient clinics | CS | Conv | Healthy adults in 2020 | Other | 23888 | 10.68 |
| Xie, 2023a | 2020-22 | Eastern | Outpatient clinics | CS | Conv | Healthy adults in 2021 | Other | 26176 | 11.50 |
| Xie, 2023a | 2020-22 | Eastern | Outpatient clinics | CS | Conv | Healthy adults in 2022 | Other | 14049 | 10.95 |
| Xie, 2023a | 2020-22 | Eastern | Outpatient clinics | CS | Conv | Male | Other | 24049 | 12.47 |
| Xie, 2023a | 2020-22 | Eastern | Outpatient clinics | CS | Conv | Female | Other | 40064 | 10.24 |
| Xie, 2023b | 2016-20 | North | Outpatient clinics | CS | Conv | Pregnant women aged <35 years | ELISA | 3051 | 5.47 |
| Xie, 2023b | 2016-20 | North | Outpatient clinics | CS | Conv | Pregnant women aged ≥35 years | ELISA | 963 | 8.52 |
| Xiong, 2014 | 2009-12 | Eastern | Outpatient clinics | CS | Conv | Pregnant women | ELISA | 1593 | 9.73 |
| Xu, 2010 | 2008 | Southwestern | Outpatient clinics | CS | RS | Famale high school students | ELISA | 1520 | 60.00 |
| Yan, 2014b | 2010-11 | Northwestern | Outpatient clinics | CS | Conv | Early pregnant women | Other | 102 | 3.92 |
| Yang, 2000 | － | North | Outpatient clinics | CS | Conv | Pregnant women | ELISA | 282 | 12.77 |
| Yang, 2003b | 2000-02 | Southwestern | Community | CS | Conv | Pregnant women | ELISA | 3286 | 0.55 |
| Yang, 2005 | － | Central-southern | Community | CS | Conv | Marriage status: Married non-pregnant women of childbearing age | ELISA | 1482 | 39.27 |
| Yang, 2005 | － | Central-southern | Community | CS | Conv | Early pregnant women | ELISA | 518 | 42.86 |
| Yang, 2007 | 2004-06 | Northwestern | Outpatient clinics | CS | Conv | Pregnant women | Other | 1331 | 1.13 |
| Yang, 2008 | － | Central-southern | Outpatient clinics | CS | Conv | Healthy adults | ELISA | 580 | 50.34 |
| Yang, 2009 | 2007-08 | Northwestern | Outpatient clinics | CS | Conv | Early pregnant women | ELISA | 1980 | 0.91 |
| Yang, 2012b | － | Central-southern | Community | CS | Conv | Marriage status: Married non-pregnant women of childbearing age | ELISA | 1482 | 39.27 |
| Yang, 2012b | － | Central-southern | Community | CS | Conv | Early pregnant women | ELISA | 518 | 42.86 |
| Yang, 2013c | 2012 | Eastern | Outpatient clinics | CS | Conv | Pregnant women | ELISA | 4761 | 0.63 |
| Yang, 2016 | 2014-16 | Eastern | Outpatient clinics | CS | Conv | Pregnant women | Other | 4896 | 3.74 |
| Yao, 2023a | 2019-21 | Central-southern | -Outpatient clinics | CS | Conv | Healthy adolescent aged 15-19 years | ELISA | 7 | 28.57 |
| Yao, 2023a | 2019-21 | Central-southern | -Outpatient clinics | CS | Conv | Healthy adults aged 20-34 years | ELISA | 2453 | 1.63 |
| Yao, 2023a | 2019-21 | Central-southern | -Outpatient clinics | CS | Conv | Healthy adults aged 35-49 years | ELISA | 941 | 0.43 |
| Yao, 2023a | 2019-21 | Central-southern | -Outpatient clinics | CS | Conv | Healthy adults in 2019 | ELISA | 1589 | 1.32 |
| Yao, 2023a | 2019-21 | Central-southern | -Outpatient clinics | CS | Conv | Healthy adults in 2020 | ELISA | 1327 | 1.36 |
| Yao, 2023a | 2019-21 | Central-southern | -Outpatient clinics | CS | Conv | Healthy adults in 2021 | ELISA | 485 | 1.44 |
| Yao, 2023b | 2019-21 | Central-southern | Other | CS | Conv | Healthy children aged 0-18 years | ELISA | 3390 | 1.77 |
| Ye, 2005 | 2003-04 | Central-southern | Outpatient clinics | CS | Conv | Healthy adults | ELISA | 1008 | 45.44 |
| Ye, 2013 | 2010-11 | Eastern | Outpatient clinics | CS | Conv | Healthy adults | ELISA | 1567 | 0.13 |
| Yin, 2022 | 2018-19 | North | Outpatient clinics | CS | Conv | Healthy adults aged 20-29 years | Other | 510 | 7.84 |
| Yin, 2022 | 2018-19 | North | Outpatient clinics | CS | Conv | Healthy adults aged 30-39 years | Other | 468 | 7.91 |
| Yin, 2022 | 2018-19 | North | Outpatient clinics | CS | Conv | Healthy adults aged ≥40 years | Other | 63 | 7.94 |
| You, 2017 | 2015-16 | Eastern | Outpatient clinics | CS | Conv | Healthy adults | ELISA | 7866 | 1.67 |
| Yu, 2005 | 1999-04 | Eastern | Outpatient clinics | CS | Conv | Healthy adults | ELISA | 7832 | 1.38 |
| Yu, 2019 | 2017-18 | Eastern | Outpatient clinics | CS | Conv | Healthy adults | ELISA | 2962 | 0.88 |
| Yuan, 2004 | 2000-03 | Eastern | Outpatient clinics | CS | Conv | Healthy adults | ELISA | 18424 | 1.48 |
| Yuan, 2021 | 2018-19 | Northwestern | Outpatient clinics | CS | Conv | Healthy adults | ELISA | 1316 | 10.64 |
| Yue, 2005 | 2000-02 | Southwestern | Outpatient clinics | CS | Conv | Healthy adults | ELISA | 1820 | 1.26 |
| Zhang, 2000 | 2009 | Central-southern | Outpatient clinics | CS | Conv | Healthy adults | ELISA | 1238 | 7.84 |
| Zhang, 2002b | 1999 | Eastern | Inpatient | CS | Conv | Healthy adults | ELISA | 1062 | 59.23 |
| Zhang, 2003 | 1999-02 | Eastern | Outpatient clinics | CS | Conv | Healthy adults in 1999 | ELISA | 956 | 0.52 |
| Zhang, 2003 | 1999-02 | Eastern | Outpatient clinics | CS | Conv | Healthy adults in 2000 | ELISA | 842 | 0.48 |
| Zhang, 2003 | 1999-02 | Eastern | Outpatient clinics | CS | Conv | Healthy adults in 2001 | ELISA | 1018 | 0.49 |
| Zhang, 2003 | 1999-02 | Eastern | Outpatient clinics | CS | Conv | Healthy adults in 2002 | ELISA | 774 | 0.65 |
| Zhang, 2007c | 2003-06 | Central-southern | Outpatient clinics | CS | Conv | Healthy adults | ELISA | 921 | 3.58 |
| Zhang, 2007d | 2007 | Central-southern | Outpatient clinics | CS | Conv | Healthy adults | ELISA | 2849 | 29.48 |
| Zhang, 2007e | 2003-05 | Northwestern | Outpatient clinics | CS | Conv | Healthy adults in 2003 | ELISA | 968 | 3.62 |
| Zhang, 2007e | 2003-05 | Northwestern | Outpatient clinics | CS | Conv | Healthy adults in 2004 | ELISA | 1165 | 6.18 |
| Zhang, 2007e | 2003-05 | Northwestern | Outpatient clinics | CS | Conv | Healthy adults in 2005 | ELISA | 1132 | 7.16 |
| Zhang, 2007f | － | North | Community | CS | MSCS | Healthy adults | ELISA | 1797 | 25.93 |
| Zhang, 2010b | 2008-10 | Multilple or unknown | Community | CS | Conv | Healthy adults | ELISA | 3164 | 13.65 |
| Zhang, 2011a | 2008-10 | Northwestern | Outpatient clinics | CS | Conv | Healthy adults | ELISA | 700 | 0.71 |
| Zhang, 2011b | 2007-09 | North | Outpatient clinics | CS | Conv | Healthy adults | ELISA | 5609 | 1.98 |
| Zhang, 2012a | 2011 | North | Outpatient clinics | CS | Conv | pregnant women with preeclampsia | ELISA | 52 | 3.85 |
| Zhang, 2012a | 2011 | North | Outpatient clinics | CS | Conv | healthy pregnant women | ELISA | 35 | 5.71 |
| Zhang, 2012b | 2009-10 | Northwestern | Outpatient clinics | CS | Conv | Pregnancy | ELISA | 689 | 0.73 |
| Zhang, 2012b | 2009-10 | Northwestern | Outpatient clinics | CS | Conv | Newborn | ELISA | 564 | 0.35 |
| Zhang, 2012c | 2008-11 | Southwestern | Community | CS | Conv | Healthy adults | Other | 2096 | 2.58 |
| Zhang, 2013b | － | Northwestern | -Outpatient clinics | CS | Conv | Healthy adults | ELISA | 419 | 0.72 |
| Zhang, 2014a | 2011 | Eastern | Community | CS | RS | 18-25 years old women | ELISA | 262 | 9.92 |
| Zhang, 2014a | 2011 | Eastern | Community | CS | RS | 26-35 years old women | ELISA | 175 | 15.43 |
| Zhang, 2014a | 2011 | Eastern | Community | CS | RS | >35 years old women | ELISA | 163 | 23.93 |
| Zhang, 2014b | 2012 | Eastern | Outpatient clinics | CS | Conv | Healthy adults | ELISA | 3635 | 0.25 |
| Zhang, 2015 | 2012 | Eastern | Other | CS | RS | HIV, HCV, Syphilis negative aged 16-29 years | ELISA | 248 | 10.89 |
| Zhang, 2015 | 2012 | Eastern | Other | CS | RS | HIV, HCV, Syphilis negative aged 39-39 years | ELISA | 287 | 16.72 |
| Zhang, 2015 | 2012 | Eastern | Other | CS | RS | HIV, HCV, Syphilis negative aged 40-49 years | ELISA | 209 | 21.05 |
| Zhang, 2015 | 2012 | Eastern | Other | CS | RS | HIV, HCV, Syphilis negative aged 50-69 years | ELISA | 130 | 26.92 |
| Zhang, 2015 | 2012 | Eastern | Other | CS | RS | One of HIV, HCV, Syphilis positive aged 16-29 years | ELISA | 32 | 25.00 |
| Zhang, 2015 | 2012 | Eastern | Other | CS | RS | One of HIV, HCV, Syphilis positive aged 39-39 years | ELISA | 41 | 29.27 |
| Zhang, 2015 | 2012 | Eastern | Other | CS | RS | One of HIV, HCV, Syphilis positive aged 40-49 years | ELISA | 62 | 29.03 |
| Zhang, 2015 | 2012 | Eastern | Other | CS | RS | One of HIV, HCV, Syphilis positive aged 50-69 years | ELISA | 32 | 43.75 |
| Zhang, 2017 | 2014-15 | Eastern | Outpatient clinics | CS | Conv | Healthy adults aged <35 years | ELISA | 4429 | 6.86 |
| Zhang, 2017 | 2014-15 | Eastern | Outpatient clinics | CS | Conv | Healthy adults aged ≥35 years | ELISA | 552 | 7.25 |
| Zhang, 2023 | 2020-22 | Southwestern | Outpatient clinics | CS | Conv | 20-35 years child-bearing period women | Other | 945 | 11.96 |
| Zhang, 2023 | 2020-22 | Southwestern | Outpatient clinics | CS | Conv | Child-bearing period women aged 35-42 years | Other | 202 | 13.86 |
| Zhang, 2023 | 2020-22 | Southwestern | Outpatient clinics | CS | Conv | 6 months-5 years Infants and children | Other | 37 | 10.81 |
| Zhang, 2023a | 2020-22 | Southwestern | Outpatient clinics | CS | Conv | 120-42 years old women of child-bearing age and children | ELISA | 1311 | 13.81 |
| Zhao, 2004 | 2001-02 | North | Outpatient clinics | CS | Conv | Healthy adults | ELISA | 1966 | 0.61 |
| Zhao, 2017 | 2013-14 | Eastern | Outpatient clinics | CS | Conv | Male | ELISA | 1058 | 3.78 |
| Zhao, 2017 | 2013-14 | Eastern | Outpatient clinics | CS | Conv | Female | ELISA | 1058 | 4.91 |
| Zhao, 2020 | 2017-19 | Northwestern | Outpatient clinics | CS | Conv | Healthy adults aged 18-25 years | Other | 4582 | 0.04 |
| Zhao, 2020 | 2017-19 | Northwestern | Outpatient clinics | CS | Conv | Healthy adults aged 26-35 years | Other | 8160 | 0.22 |
| Zhao, 2020 | 2017-19 | Northwestern | Outpatient clinics | CS | Conv | Healthy adults aged 36-45 years | Other | 1352 | 0.07 |
| Zheng, 2021 | 2018-20 | Eastern | Outpatient clinics | CS | Conv | Healthy adults aged <35 years | ELISA | 15257 | 9.37 |
| Zheng, 2021 | 2018-20 | Eastern | Outpatient clinics | CS | Conv | Healthy adults aged ≥35 years | ELISA | 2727 | 19.36 |
| Zhong, 2000 | － | Central-southern | Outpatient clinics | CS | Conv | Healthy adults | ELISA | 282 | 18.44 |
| Zhong, 2008a | － | Eastern | Community | CS | Conv | Healthy adults aged <20 years | ELISA | 14 | 7.14 |
| Zhong, 2008a | － | Eastern | Community | CS | Conv | Healthy adults aged 20-29 years | ELISA | 90 | 3.33 |
| Zhong, 2008a | － | Eastern | Community | CS | Conv | Healthy adults aged 30-39 years | ELISA | 205 | 8.78 |
| Zhong, 2008a | － | Eastern | Community | CS | Conv | Healthy adults aged ≥40 years | ELISA | 89 | 10.11 |
| Zhou, 2000 | 1997-98 | Northwestern | Outpatient clinics | CS | Conv | Pregnancy women overall | ELISA | 1529 | 1.57 |
| Zhou, 2007a | 2005-06 | Central-southern | Outpatient clinics | CS | Conv | Healthy adults | ELISA | 991 | 5.65 |
| Zhou, 2011a | 2009-10 | Eastern | Outpatient clinics | CS | Conv | Pregnancy women overall | ELISA | 560 | 7.32 |
| Zhou, 2013b | 2009-11 | Eastern | Outpatient clinics | CS | Conv | Healthy adults in 2009 | ELISA | 4369 | 1.97 |
| Zhou, 2013b | 2009-11 | Eastern | Outpatient clinics | CS | Conv | Healthy adults in 2010 | ELISA | 5233 | 1.97 |
| Zhou, 2013b | 2009-11 | Eastern | Outpatient clinics | CS | Conv | Healthy adults in 2011 | ELISA | 5260 | 1.94 |
| Zhou, 2018a | 2017 | Central-southern | Outpatient clinics | CS | Conv | Pregnancy women overall | IF | 1745 | 9.17 |
| Zhou, 2018b | 2015-17 | Southwestern | Outpatient clinics | CS | Conv | Healthy adults | ELISA | 2393 | 9.11 |
| Zhou, 2019 | 2015-17 | Eastern | Outpatient clinics | CS | Conv | Healthy adults aged 17-25 years | ELISA | 4897 | 1.47 |
| Zhou, 2019 | 2015-17 | Eastern | Outpatient clinics | CS | Conv | Healthy adults aged 26-35 years | ELISA | 9632 | 1.21 |
| Zhou, 2019 | 2015-17 | Eastern | Outpatient clinics | CS | Conv | Healthy adults aged 36-47 years | ELISA | 3983 | 2.96 |
| Zhou, 2020 | 2015-18 | Southwestern | Outpatient clinics | CS | Conv | Healthy adults | ELISA | 2310 | 1.00 |
| Zhu, 2007 | 2005-06 | Eastern | Outpatient clinics | CS | Conv | Healthy adults | ELISA | 2686 | 0.00 |
| Zhu, 2018a | 2016-17 | Central-southern | Outpatient clinics | CS | Conv | Male | ELISA | 24873 | 2.18 |
| Zhu, 2018a | 2016-17 | Central-southern | Outpatient clinics | CS | Conv | Female | ELISA | 28838 | 3.36 |
| Zuo, 2016 | 2014-15 | Central-southern | Outpatient clinics | CS | Conv | Healthy adults | ELISA | 3980 | 17.99 |

^a^ The reported study design is the original study design (case control, cross sectional, cohort, or randomized controlled trial). The included seroprevalence measures are those for the baseline measures at the beginning of the study.

Abbreviations: CC = Case-control, Conv = Convenience, CS = Cross-sectional, CRS = Cluster random sampling,MSCS = Multiple stage cluster sampling, RS = Random sampling, TLS=Time-location sampling, SS = Snowball sampling, Ig M = Immunoglobulin M, Ig G =Immunoglobulin G, AMI = Acute myocardial infarction, ELISA = Enzyme-linked immunosorbent type-specific assay, HIV = Human immunodeficiency virus, HSV-2 = Herpes simplex virus type 2, IF= Immunofluorescent, WB = Western blot, HPV= Human papilloma virus, HCV= Hepatitis C virus..

# **Supplementary Table S4.** Studies reporting HSV-2 seroprevalence among intermediate-risk populations in China.

| **Author, year** | **Year(s) of data collection** | **Region** | **Study site** | **Original study design**a | **Sampling method** | **Population** | **HSV-2 serological assay** | **Sample size** | **HSV-2 seroprevalence (%)** |
| --- | --- | --- | --- | --- | --- | --- | --- | --- | --- |
| Chen, 2006a | 2000 | Middle | Community | CS | Conv | 20-24 years old truck driveers | ELISA | 54.00 | 5.56 |
| Chen, 2006a | 2000 | Middle | Community | CS | Conv | 25-29 years old truck drivers | ELISA | 143.00 | 4.20 |
| Chen, 2006a | 2000 | Middle | Community | CS | Conv | 30-34 years old truck drivers | ELISA | 154.00 | 3.90 |
| Chen, 2006a | 2000 | Middle | Community | CS | Conv | 35-39 years old truck drivers | ELISA | 111.00 | 4.50 |
| Chen, 2006a | 2000 | Middle | Community | CS | Conv | >40 years old trcuk drivers | ELISA | 83.00 | 4.82 |
| Gao, 2006 | 2005-06 | Western | Community | CS | Conv | 20–35 years old Miners | ELISA | 352 | 15.06 |
| Gao, 2006 | 2005-06 | Western | Community | CS | Conv | <20 or ≥35 years old Miners | ELISA | 152 | 9.21 |
| Reilly, 2012 | 2010 | Western | Community | CS | CRS | Male clients of FSWs | ELISA | 306.00 | 26.47 |
| Song, 2006 | — | Western | Community | CS | RS | Male Taxi Drivers | ELISA | 139.00 | 37.41 |
| Wei, 2004 | 1999 | Middle | Community | CS | Conv | Non-sex Hospitality girls | ELISA | 84.00 | 11.90 |
| Wu, 2007 | — | Eastern | Community | CS | Conv | Female market vendors | ELISA | 2378.00 | 8.20 |
| Wu, 2007 | — | Eastern | Community | CS | Conv | Male market vendors | ELISA | 2132.00 | 4.64 |
| Xu, 2008 | 2006 | Western | Community | CS | Conv | Miner non-clients of FSWs | ELISA | 1424.00 | 8.36 |
| Xu, 2008 | 2006 | Western | Community | CS | Conv | Miner clients of FSWs | ELISA | 336.00 | 14.88 |
| Yang, 2003a | 2000-01 | Middle | Community | CS | Conv | Bar and hotel workers | ELISA | 165.00 | 21.82 |
| Zeng, 2010 | — | Eastern | Community | CS | RS | Taxi drivers | ELISA | 178 | 40.45 |
| Zhang, 2007a | 2006 | Western | Community | CS | Conv | Clients of FSWs/MSWs | ELISA | 336.00 | 14.88 |
| Zhang, 2007b | 2006 | Western | Community | CS | Conv | ≤25 years old Miners | ELISA | 656.00 | 5.64 |
| Zhang, 2007b | 2006 | Western | Community | CS | Conv | >25 years old Miners | ELISA | 1104.00 | 11.96 |
| Zhang, 2010a | 2008-09 | Western | Community | CS | Conv | >25 years old clients of FSWs/MSWs in first survey | ELISA | 221.00 | 36.20 |
| Zhang, 2010a | 2008-09 | Western | Community | CS | Conv | ≤25 years old clients of FSWs/MSWs in first survey | ELISA | 88.00 | 6.82 |
| Zhang, 2010a | 2008-09 | Western | Community | CS | Conv | >25 years old clients of FSWs/MSWs in second survey | ELISA | 211.00 | 28.91 |
| Zhang, 2010a | 2008-09 | Western | Community | CS | Conv | ≤25 years old clients of FSWs/MSWs in second survey | ELISA | 102.00 | 9.80 |
| Zhang, 2010a | 2008-09 | Western | Community | CS | Conv | >25 years old clients of FSWs/MSWs in third survey | ELISA | 208.00 | 38.46 |
| Zhang, 2010a | 2008-09 | Western | Community | CS | Conv | ≤25 years old clients of FSWs/MSWs in third survey | ELISA | 126.00 | 9.52 |
| Zhou, 2012 | 2010 | Eastern | Community | CS | Conv | Bar and hotel workers | ELISA | 391 | 34.27 |
| Zhu, 2020 | 2015-16 | Western | Community | CS | SS | Clients of FSWs/MSWs | ELISA | 776 | 16.49 |
| Zhu, 2019 | 2014-15 | Western | Community | CS | SS | Male clients of FSWs in Hekou county | ELISA | 202.00 | 20.79 |
| Zhu, 2019 | 2014-15 | Western | Community | CS | SS | Male clients of FSWs t in other regions of China | ELISA | 519.00 | 15.61 |

^a^ The reported study design is the original study design (case control, cross sectional, cohort, or randomized controlled trial). The included seroprevalence measures are those for the baseline measures at the beginning of the study.

Abbreviations: CS = Cross-sectional, Conv = Convenience, SS = Snowball sampling, RS = Random samplin, CRS=Cluster random sampling, FSWs = Female sex workers, MSWS=Male sex workers, ELISA = Enzyme-linked immunosorbent type-specific assay, HIV = Human immunodeficiency virus, HSV-2 = Herpes simplex virus type 2.

# **Supplementary Table S5.** Studies reporting HSV-2 seroprevalence among key populations in China.

| **Author, year** | **Year(s) of data collection** | **Region** | **Study site** | **Original study design^a^** | **Sampling method** | **Population** | **HSV-WB serological assay** | **Sample size** | **HSV-2 seroprevalence (%)** |
| --- | --- | --- | --- | --- | --- | --- | --- | --- | --- |
| Cao, 2006 | 2003 | Eastern | Community | CS | Conv | MSMs | ELISA | 90 | 7.78 |
| Chang, 2010 | － | Central-southern | Outpatient clinics | CS | Conv | MMT 1 drug takers in Guangxi Province | ELISA | 280 | 58.93 |
| Chang, 2010 | － | Southwestern | Outpatient clinics | CS | Conv | MMT 1 drug takers in Guizhou Province | ELISA | 153 | 54.25 |
| Chen, 2005 | 1999-20 | Southwestern | Outpatient clinics | CS | Conv | 15-19 years old FSWs | ELISA | 70 | 84.29 |
| Chen, 2005 | 1999-20 | Southwestern | Outpatient clinics | CS | Conv | 20-24 years old FSWs | ELISA | 204 | 86.76 |
| Chen, 2005 | 1999-20 | Southwestern | Outpatient clinics | CS | Conv | 25-29 years old FSWs | ELISA | 144 | 79.86 |
| Chen, 2005 | 1999-20 | Southwestern | Outpatient clinics | CS | Conv | 30-34 years old FSWs | ELISA | 62 | 85.48 |
| Chen, 2005 | 1999-20 | Southwestern | Outpatient clinics | CS | Conv | 35-39 years old FSWs | ELISA | 25 | 88.00 |
| Chen, 2013a | 2009 | Central-southern | Community | CS | Conv | >26 years old FSWs | ELISA | 1045 | 65.17 |
| Chen, 2013a | 2009 | Central-southern | Community | CS | Conv | <26 years old FSWs | ELISA | 1408 | 47.30 |
| Chen, 2015 | 2009 | Central-southern | Community | CS | SS | Drug users MSM | ELISA | 177 | 16.95 |
| Chen, 2015 | 2009 | Central-southern | Community | CS | SS | Drug non-users MSM | ELISA | 649 | 12.79 |
| Dai, 2009 | 2006 | Central-southern | Other | CS | Conv | FSWs | ELISA | 202 | 47.52 |
| Ding, 2009 | 2008 | Southwestern | Outpatient clinics | CS | SS | MSM | ELISA | 945 | 2.65 |
| Ding, 2010 | 2008 | Southwestern | Outpatient clinics | CS | SS | MSM | ELISA | 743 | 3.36 |
| Ding, 2017 | 2014 | Eastern | Other | CS | Conv | MSM | ELISA | 243 | 9.47 |
| Duan, 2018 | － | Eastern | Other | CS | Conv | ≤19 years old female drug users | ELISA | 41 | 56.10 |
| Duan, 2018 | － | Eastern | Other | CS | Conv | 20–30 years old female drug users | ELISA | 288 | 74.31 |
| Duan, 2018 | － | Eastern | Other | CS | Conv | ≥30 years old female drug users | ELISA | 121 | 71.90 |
| Fan, 2010 | 2009 | North | Other | CS | Conv | 18–30 years old drug abusers | ELISA | 62 | 61.29 |
| Fan, 2010 | 2009 | North | Other | CS | Conv | 30–40 years old drug abusers | ELISA | 40 | 57.50 |
| Fan, 2010 | 2009 | North | Other | CS | Conv | ≥40 years old drug abusers | ELISA | 24 | 62.50 |
| Fan, 2017 | 2015-16 | Eastern | Outpatient clinics | CS | Conv | MSM | ELISA | 127 | 3.15 |
| Feng, 2010 | 2007 | Southwestern | Community | CS | SS | MSM | ELISA | 538 | 24.72 |
| Gao, 2006 | 2005 | Southwestern | Community | CS | Conv | 16–20 years old FSWs | ELISA | 26 | 42.31 |
| Gao, 2006 | 2005 | Southwestern | Community | CS | Conv | 20–30 years old FSWs | ELISA | 137 | 64.23 |
| Gao, 2006 | 2005 | Southwestern | Community | CS | Conv | ≥30 years old FSWs | ELISA | 74 | 72.97 |
| Gao, 2012 | 2009 | North | Outpatient clinics | CS | SS | ≤25 years old MSM | ELISA | 411 | 2.19 |
| Gao, 2012 | 2009 | North | Outpatient clinics | CS | SS | >25 years old MSM | ELISA | 551 | 7.62 |
| Guo, 2014 | － | Northwestern | Community | CS | Conv | FSWs | ELISA | 513 | 29.43 |
| Guo, 2015 | 2014 | Northwestern | Other | CS | Conv | ≤30 years old FSWs | ELISA | 264 | 23.48 |
| Guo, 2015 | 2014 | Northwestern | Other | CS | Conv | >30 years old FSWs | ELISA | 266 | 38.72 |
| Han, 2015 | 2013 | Eastern | Online survey | CS | SS | ≤20 years old MSM | ELISA | 34 | 0.00 |
| Han, 2015 | 2013 | Eastern | Online survey | CS | SS | 21-40 years old MSM | ELISA | 306 | 8.82 |
| Han, 2015 | 2013 | Eastern | Online survey | CS | SS | >40 years old MSM | ELISA | 60 | 15.00 |
| Han, 2016 | 2012 | Multiple or unknown | Community | CS | Conv | FSWs | ELISA | 1487 | 27.84 |
| Han, 2018 | 2015-17 | Eastern | Online survey | CS | SS | MSM | ELISA | 1300 | 5.62 |
| Hu, 2017 | 2009-14 | Northeastern | Outpatient clinics | CS | Conv | <24 years old HIV-positive MSM | ELISA | 105 | 23.81 |
| Hu, 2017 | 2009-14 | Northeastern | Outpatient clinics | CS | Conv | 25-50 years old HIV-positive MSM | ELISA | 365 | 49.59 |
| Hu, 2017 | 2009-14 | Northeastern | Outpatient clinics | CS | Conv | >50 years old HIV-positive MSM | ELISA | 75 | 78.67 |
| Jiang, 2006 | 2003 | Eastern | Community | CS | Conv | MSM | ELISA | 90 | 7.78 |
| Li, 2006b | 2005 | North | Outpatient clinics | CS | Conv | MSM | ELISA | 85 | 8.24 |
| Li, 2014a | 2013 | Eastern | Community | CS | Conv | FSWs | ELISA | 460 | 43.04 |
| Li, 2016d | 2009-10 | North | Community | Cohort | Conv | MSM | ELISA | 962 | 5.30 |
| Liang, 2005a | 2002-04 | Central-southern | Outpatient clinics | CS | Conv | MSM and bisexuality persons | ELISA | 146 | 4.79 |
| Liu, 2004b | 2002-03 | Eastern | Other | CS | Conv | ≤25 years old FSWs | ELISA | 212 | 33.49 |
| Liu, 2004b | 2002-03 | Eastern | Other | CS | Conv | >25 years old FSWs | ELISA | 112 | 47.32 |
| Liu, 2012 | 2009 | Central-southern | Community | CS | CRS | Money boys | ELISA | 418 | 11.00 |
| Liu, 2017b | 2015 | Eastern | Outpatient clinics | CS | Conv | 18-25 years old MSM | ELISA | 266 | 3.38 |
| Liu, 2017b | 2015 | Eastern | Outpatient clinics | CS | Conv | 26-35 years old MSM | ELISA | 348 | 5.75 |
| Liu, 2017b | 2015 | Eastern | Outpatient clinics | CS | Conv | 36-74 years old MSM | ELISA | 118 | 10.17 |
| Liu, 2017d | 2013-14 | Eastern | Community | CS | Conv | MSM | ELISA | 486 | 22.43 |
| Liu, 2023 | 2020-21 | Multiple or unknown | Online survey | CS | Conv | MSM/MSWs | ELISA | 612 | 27.12 |
| Lo, 1999 | 1995 | SARs and Taiwan | Outpatient clinics | CS | Conv | FSW STD clinic attendees | WB | 40 | 77.50 |
| Lu, 2019 | 2015-18 | Central-southern | Outpatient clinics | CS | Conv | <30 years old MSM | ELISA | 1142 | 1.84 |
| Lu, 2019 | 2015-18 | Central-southern | Outpatient clinics | CS | Conv | ≥30 years old MSM | ELISA | 462 | 2.81 |
| Luo, 2008 | 2006 | Southwestern | Community | CS | Conv | FSWs | ELISA | 216 | 38.43 |
| Luo, 2015a | 2009-12 | North | Community | CS | Conv | FSWs who were lost to follow up | ELISA | 1096 | 60.40 |
| Luo, 2015a | 2009-12 | North | Community | CS | Conv | FSWs who had at least 1 follow up | ELISA | 792 | 66.54 |
| Luo, 2015b | 2013 | Eastern | Community | CS | Conv | FSWs | ELISA | 837 | 68.10 |
| Ma, 2016 | 2014-15 | Central-southern | Community | CS | SS | MSM | ELISA | 467 | 11.35 |
| Mao, 2018 | 2012-13 | Multiple or unknown | Community | CS | Conv | Younger MSM | ELISA | 1289 | 7.60 |
| Mao, 2018 | 2012-13 | Multiple or unknown | Community | CS | Conv | Older MSM | ELISA | 3131 | 14.50 |
| Mao, 2021a | 2012-18 | Central-southern | Community | CS | TLS | ≤24 years old MSM | ELISA | 322 | 6.52 |
| Mao, 2021a | 2012-18 | Central-southern | Community | CS | TLS | 25–34 years old MSM | ELISA | 867 | 15.11 |
| Mao, 2021a | 2012-18 | Central-southern | Community | CS | TLS | 35–44 years old MSM | ELISA | 374 | 20.05 |
| Mao, 2021a | 2012-18 | Central-southern | Community | CS | TLS | ≧45 years old MSM | ELISA | 132 | 31.06 |
| Mao, 2021b | 2018 | Central-southern | Community | CS | TLS | ＜25 years old MSM | ELISA | 62 | 6.45 |
| Mao, 2021b | 2018 | Central-southern | Community | CS | TLS | 25-35 years old MSM | ELISA | 177 | 12.99 |
| Mao, 2021b | 2018 | Central-southern | Community | CS | TLS | 35-45 years old MSM | ELISA | 68 | 14.71 |
| Mao, 2021b | 2018 | Central-southern | Community | CS | TLS | ≥45 years old MSM | ELISA | 37 | 29.73 |
| Min, 2013 | 2012 | Southwestern | Community | CS | Conv | MSM | ELISA | 458 | 15.50 |
| Ngo, 2008 | 2004 | Southwestern | Other | CS | Conv | <19 years old CSW | ELISA | 58 | 22.41 |
| Ngo, 2008 | 2004 | Southwestern | Other | CS | Conv | >35 years old CSW | ELISA | 98 | 20.41 |
| Ngo, 2008 | 2004 | Southwestern | Other | CS | Conv | 20-24 years old CSW | ELISA | 154 | 47.40 |
| Ngo, 2008 | 2004 | Southwestern | Other | CS | Conv | 25-29 years old CSW | ELISA | 111 | 30.63 |
| Ngo, 2008 | 2004 | Southwestern | Other | CS | Conv | 30-34 years old CSW | ELISA | 78 | 32.05 |
| Peng, 2020 | 2019 | Central-southern | Community | Cohort | RDS | MSM | ELISA | 462 | 3.03 |
| Qi, 2005 | － | Multiple or unknown | Community | CS | Conv | MSM | ELISA | 82 | 6.10 |
| Shen, 2009 | － | Eastern | Community | CS | Conv | 16-19 years old FSWs | ELISA | 54 | 7.41 |
| Shen, 2009 | － | Eastern | Community | CS | Conv | 20-29 years old FSWs | ELISA | 261 | 4.60 |
| Shen, 2009 | － | Eastern | Community | CS | Conv | ≥30 years old FSWs | ELISA | 137 | 5.11 |
| Tang, 2008 | 2005-06 | Central-southern | Community | CS | Conv | FSWs | ELISA | 200 | 47.52 |
| Wang, 2005 | 2004 | Eastern | Community | CS | TLS | FSWs | ELISA | 456 | 32.46 |
| Wang, 2007 | 2006 | Southwestern | Community | CS | TLS | 16-20 years old FSWs | ELISA | 225 | 60.44 |
| Wang, 2007 | 2006 | Southwestern | Community | CS | TLS | 21-25 years old FSWs | ELISA | 255 | 69.80 |
| Wang, 2007 | 2006 | Southwestern | Community | CS | TLS | 26-52 years old FSWs | ELISA | 257 | 73.15 |
| Wang, 2008c | 2006 | Southwestern | Community | CS | TLS | FSWs collected in 2006.3-2006.4 | ELISA | 737 | 68.11 |
| Wang, 2008c | 2006 | Southwestern | Community | CS | TLS | FSWs collected in 20069-2006.10 | ELISA | 747 | 68.54 |
| Wang, 2008d | 2008 | Eastern | Community | CS | Conv | FSWs | ELISA | 338 | 31.07 |
| Wang, 2012c | 2009 | Multiple or unknown | Community | CS | SS | MSM | ELISA | 3227 | 10.66 |
| Wang, 2012e | 2006-09 | Southwestern | Community | CS | Conv | FSWs Sera collected in 2006 (first survey) | ELISA | 741 | 67.34 |
| Wang, 2012e | 2006-09 | Southwestern | Community | CS | Conv | FSWs Sera collected in 2007 (third survey) | ELISA | 705 | 70.78 |
| Wang, 2012e | 2006-09 | Southwestern | Community | CS | Conv | FSWs Sera collected in 2008 (fifth survey) | ELISA | 587 | 68.31 |
| Wang, 2012e | 2006-09 | Southwestern | Community | CS | Conv | FSWs Sera collected in 2009 (seventh survey) | ELISA | 548 | 70.62 |
| Wang, 2012e | 2006-09 | Southwestern | Community | CS | Conv | FSWs Sera collected in 2006 (second survey) | ELISA | 407 | 67.81 |
| Wang, 2012e | 2006-09 | Southwestern | Community | CS | Conv | FSWs Sera collected in 2007 (fourth survey) | ELISA | 255 | 54.51 |
| Wang, 2012e | 2006-09 | Southwestern | Community | CS | Conv | FSWs Sera collected in 2008 (sixth survey) | ELISA | 281 | 63.35 |
| Wang, 2012e | 2006-09 | Southwestern | Community | CS | Conv | FSWs Sera collected in 2009 (eighth survey) | ELISA | 251 | 60.16 |
| Wang, 2012f | 2009-09 | Southwestern | Community | CS | Conv | FSWs | ELISA | 345 | 58.26 |
| Wang, 2013a | 2006-09 | Southwestern | Community | CS | Conv | FSWs | ELISA | 2282 | 62.62 |
| Wang, 2017a | 2014 | Eastern | Community | CS | TLS | FSWs | ELISA | 347 | 38.33 |
| Wang, 2019c | 2017 | Southwestern | Community | CS | Conv | FSWs with HIV | ELISA | 225 | 16.44 |
| Wang, 2023a | 2021-22 | North | Outpatient clinics | Cohort | Conv | Sexual minority women | ELISA | 219 | 5.48 |
| Wei, 2004 | 1999 | Central-southern | Community | CS | Conv | FSWs | ELISA | 101 | 29.70 |
| Wei, 2014 | 2009-12 | Northeastern | Outpatient clinics | CS | Conv | HIV-infected MSM | ELISA | 307 | 44.63 |
| Wu, 2013b | 2012-13 | Eastern | Community | CS | Conv | 17-19 years old FSWs | ELISA | 15 | 80.00 |
| Wu, 2013b | 2012-13 | Eastern | Community | CS | Conv | 20-29 years old FSWs | ELISA | 103 | 54.37 |
| Wu, 2013b | 2012-13 | Eastern | Community | CS | Conv | 30-39 years old FSWs | ELISA | 105 | 79.05 |
| Wu, 2013b | 2012-13 | Eastern | Community | CS | Conv | 40-49 years old FSWs | ELISA | 47 | 76.60 |
| Wu, 2013b | 2012-13 | Eastern | Community | CS | Conv | >50 years old FSWs | ELISA | 19 | 68.42 |
| Xie, 2021 | 2019 | Eastern | Community | CS | Conv | MSM | ELISA | 837 | 5.38 |
| Xu, 2008 | 2006 | Southwestern | Community | CS | Conv | FSWs | ELISA | 96 | 70.83 |
| Xu, 2011 | 2006-07 | Southwestern | Community | Cohort | Conv | FSWs | ELISA | 1642 | 66.93 |
| Xu, 2016 | 2012-13 | Northeastern | Community | CS | SS | MSM in Shenyang | ELISA | 587 | 16.70 |
| Xu, 2016 | 2012-13 | Central-southern | Community | CS | SS | MSM in Changsha | ELISA | 690 | 14.20 |
| Xu, 2016 | 2012-13 | Southwestern | Community | CS | SS | MSM in Kunming | ELISA | 634 | 14.20 |
| Xu, 2016 | 2012-13 | Eastern | Community | CS | SS | MSM in Shanghai | ELISA | 779 | 11.30 |
| Xu, 2016 | 2012-13 | Eastern | Community | CS | SS | MSM in Nanjing | ELISA | 593 | 10.79 |
| Xu, 2016 | 2012-13 | Eastern | Community | CS | SS | MSM in Ji'nan | ELISA | 670 | 9.10 |
| Xu, 2016 | 2012-13 | Central-southern | Community | CS | SS | MSM in Zhengzhou | ELISA | 469 | 11.30 |
| Yan, 2013 | 2010 | Eastern | Community | CS | Conv | 18-21 years old FSWs | ELISA | 72 | 30.56 |
| Yan, 2013 | 2010 | Eastern | Community | CS | Conv | 21-26 years old FSWs | ELISA | 205 | 26.34 |
| Yan, 2013 | 2010 | Eastern | Community | CS | Conv | 26-31 years old FSWs | ELISA | 121 | 28.93 |
| Yan, 2013 | 2010 | Eastern | Community | CS | Conv | ≥31 years old FSWs | ELISA | 80 | 35.00 |
| Yan, 2014a | 2012-13 | Eastern | Community | CS | Conv | MSM | ELISA | 535 | 22.06 |
| Yan, 2016 | 2008 | Eastern | Community | CS | RDS | 2008 survey among MSM | ELISA | 430 | 18.60 |
| Yan, 2016 | 2012 | Eastern | Community | CS | RDS | 2012 survey among MSM | ELISA | 589 | 10.19 |
| Yang, 2011 | 2008-09 | Eastern | Community | CS | Conv | 16-20 years old FSWs | ELISA | 126 | 22.22 |
| Yang, 2011 | 2008-09 | Eastern | Community | CS | Conv | 21-30 years old FSWs | ELISA | 467 | 48.18 |
| Yang, 2011 | 2008-09 | Eastern | Community | CS | Conv | 31-40 years old FSWs | ELISA | 178 | 60.67 |
| Yang, 2011 | 2008-09 | Eastern | Community | CS | Conv | 41-52 years old FSWs | ELISA | 22 | 63.64 |
| Yao, 2012 | 2007 | Southwestern | Community | CS | Conv | FSWs | ELISA | 397 | 42.57 |
| Ye, 2022 | 2017 | Eastern | Other | CS | CRS | Male MA users | ELISA | 464 | 26.72 |
| Ye, 2022 | 2017 | Eastern | Other | CS | CRS | Female MA users | ELISA | 168 | 70.24 |
| Yin, 2012 | 2009-10 | Central-southern | Outpatient clinics | CS | Conv | MSM from STD clinic in Shenzhen | ELISA | 648 | 18.67 |
| Yin, 2012 | 2009-10 | Central-southern | Outpatient clinics | CS | Conv | MSM from health center in Guangzhou | ELISA | 393 | 8.14 |
| Yin, 2012 | 2009-10 | Eastern | Outpatient clinics | CS | Conv | MSM from community venues in Changzhou | ELISA | 421 | 19.24 |
| Yu, 2011 | 2009-10 | North | Community | CS | Conv | ≥25 years old MSM | ELISA | 411 | 2.19 |
| Yu, 2011 | 2009-10 | North | Community | CS | Conv | <25 years old MSM | ELISA | 551 | 7.62 |
| Zhang, 2007b | 2006 | Southwestern | Community | CS | Conv | ≤25 years old FSWs | ELISA | 128 | 3.13 |
| Zhang, 2007b | 2006 | Southwestern | Community | CS | Conv | >25 years old FSWs | ELISA | 34 | 23.53 |
| Zhang, 2013a | 2008-09 | Eastern | Community | CS | SS | MSM | ELISA | 208 | 18.27 |
| Zhang, 2014a | 2011 | Eastern | Community | CS | RS | 18-25 years old FSWs | ELISA | 336 | 46.43 |
| Zhang, 2014a | 2011 | Eastern | Community | CS | RS | 26-35 years old FSWs | ELISA | 196 | 59.18 |
| Zhang, 2014a | 2011 | Eastern | Community | CS | RS | >35 years old FSWs | ELISA | 68 | 60.29 |
| Zhang, 2016 | 2012-13 | Central-southern | Community | CS | Conv | MSM | ELISA | 826 | 7.02 |
| Zhang, 2021c | 2018-19 | Southwestern | Community | CS | SS | 15-25 years old MSM | ELISA | 297 | 10.77 |
| Zhang, 2021c | 2018-19 | Southwestern | Community | CS | SS | 26-35 years old MSM | ELISA | 193 | 9.84 |
| Zhang, 2021c | 2018-19 | Southwestern | Community | CS | SS | >35 years old MSM | ELISA | 87 | 26.44 |
| Zhu, 2008 | － | Eastern | Community | CS | Conv | MSM | ELISA | 73 | 15.07 |
| Zhu, 2009 | 2007-08 | Eastern | Community | CS | TLS | MSM | ELISA | 212 | 10.85 |
| Zhu, 2017 | 2014-15 | Southwestern | Community | CS | Conv | 16-18 years old FSWs | ELISA | 167 | 38.32 |
| Zhu, 2017 | 2014-15 | Southwestern | Community | CS | Conv | 18-25 years old FSWs | ELISA | 466 | 50.00 |
| Zhu, 2017 | 2014-15 | Southwestern | Community | CS | Conv | >25 years old FSWs | ELISA | 425 | 56.47 |
| Zhu, 2018b | 2014 | Southwestern | Community | CS | SS | FSWs | ELISA | 585 | 45.81 |

^a^ The reported study design is the original study design (case control, cross sectional, cohort, or randomized controlled trial). The included seroprevalence measures are those for the baseline measures at the beginning of the study.

Abbreviations: CC = Case-control,, CS = Cross-sectional, Conv = Convenience, CRS = Cluster random sampling, RDS = Respondent driven sampling, RS = Random sampling, SS = Snowball sampling,, TLS=Time-location sampling, ELISA = Enzyme-linked immunosorbent type-specific assay, FSWs = Female sex workers, HIV = Human immunodeficiency virus, HSV-2 = Herpes simplex virus type 2, MA =Methamphetamine, CSW = Commercial sex worker, MSM = Men who have sex with men, STD = Sexually transmitted disease, WB = Western blot,.

# **Supplementary Table S6.** Studies reporting HSV-2 seroprevalence among HIV positive population and individual in HIV discordant couples in China.

| **Author, year** | **Year(s) of data collection** | **Region** | **Study site** | **Original study design^a^** | **Sampling method** | **Population** | **HSV-2 serological assay** | **Sample size** | **HSV-2 seroprevalence (%)** |
| --- | --- | --- | --- | --- | --- | --- | --- | --- | --- |
| Chen, 2010a | 2004-07 | Middle | Community | CS | Conv | < 11 years old AIDS patients | ELISA | 5 | 40.00 |
| Chen, 2010a | 2004-07 | Middle | Community | CS | Conv | 20–36 years old AIDS patients | ELISA | 31 | 12.90 |
| Chen, 2010a | 2004-07 | Middle | Community | CS | Conv | ≥36 years old AIDS patients | ELISA | 159 | 12.58 |
| Chen, 2010b | 2008-09 | Multiple | Outpatient clinics | CS | Conv | 19–29 years old HIV/AIDS patients | ELISA | 192 | 43.75 |
| Chen, 2010b | 2008-09 | Multiple | Outpatient clinics | CS | Conv | 30–39 years old HIV/AIDS patients | ELISA | 503 | 34.79 |
| Chen, 2010b | 2008-09 | Multiple | Outpatient clinics | CS | Conv | 40–49 years old HIV/AIDS patients | ELISA | 291 | 30.24 |
| Chen, 2010b | 2008-09 | Multiple | Outpatient clinics | CS | Conv | ≥50 years old HIV/AIDS patients | ELISA | 126 | 25.40 |
| Ding, 2016 | 2009-11 | Western | Community | Cohort | Conv | HIV infected partners | ELISA | 1167 | 34.88 |
| Ding, 2016 | 2009-11 | Western | Community | Cohort | Conv | HIV uninfected partners | ELISA | 1052 | 28.04 |
| Fu, 2009 | 2008 | Western | Other | CS | Conv | <40 years old HIV-infected people | ELISA | 184 | 34.24 |
| Fu, 2009 | 2008 | Western | Other | CS | Conv | ≥40 years old HIV-infected people | ELISA | 116 | 36.21 |
| Gu, 2004 | 2001-02 | Eastern | Outpatient clinics | CS | RS | HIV/AIDS patients | ELISA | 14 | 50.00 |
| He, 2011 | 2008-09 | Multiple | Outpatient clinics | CS | Conv | 19-29 years old HIV patients | ELISA | 191 | 43.98 |
| He, 2011 | 2008-09 | Multiple | Outpatient clinics | CS | Conv | 30-39 years old HIV patients | ELISA | 503 | 34.79 |
| He, 2011 | 2008-09 | Multiple | Outpatient clinics | CS | Conv | 40-49 years old HIV patients | ELISA | 290 | 30.34 |
| He, 2011 | 2008-09 | Multiple | Outpatient clinics | CS | Conv | 50-59 years old HIV patients | ELISA | 96 | 20.83 |
| He, 2011 | 2008-09 | Multiple | Outpatient clinics | CS | Conv | 60-94 years old HIV patients | ELISA | 30 | 40.00 |
| Liu, 2017a | 2016 | Multiple | Outpatient clinics | CS | Conv | HIV-positive MSM | ELISA | 580 | 0.52 |
| Mu, 2011 | 2009 | Western | Outpatient clinics | CS | Conv | < 20 years old drug-addicted HIV/AIDS patients | ELISA | 2 | 50.00 |
| Mu, 2011 | 2009 | Western | Outpatient clinics | CS | Conv | 20-30 years old drug-addicted HIV/AIDS patients | ELISA | 25 | 56.00 |
| Mu, 2011 | 2009 | Western | Outpatient clinics | CS | Conv | 30-40 years old drug-addicted HIV/AIDS patients | ELISA | 29 | 65.52 |
| Mu, 2011 | 2009 | Western | Outpatient clinics | CS | Conv | ≥40 years old HIV/AIDS patients | ELISA | 11 | 63.64 |
| Tu, 2022 | 2020-21 | Western | Outpatient clinics | CS | CRS | HIV-positive patients | ELISA | 406 | 33.25 |
| Wang, 2014a | 2013 | Western | Outpatient clinics | CS | Conv | 21-30 years old HIV/AIDS patients | ELISA | 60 | 3.33 |
| Wang, 2014a | 2013 | Western | Outpatient clinics | CS | Conv | 31-40 years old HIV/AIDS patients | ELISA | 51 | 1.96 |
| Wang, 2014a | 2013 | Western | Outpatient clinics | CS | Conv | 41-50 years old HIV/AIDS patients | ELISA | 45 | 2.22 |
| Yang, 2013a | 2012 | Eastern | Community | CS | CRS | ≤35 years old HIV-infected MSM | ELISA | 123 | 20.33 |
| Yang, 2013a | 2012 | Eastern | Community | CS | CRS | ＞35 years old HIV-infected MSM | ELISA | 141 | 34.75 |
| Yang, 2015 | 2006-08 | Eastern | Outpatient clinics | CS | Conv | <20 years old VCT Outpatient HIV Testers | ELISA | 26 | 19.23 |
| Yang, 2015 | 2006-08 | Eastern | Outpatient clinics | CS | Conv | 20-29 years old VCT Outpatient HIV Testers | ELISA | 645 | 11.01 |
| Yang, 2015 | 2006-08 | Eastern | Outpatient clinics | CS | Conv | 30-39 years old VCT Outpatient HIV Testers | ELISA | 302 | 12.58 |
| Yang, 2015 | 2006-08 | Eastern | Outpatient clinics | CS | Conv | >40 years old VCT Outpatient HIV Testers | ELISA | 96 | 21.88 |
| Zhang, 2021d | 2009-14 | Multiple | Other | Cohort | Conv | MSM with acute HIV infection | ELISA | 400 | 27.25 |
| Zhu, 2013 | NA | Western | Community | CS | Conv | Male HIV-positive drug users | ELISA | 381 | 52.76 |

^a^ The reported study design is the original study design (case control, cross sectional, cohort, or randomized controlled trial). The included seroprevalence measures are those for the baseline measures at the beginning of the study.

Abbreviations: CC = Case-control, Conv = Convenience, CRS = Cluster random sampling, CS = Cross-sectional, ELISA = Enzyme-linked immunosorbent type-specific assay, HIV = Human immunodeficiency virus, HSV-2 = Herpes simplex virus type 2, AIDS = Acquired immunodeficiency syndrome, MSM = Men who have sex with men, RS = Random sampling, VCT = Voluntary counselling and testing.

# **Supplementary Table S7.** Studies reporting HSV-2 seroprevalence among STI clinic attendees and symptomatic populations in China.

| **Author, year** | **Year(s) of data collection** | **Region** | **Study site** | **Original study design^a^** | **Sampling method** | **Population** | **HSV-2 serological assay** | **Sample size** | **HSV-2 seroprevalence (%)** |
| --- | --- | --- | --- | --- | --- | --- | --- | --- | --- |
| Bi, 2006 | 2003-05 | Eastern | Outpatient clinic | CS | Conv | Male gonorrhea patients | ELISA | 282 | 0.71 |
| Bi, 2006 | 2003-05 | Eastern | Outpatient clinic | CS | Conv | Female gonorrhea patients | ELISA | 59 | 0.00 |
| Chen, 2002 | 2000-01 | Middle | Outpatient clinic | CS | Conv | Male STD patients | IF | 714 | 10.50 |
| Chen, 2002 | 2000-01 | Middle | Outpatient clinic | CS | Conv | Female STD patients | IF | 421 | 11.64 |
| Chen, 2013c | 2011-12 | Eastern | Outpatient clinic | CS | Conv | ＜18 years old suspected GH patients | ELISA | 21 | 28.57 |
| Chen, 2013c | 2011-12 | Eastern | Outpatient clinic | CS | Conv | 18–40 years old suspected GH patients | ELISA | 250 | 55.20 |
| Chen, 2013c | 2011-12 | Eastern | Outpatient clinic | CS | Conv | ＞40 years old suspected GH patients | ELISA | 41 | 46.34 |
| Dai, 2009 | 2006 | Eastern | Other | CS | Conv | Women in gynecological clinics | ELISA | 283 | 18.73 |
| Dai, 2011 | 2007-08 | Eastern | Outpatient clinic | CS | Conv | Female patients | ELISA | 746 | 63.27 |
| Gao, 2011 | 2008 | Eastern | Outpatient clinic | CS | Conv | 17–25 years old STD clinics attendees | ELISA | 32 | 28.13 |
| Gao, 2011 | 2008 | Eastern | Outpatient clinic | CS | Conv | 25–35 years old STD clinics attendees | ELISA | 96 | 31.25 |
| Gao, 2011 | 2008 | Eastern | Outpatient clinic | CS | Conv | 35–45 years old STD clinics attendees | ELISA | 59 | 32.20 |
| Gao, 2011 | 2008 | Eastern | Outpatient clinic | CS | Conv | 45–86 years old STD clinics attendees | ELISA | 69 | 27.54 |
| Gu, 2004 | 2001-02 | Eastern | Outpatient clinic | CS | RS | STD patients | ELISA | 76 | 31.58 |
| Gu, 2011 | 2009-11 | Northeastern | Outpatient clinic | CS | Conv | ≤20 years old GH patients | ELISA | 15 | 53.33 |
| Gu, 2011 | 2009-11 | Northeastern | Outpatient clinic | CS | Conv | 21–30 years old GH patients | ELISA | 194 | 66.49 |
| Gu, 2011 | 2009-11 | Northeastern | Outpatient clinic | CS | Conv | 31–40 years old GH patients | ELISA | 210 | 81.90 |
| Gu, 2011 | 2009-11 | Northeastern | Outpatient clinic | CS | Conv | 41–50 years old GH patients | ELISA | 74 | 28.38 |
| Gu, 2011 | 2009-11 | Northeastern | Outpatient clinic | CS | Conv | 51–60 years old GH patients | ELISA | 27 | 55.56 |
| Gu, 2011 | 2009-11 | Northeastern | Outpatient clinic | CS | Conv | ≥60 years old GH patients | ELISA | 10 | 40.00 |
| He, 1997 | 1994-95 | Eastern | Outpatient clinic | CS | Conv | Male STD clinic patients | ELISA | 1080 | 1.67 |
| He, 1997 | 1994-95 | Eastern | Outpatient clinic | CS | Conv | Female STD clinic patients | ELISA | 468 | 0.00 |
| Huang, 2005 | 2000-04 | Eastern | Outpatient clinic | CS | Conv | Male STD clinic patients collected in 2001 | ELISA | 279 | 25.81 |
| Huang, 2005 | 2000-04 | Eastern | Outpatient clinic | CS | Conv | Male STD clinic patients collected in 2002 | ELISA | 385 | 13.25 |
| Huang, 2005 | 2000-04 | Eastern | Outpatient clinic | CS | Conv | Male STD clinic patients collected in 2003 | ELISA | 395 | 16.96 |
| Huang, 2005 | 2000-04 | Eastern | Outpatient clinic | CS | Conv | Male STD clinic patients collected in 2004 | ELISA | 336 | 19.94 |
| Huang, 2005 | 2000-04 | Eastern | Outpatient clinic | CS | Conv | Female STD clinic patients collected in 2001 | ELISA | 170 | 28.24 |
| Huang, 2005 | 2000-04 | Eastern | Outpatient clinic | CS | Conv | Female STD clinic patients collected in 2002 | ELISA | 171 | 21.05 |
| Huang, 2005 | 2000-04 | Eastern | Outpatient clinic | CS | Conv | Female STD clinic patients collected in 2003 | ELISA | 174 | 21.84 |
| Huang, 2005 | 2000-04 | Eastern | Outpatient clinic | CS | Conv | Female STD clinic patients collected in 2004 | ELISA | 119 | 34.45 |
| Huang, 2015c | 2010-13 | Eastern | Outpatient clinic | CS | Conv | Male STD clinic patients | ELISA | 278 | 37.41 |
| Jia, 2010 | 2006-08 | Northeastern | Outpatient clinic | CS | Conv | Genital inflammation | ELISA | 1009 | 26.56 |
| Kuang, 2009 | 2008-09 | Middle | Outpatient clinic | CS | Conv | Female syphilis patients | Other | 80 | 38.75 |
| Li, 2003b | 2000-01 | Middle | Outpatient clinic | CS | Conv | STD confirmed female patients | IF | 500 | 11.20 |
| Li, 2004 | 2000-01 | Eastern | Outpatient clinic | CS | Conv | 15-29 years old STD clinic patients | ELISA | 177 | 6.21 |
| Li, 2004 | 2000-01 | Eastern | Outpatient clinic | CS | Conv | 30-49 years old STD clinic patients | ELISA | 459 | 21.57 |
| Li, 2004 | 2000-01 | Eastern | Outpatient clinic | CS | Conv | 50-70 years old STD clinic patients | ELISA | 278 | 12.95 |
| Li, 2007a | 2006 | Eastern | Outpatient clinic | CS | Conv | Male STD patients | ELISA | 260 | 59.23 |
| Li, 2007a | 2006 | Eastern | Outpatient clinic | CS | Conv | Female STD patients | ELISA | 341 | 61.58 |
| Li, 2012b | 2006-09 | Western | Outpatient clinic | CS | Conv | 18-30 years old suspected GH patients | Other | 118 | 23.73 |
| Li, 2012b | 2006-09 | Western | Outpatient clinic | CS | Conv | 31-40 years old suspected GH patients | Other | 206 | 43.69 |
| Li, 2012b | 2006-09 | Western | Outpatient clinic | CS | Conv | 41-49 years old suspected GH patients | Other | 96 | 22.92 |
| Li, 2012c | 2011 | Northeastern | Outpatient clinic | CS | Conv | Male STD patients | ELISA | 186 | 59.14 |
| Li, 2012c | 2011 | Northeastern | Outpatient clinic | CS | Conv | Female STD patients | ELISA | 120 | 60.83 |
| Li, 2013b | 2009-12 | Northeastern | Outpatient clinic | CS | Conv | Male STD patients | ELISA | 1418 | 32.65 |
| Li, 2013b | 2009-12 | Northeastern | Outpatient clinic | CS | Conv | Female STD patients | ELISA | 882 | 53.29 |
| Li, 2014c | 2011-12 | Western | Outpatient clinic | CS | Conv | ≤18 years old STD clinic patients | ELISA | 26 | 11.54 |
| Li, 2014c | 2011-12 | Western | Outpatient clinic | CS | Conv | 18-35 years old STD clinic patients | ELISA | 506 | 27.08 |
| Li, 2014c | 2011-12 | Western | Outpatient clinic | CS | Conv | 36-54 years old STD clinic patients | ELISA | 313 | 37.70 |
| Li, 2014c | 2011-12 | Western | Outpatient clinic | CS | Conv | ≥55 years old STD clinic patients | ELISA | 56 | 37.50 |
| Li, 2015 | 2014 | Northeastern | Outpatient clinic | CS | Conv | Male STD clinic patients | ELISA | 312 | 21.79 |
| Li, 2015 | 2014 | Northeastern | Outpatient clinic | CS | Conv | Female STD clinic patients | ELISA | 270 | 48.15 |
| Lin, 2002 | 1999-00 | Eastern | Outpatient clinic | CS | Conv | Patients with STDs | Other | 94 | 68.09 |
| Lin, 2009b | 2004-08 | Eastern | Outpatient clinic | CS | Conv | Patients with suspected genitourinary tract infection | Other | 186 | 12.90 |
| Liu, 2006 | 2004-05 | Eastern | Outpatient clinic | CS | Conv | Male suspected infected patients | Other | 1642 | 2.25 |
| Liu, 2006 | 2004-05 | Eastern | Outpatient clinic | CS | Conv | Female suspected infected patients | Other | 1329 | 0.30 |
| Lo, 1999 | 1995 | Hong Kong | Outpatient clinic | CS | Conv | Female STD clinic attenees | WB | 76 | 35.53 |
| Lo, 1999 | 1995 | Hong Kong | Outpatient clinic | CS | Conv | Male STD clinic attendees | WB | 74 | 24.32 |
| Lu, 2002 | 2000-01 | Eastern | Other | CS | Conv | Patients with STDs | ELISA | 297 | 20.88 |
| Lu, 2004 | 1999-02 | Western | Outpatient clinic | CS | Conv | Female STD clinic attendees | ELISA | 827 | 2.78 |
| Pang, 2015 | 2013-14 | Eastern | Outpatient clinic | CS | Conv | <20 years old suspected GH patients | ELISA | 45 | 0.00 |
| Pang, 2015 | 2013-14 | Eastern | Outpatient clinic | CS | Conv | 20-29 years old suspected GH patients | ELISA | 310 | 16.77 |
| Pang, 2015 | 2013-14 | Eastern | Outpatient clinic | CS | Conv | 30-39 years old suspected GH patients | ELISA | 369 | 25.75 |
| Pang, 2015 | 2013-14 | Eastern | Outpatient clinic | CS | Conv | 40-49 years old suspected GH patients | ELISA | 163 | 35.58 |
| Pang, 2015 | 2013-14 | Eastern | Outpatient clinic | CS | Conv | ≥50 years old suspected GH patients | ELISA | 87 | 25.29 |
| Peng, 2001 | — | Eastern | Outpatient clinic | CS | Conv | patients with nongonococcal urethritis(NGU) | Other | 168 | 20.83 |
| Shao, 2010 | 2007 | Eastern | Outpatient clinic | CS | Conv | <20 years old STD clinics attendees | ELISA | 19 | 36.84 |
| Shao, 2010 | 2007 | Eastern | Outpatient clinic | CS | Conv | 20-35 years old STD clinics attendees | ELISA | 173 | 17.34 |
| Shao, 2010 | 2007 | Eastern | Outpatient clinic | CS | Conv | 36-50 years old STD clinics attendees | ELISA | 73 | 21.92 |
| Shao, 2010 | 2007 | Eastern | Outpatient clinic | CS | Conv | >50 years old STD clinics attendees | ELISA | 26 | 11.54 |
| Shen, 2009 | 2008 | Eastern | Outpatient clinic | CS | Conv | ≤25 years old STD clinics attendees | ELISA | 95 | 22.11 |
| Shen, 2009 | 2008 | Eastern | Outpatient clinic | CS | Conv | 26-35 years old STD clinics attendees | ELISA | 151 | 15.89 |
| Shen, 2009 | 2008 | Eastern | Outpatient clinic | CS | Conv | 36-45 years old STD clinics attendees | ELISA | 96 | 18.75 |
| Shen, 2009 | 2008 | Eastern | Outpatient clinic | CS | Conv | ≥46 years old STD clinics attendees | ELISA | 77 | 22.08 |
| Shen, 2014 | 2011-12 | Eastern | Outpatient clinic | CS | Conv | ≤20 years old STD clinics attendees | ELISA | 6 | 66.67 |
| Shen, 2014 | 2011-12 | Eastern | Outpatient clinic | CS | Conv | 21-30 years old STD clinics attendees | ELISA | 97 | 63.92 |
| Shen, 2014 | 2011-12 | Eastern | Outpatient clinic | CS | Conv | 31-40 years old STD clinics attendees | ELISA | 105 | 81.90 |
| Shen, 2014 | 2011-12 | Eastern | Outpatient clinic | CS | Conv | 41-50 years old STD clinics attendees | ELISA | 37 | 29.73 |
| Shen, 2014 | 2011-12 | Eastern | Outpatient clinic | CS | Conv | 51-60 years old STD clinics attendees | ELISA | 13 | 61.54 |
| Shen, 2014 | 2011-12 | Eastern | Outpatient clinic | CS | Conv | ≥60 years old STD clinics attendees | ELISA | 7 | 57.14 |
| Su, 2019 | 2015-17 | Northeastern | Outpatient clinic | CS | Conv | Male patients with suspected genital herpes | ELISA | 800 | 20.75 |
| Su, 2019 | 2015-17 | Northeastern | Outpatient clinic | CS | Conv | Female patients with suspected genital herpes | ELISA | 800 | 47.00 |
| Tan, 2001 | — | Middle | Outpatient clinic | CS | Conv | Male STD patients | ELISA | 182 | 11.54 |
| Tan, 2001 | — | Middle | Outpatient clinic | CS | Conv | Female STD patients | ELISA | 145 | 14.48 |
| Tan, 2001 | — | Middle | Outpatient clinic | CS | Conv | Male health control group | ELISA | 20 | 5.00 |
| Tan, 2001 | — | Middle | Outpatient clinic | CS | Conv | Female health control group | ELISA | 10 | 0.00 |
| Wang, 2014b | 2013 | Eastern | Outpatient clinic | CS | Conv | Male <20 years old dermatology 1s | ELISA | 71 | 1.41 |
| Wang, 2014b | 2013 | Eastern | Outpatient clinic | CS | Conv | Male 20-29 years old dermatology 1s | ELISA | 620 | 7.42 |
| Wang, 2014b | 2013 | Eastern | Outpatient clinic | CS | Conv | Male 30-39 years old dermatology 1s | ELISA | 716 | 14.66 |
| Wang, 2014b | 2013 | Eastern | Outpatient clinic | CS | Conv | Male 40-49 years old dermatology 1s | ELISA | 469 | 18.12 |
| Wang, 2014b | 2013 | Eastern | Outpatient clinic | CS | Conv | Male 50-59 years old dermatology 1s | ELISA | 213 | 20.66 |
| Wang, 2014b | 2013 | Eastern | Outpatient clinic | CS | Conv | Male ≥60 years old dermatology 1s | ELISA | 169 | 14.20 |
| Wang, 2014b | 2013 | Eastern | Outpatient clinic | CS | Conv | Female <20 years old dermatology 1s | ELISA | 46 | 13.04 |
| Wang, 2014b | 2013 | Eastern | Outpatient clinic | CS | Conv | Female 20-29 years old dermatology 1s | ELISA | 299 | 24.08 |
| Wang, 2014b | 2013 | Eastern | Outpatient clinic | CS | Conv | Female 30-39 years old dermatology 1s | ELISA | 263 | 38.02 |
| Wang, 2014b | 2013 | Eastern | Outpatient clinic | CS | Conv | Female 40-49 years old dermatology 1s | ELISA | 149 | 38.93 |
| Wang, 2014b | 2013 | Eastern | Outpatient clinic | CS | Conv | Female 50-59 years old dermatology 1s | ELISA | 92 | 32.61 |
| Wang, 2014b | 2013 | Eastern | Outpatient clinic | CS | Conv | Female ≥60 years old dermatology 1s | ELISA | 60 | 26.67 |
| Wang, 2018b | 2013-16 | Western | Outpatient clinic | CS | Conv | Male STD clinics attendees | ELISA | 2241 | 2.86 |
| Wang, 2018b | 2013-16 | Western | Outpatient clinic | CS | Conv | Famale STD clinics attendees | ELISA | 670 | 5.07 |
| Wang, 2020 | 2015-17 | Eastern | Outpatient clinic | CS | Conv | Male patients newly diagnosed with anogenital warts | ELISA | 117 | 4.27 |
| Wang, 2020 | 2015-17 | Eastern | Outpatient clinic | CS | Conv | Female patients newly diagnosed with anogenital warts | ELISA | 83 | 8.43 |
| Wang, 2021b | 2018-19 | Western | Outpatient clinic | CS | Conv | <20 years old Suspected GH patients | ELISA | 54 | 5.56 |
| Wang, 2021b | 2018-19 | Western | Outpatient clinic | CS | Conv | 20-40 years old Suspected GH patients | ELISA | 423 | 22.22 |
| Wang, 2021b | 2018-19 | Western | Outpatient clinic | CS | Conv | 41-60 years old Suspected GH patients | ELISA | 366 | 29.78 |
| Wang, 2021b | 2018-19 | Western | Outpatient clinic | CS | Conv | >60 years old Suspected GH patients | ELISA | 193 | 18.13 |
| Wei, 2006 | 2005 | Eastern | Outpatient clinic | CS | Conv | STD clinics attendees | ELISA | 860 | 3.72 |
| Wu, 2013c | 2011-12 | Western | Outpatient clinic | CS | Conv | <18 years old STD outpatients | ELISA | 26 | 11.54 |
| Wu, 2013c | 2011-12 | Western | Outpatient clinic | CS | Conv | 18 -35 years old STD outpatients | ELISA | 506 | 27.08 |
| Wu, 2013c | 2011-12 | Western | Outpatient clinic | CS | Conv | 36-54 years old STD outpatients | ELISA | 313 | 37.70 |
| Wu, 2013c | 2011-12 | Western | Outpatient clinic | CS | Conv | >55 years old STD outpatients | ELISA | 56 | 37.50 |
| Wu, 2013c | 2011-12 | Western | Outpatient clinic | CS | Conv | <18 years old STD inpatients | ELISA | 96 | 4.17 |
| Wu, 2013c | 2011-12 | Western | Outpatient clinic | CS | Conv | 18 -35 years old STD inpatients | ELISA | 128 | 10.94 |
| Wu, 2013c | 2011-12 | Western | Outpatient clinic | CS | Conv | 36-54 years old STD inpatients | ELISA | 225 | 13.33 |
| Wu, 2013c | 2011-12 | Western | Outpatient clinic | CS | Conv | >55 years old STD inpatients | ELISA | 325 | 6.77 |
| Yang, 2012a | 2009-10 | Eastern | Outpatient clinic | CS | Conv | Pregnancy with symptomatic and doubtful genital herpes | ELISA | 2539 | 48.01 |
| Yin, 2005 | — | Eastern | Outpatient clinic | CS | Conv | STD clinic attendees | WB | 105 | 55.24 |
| Zeng, 2006 | 2004-05 | Middle | Outpatient clinic | CS | Conv | Dermatovenereal and gynecological 1s | ELISA | 85 | 30.59 |
| Zhang, 2002a | — | Eastern | Outpatient clinic | CS | Conv | Patients with cervical lesions | ELISA | 148 | 25.68 |
| Zhang, 2010c | — | Eastern | Outpatient clinic | CS | Conv | Male STD clinics attendees | Not reported | 450 | 18.2 |
| Zhang, 2015 | 2012 | Eastern | Other | CS | RS | Port entry population | ELISA | 1041 | 19.79 |
| Zhang, 2021e | 2017-18 | Eastern | Outpatient clinic | CS | Conv | Patients attending a STI clinic | ELISA | 678 | 13.57 |
| Zhong, 2008b | 2007 | Eastern | Outpatient clinic | CS | Conv | 17-24 years old STD clinic patients | ELISA | 124 | 23.39 |
| Zhong, 2008b | 2007 | Eastern | Outpatient clinic | CS | Conv | 25-34 years old STD clinic patients | ELISA | 234 | 24.36 |
| Zhong, 2008b | 2007 | Eastern | Outpatient clinic | CS | Conv | 35-44 years old STD clinic patients | ELISA | 120 | 23.33 |
| Zhong, 2008b | 2007 | Eastern | Outpatient clinic | CS | Conv | 45-86 years old STD clinic patients | ELISA | 138 | 23.91 |
| Zhong, 2010 | 2007 | Eastern | Outpatient clinic | CS | Conv | 17-24 years old VCT clinic patients | ELISA | 74 | 6.76 |
| Zhong, 2010 | 2007 | Eastern | Outpatient clinic | CS | Conv | 25-34 years old VCT clinic patients | ELISA | 168 | 8.93 |
| Zhong, 2010 | 2007 | Eastern | Outpatient clinic | CS | Conv | 35-44 years old VCT clinic patients | ELISA | 30 | 20.00 |
| Zhong, 2010 | 2007 | Eastern | Outpatient clinic | CS | Conv | 45-86 years old VCT clinic patients | ELISA | 19 | 5.26 |
| Zhou, 2007b | 2002-06 | Western | Outpatient clinic | CS | Conv | STD clinic patients | ELISA | 470 | 16.38 |
| Zhou, 2008 | — | Eastern | Outpatient clinic | CS | Conv | Male STD clinic patients | ELISA | 441 | 32.88 |
| Zhou, 2008 | — | Eastern | Outpatient clinic | CS | Conv | Female STD clinic patients | ELISA | 71 | 71.83 |
| Zhou, 2011b | 2008-11 | Western | Outpatient clinic | CS | Conv | Male STD clinic patients | IF | 557 | 22.26 |
| Zhou, 2011b | 2008-11 | Western | Outpatient clinic | CS | Conv | Female STD clinic patients | IF | 983 | 62.67 |
| Zhou, 2013a | 2011 | Eastern | Outpatient clinic | CS | Conv | 50-60 years old male STD clinic patients | ELISA | 91 | 30.77 |
| Zhou, 2013a | 2011 | Eastern | Outpatient clinic | CS | Conv | ≥61 years old male STD clinic patients | ELISA | 66 | 36.36 |
| Zou, 2015 | 2014 | Eastern | Outpatient clinic | CS | Conv | 17-20 years old STD clinic patients | ELISA | 39 | 10.26 |
| Zou, 2015 | 2014 | Eastern | Outpatient clinic | CS | Conv | 21-30 years old STD clinic patients | ELISA | 440 | 19.32 |
| Zou, 2015 | 2014 | Eastern | Outpatient clinic | CS | Conv | 31-40 years old STD clinic patients | ELISA | 411 | 28.95 |
| Zou, 2015 | 2014 | Eastern | Outpatient clinic | CS | Conv | 40-50 years old STD clinic patients | ELISA | 205 | 25.85 |
| Zou, 2015 | 2014 | Eastern | Outpatient clinic | CS | Conv | >50 years old STD clinic patients | ELISA | 120 | 25.00 |

^a^ The reported study design is the original study design (case control, cross sectional, cohort, or randomized controlled trial). The included seroprevalence measures are those for the baseline measures at the beginning of the study.

Abbreviations:CS = Cross-sectional, Conv = Convenience, RS = Random sampling,, GH = Genital herpes, STI = Sexually transmitted infection, STD = Sexually transmitted disease, VCT = Voluntary counselling and testing,, ELISA = Enzyme-linked immunosorbent type-specific assay, IF = Immunofluorescent, PCR = Polymerase chain reaction, HIV = Human immunodeficiency virus, HSV-2 = Herpes simplex virus type 2, WB = Western blot.

# **Supplementary Table S8.** Studies reporting HSV-2 seroprevalence among other populations in China.

| **Author, year** | **Year(s) of data collection** | **Region** | **Study site** | **Original study design**a | **Sampling method** | **Population** | **HSV-2 serological assay** | **Sample size** | **HSV-2 seroprevalence (%)** |
| --- | --- | --- | --- | --- | --- | --- | --- | --- | --- |
| Liu, 2004a | 2000-03 | Eastern | Inpatient | CS | Conv | 14-30 years old Hospitalized patients with adult acute respiratory infection | ELISA | 241 | 3.32 |
| Liu, 2004a | 2000-03 | Eastern | Inpatient | CS | Conv | 31-60 years old Hospitalized patients with adult acute respiratory infection | ELISA | 406 | 1.23 |
| Liu, 2004a | 2000-03 | Eastern | Inpatient | CS | Conv | 61-100 years old Hospitalized patients with adult acute respiratory infection | ELISA | 158 | 3.80 |
| Luo, 2002a | 2000-02 | Middle | Inpatient | CS | Conv | Hepatitis patients | ELISA | 457 | 1.75 |
| Ma, 2014 | 2010-14 | Northeastern | Inpatient | CS | Conv | Patients with acute transverse myelitis | ELISA | 64 | 39.06 |
| Ma, 2018 | 2014-17 | Eastern | Outpatient clinic | CS | Conv | Patients with neurological infectious disease | ELISA | 6283 | 4.62 |
| Peng, 1991 | 1987-88 | Western | Outpatient clinic | CC | Conv | women with invasive cervical cancer | WB | 89 | 46.07 |
| Tang, 2006 | 2004-05 | Middle | Inpatient | CS | Conv | Infant and toddler group of all children hospitalized for TORCH testing | ELISA | 1300 | 1.77 |
| Zhang, 2002a | — | Eastern | Outpatient clinic | CS | Conv | Health Screening Men | ELISA | 456 | 19.30 |
| Zhang, 2021a | 2015-19 | Middle | Inpatient | CS | Conv | Children with hematological diseases | Other | 354 | 15.82 |

^a^ The reported study design is the original study design (case control, cross sectional, cohort, or randomized controlled trial). The included seroprevalence measures are those for the baseline measures at the beginning of the study.

Abbreviations: CC = Case-control, Conv = Convenience, CS = Cross-sectional, ELISA = Enzyme-linked immunosorbent type-specific assay, HIV = Human immunodeficiency virus, HSV-2 = Herpes simplex virus type 2, TORCH = T：Toxoplasmagondii, O: Others, R: Rubellavirus, C: Cytomegalovirus, H: Herpessimplexvirus, PCR = Polymerase chain reaction, WB = Western blot.

# **Supplementary Table S9.** Distribution of publications on HSV-2 seroprevalence across different groups in China in English and Chinese databases

|  |  | **Number of measurements K (%)^a^** | | |
| --- | --- | --- | --- | --- |
|  |  | **English**  **n=60, K=194^b^** | **Chinese**  **n=342, K=664** | **p-value** |
| **Population characteristics** | |  |  |  |
| **Population type** | General population | 93 (47.9) | 395 (59.5) | <0.001 |
|  | Intermediate risk population | 13 (6.7) | 16 (2.4) |  |
|  | FSWs | 36 (18.6) | 35 (5.3) |  |
|  | MSM/MSWs | 28 (14.4) | 40 (6.0) |  |
|  | Other key populations | 8 (4.1) | 8 (1.2) |  |
|  | STI clinics attendees | 6 (3.1) | 136 (20.5) |  |
|  | HIV positive population and individual in HIV discordant couples | 9 (4.6) | 25 (3.8) |  |
|  | Other populations^e^ | 1 (0.5) | 9 (1.4) |  |
| **Age group** | <20 | 16 (8.3) | 61 (9.2) | <0.001 |
|  | 20-29 years | 30 (15.5) | 84 (12.7) |  |
|  | 30-39 years | 26 (13.4) | 49 (7.4) |  |
|  | 40-49 years | 16 (8.3) | 24 (3.6) |  |
|  | 50-59 years | 9 (4.6) | 9 (1.4) |  |
|  | 60+ years | 7 (3.6) | 9 (1.4) |  |
|  | Mixed | 90 (46.4) | 428 (64.5) |  |
| **Sex** | Women | 100 (51.6) | 402 (60.5) | <0.001 |
|  | Men | 77(39.7) | 101 (15.2) |  |
|  | Mixed sexes | 17 (8.8) | 161 (24.3) |  |
| **Regions** | North | 14 (7.2) | 52 (7.8) | <0.001 |
|  | Northeastern | 6 (3.1) | 21 (3.2) |  |
|  | Eastern | 63 (32.5) | 236 (35.5) |  |
|  | Central-southern | 14 (7.2) | 189 (28.5) |  |
|  | Southwestern | 45 (23.2) | 104 (15.7) |  |
|  | Northwestern | 4 (2.1) | 53 (8.0) |  |
|  | SARs^f^ and Taiwan | 33 (17.0) | 0 (0.0) |  |
|  | Multiple or unknown | 15 (7.7) | 9 (1.4) |  |
| **Sample size** | <200 | 11 (5.7) | 222 (33.4) | <0.001 |
|  | >=200 | 183 (94.3) | 442 (66.6) |  |
| **Sampling** | Probability | 81 (41.8) | 44 (6.6) | <0.001 |
|  | Non-probability | 113 (58.2) | 620 (93.4) |  |
| **Response rate** | >=80% | 70 (36.1) | 163 (24.6) | <0.001 |
|  | <80% | 6 (3.1) | 4 (0.6) |  |
|  | Unclear | 118 (60.8) | 497 (74.8) |  |
| **Temporal variables** | |  |  |  |
| **Year of data collection** | <=2000 | 21 (10.8) | 25 (3.8) | <0.001 |
|  | 2001-2010 | 106 (54.6) | 262 (39.5) |  |
|  | >2010 | 60 (30.9) | 324 (48.8) |  |
|  | Unknown | 7 (3.6) | 53 (8.0) |  |

^a^ N means the unique number of publications. ^b^ K means the number of total measurements on different strata. ^c^ P-value is based on chi-squared tests.

# **Supplementary Figure S1.** Forest plots presenting outcomes of the pooled mean HSV-2 seroprevalence among different populations in China.

## General populations


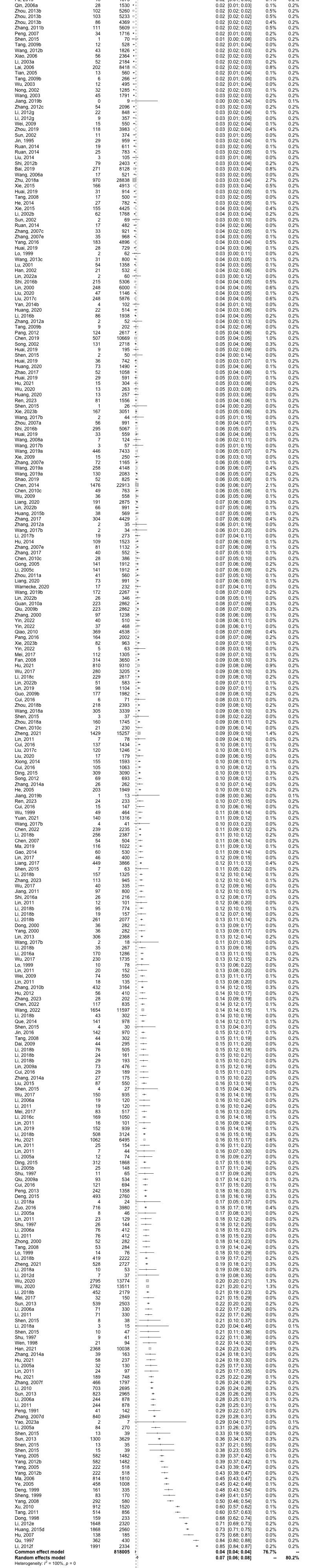


## Intermediate-risk populations


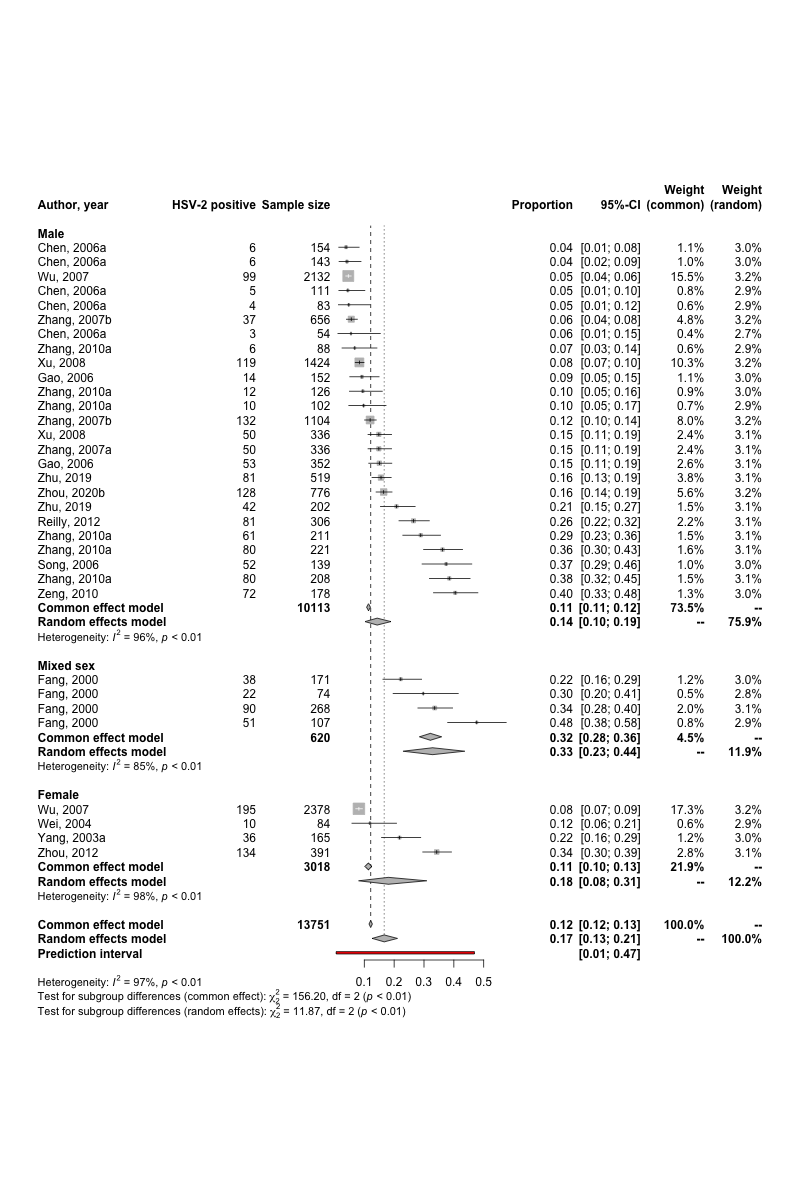


## Key populations


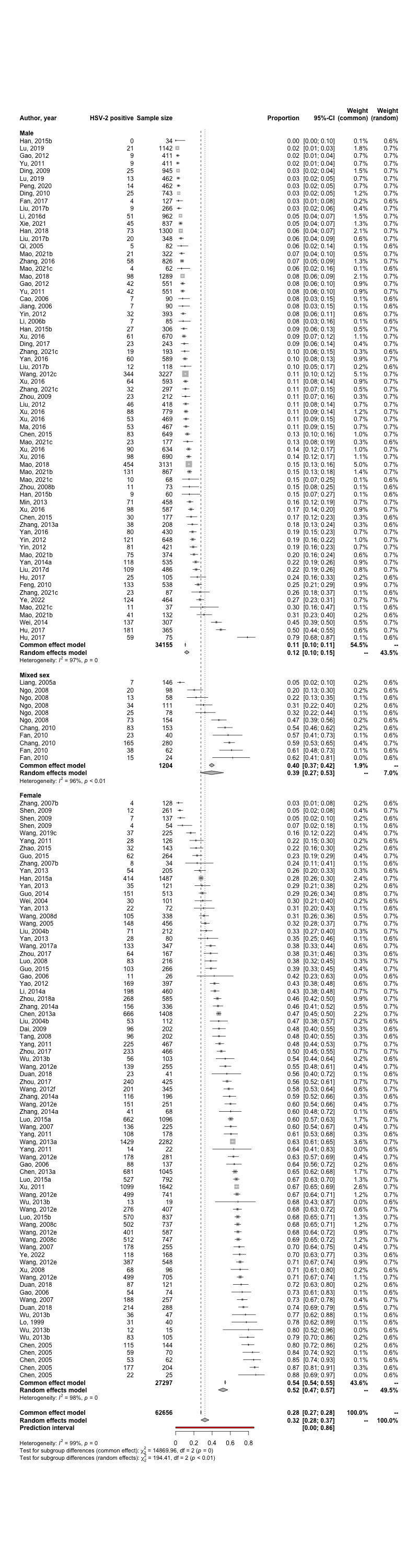


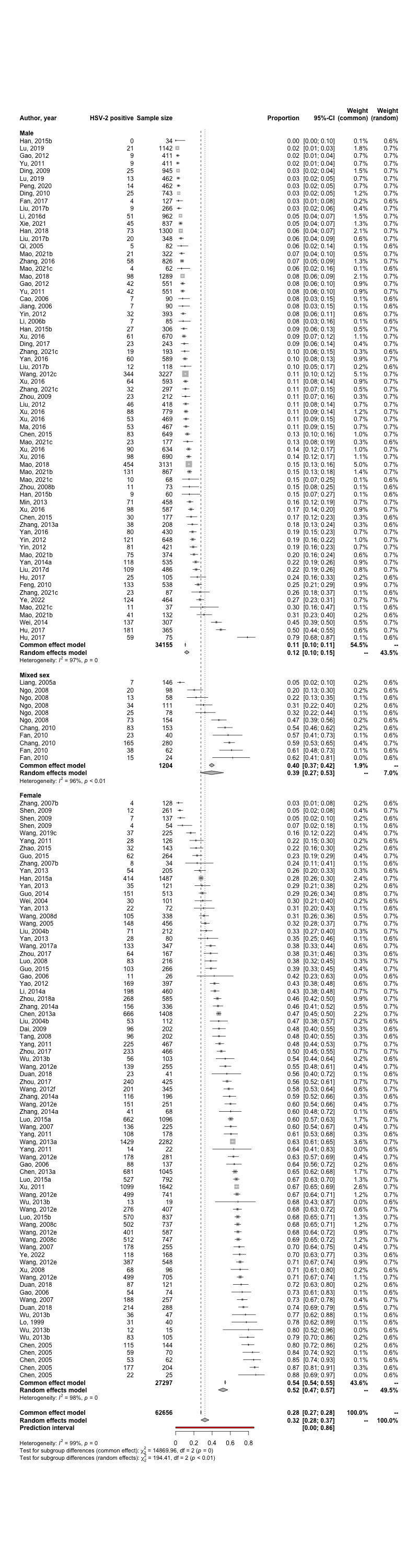


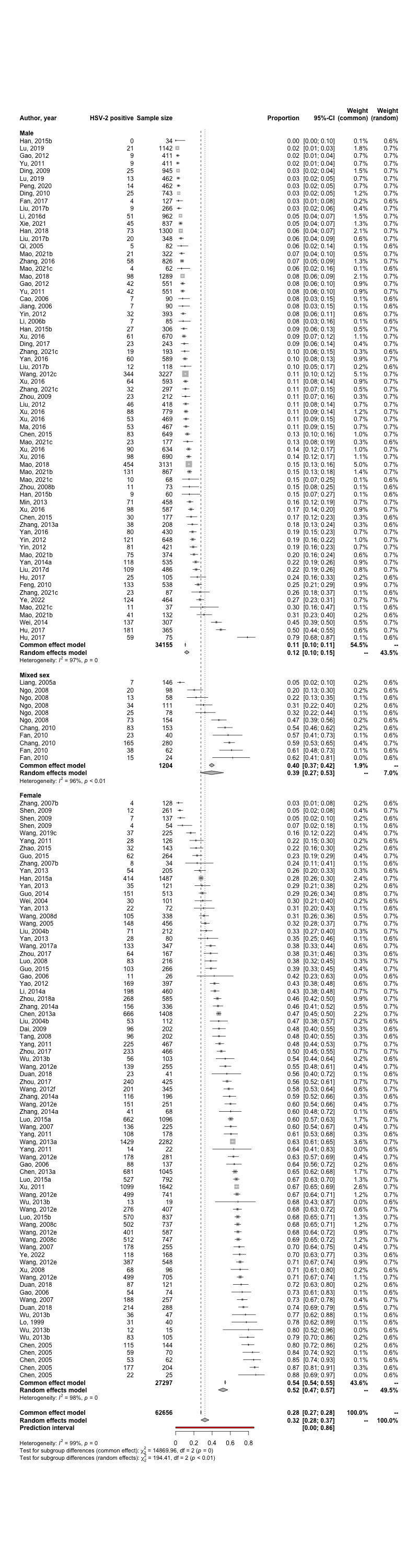


## STI clinic attendees and symptomatic populations
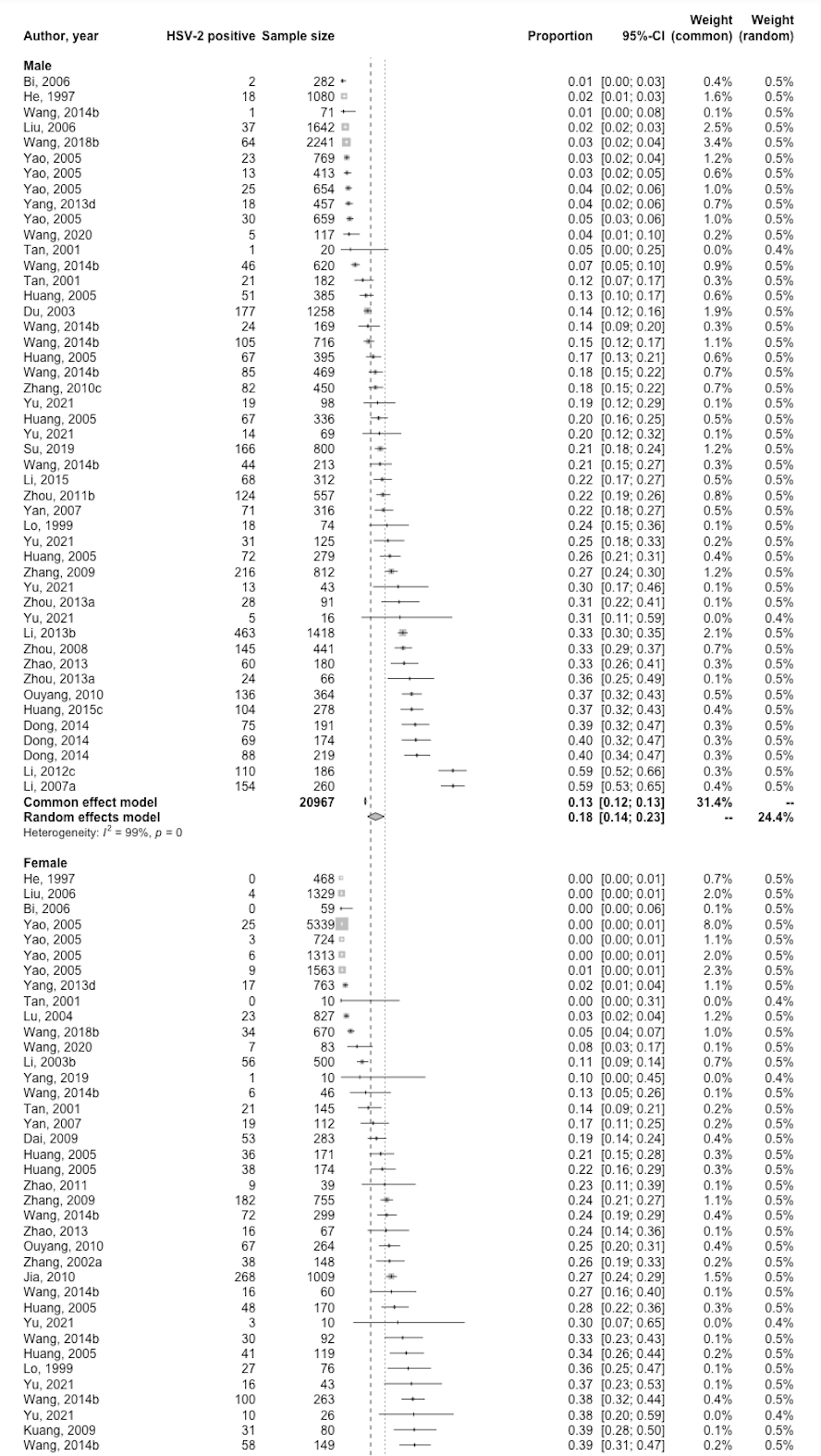


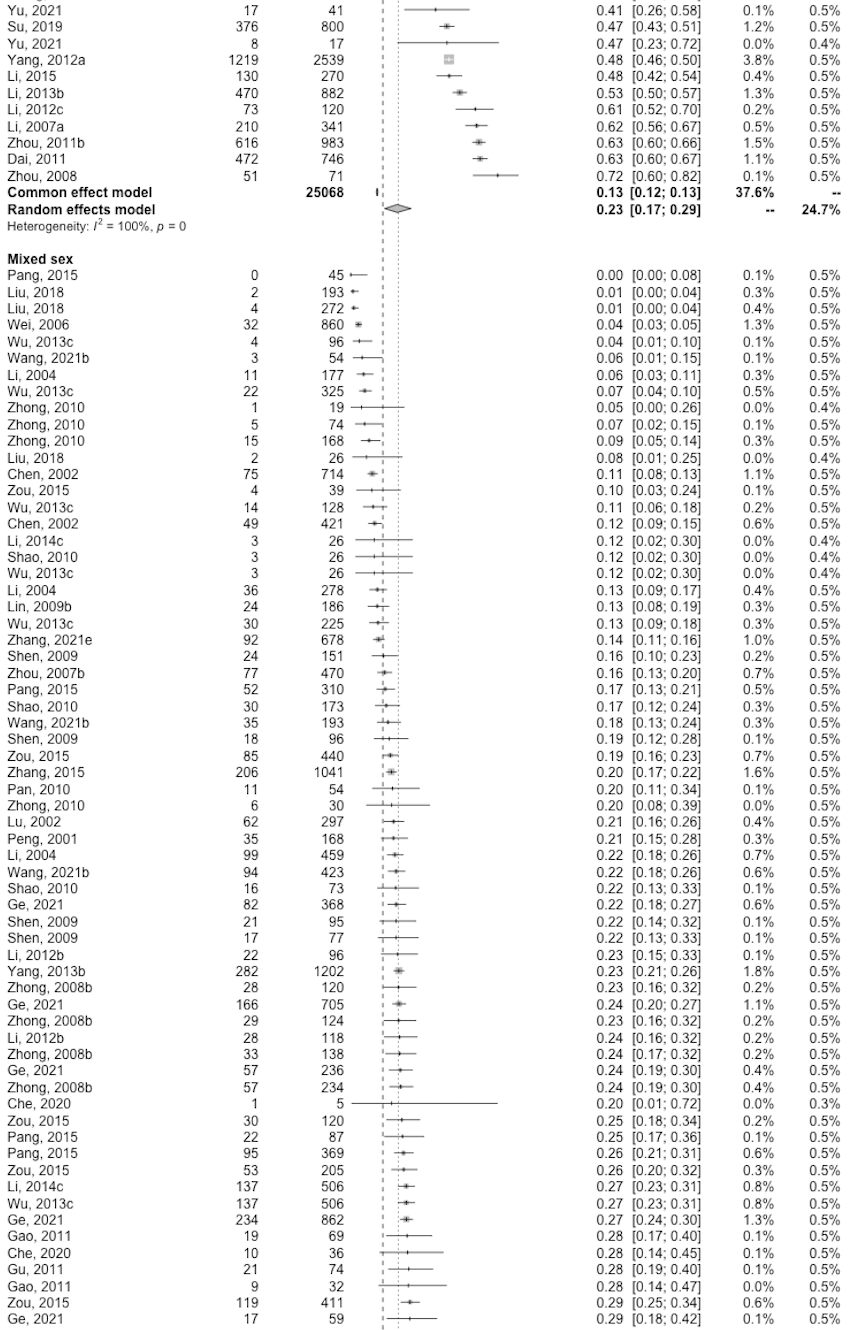


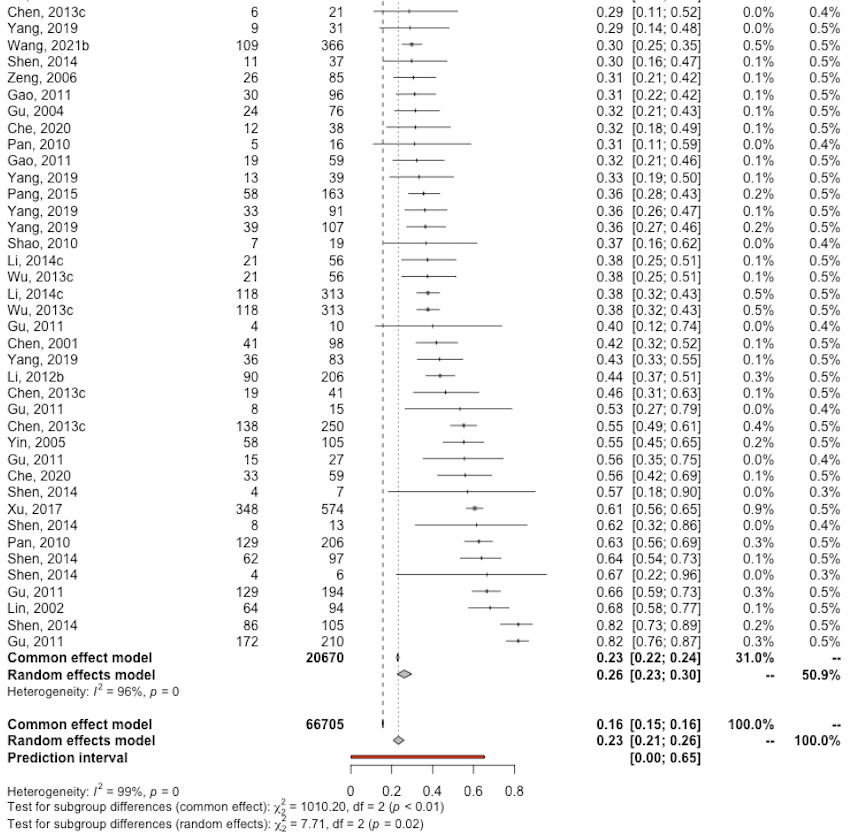


## HIV-positive individuals and individuals in HIV-discordant couples


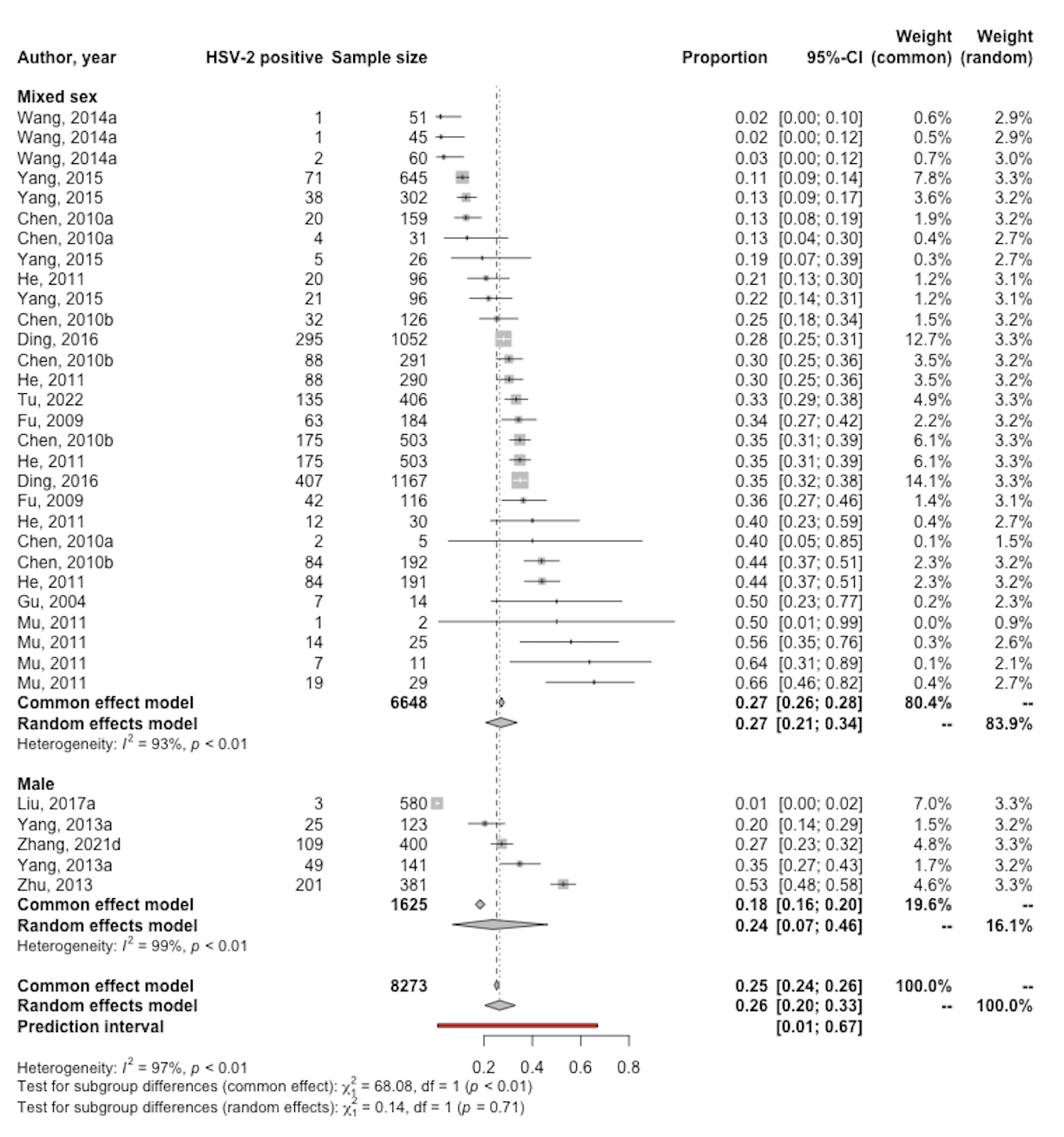


## Other populations


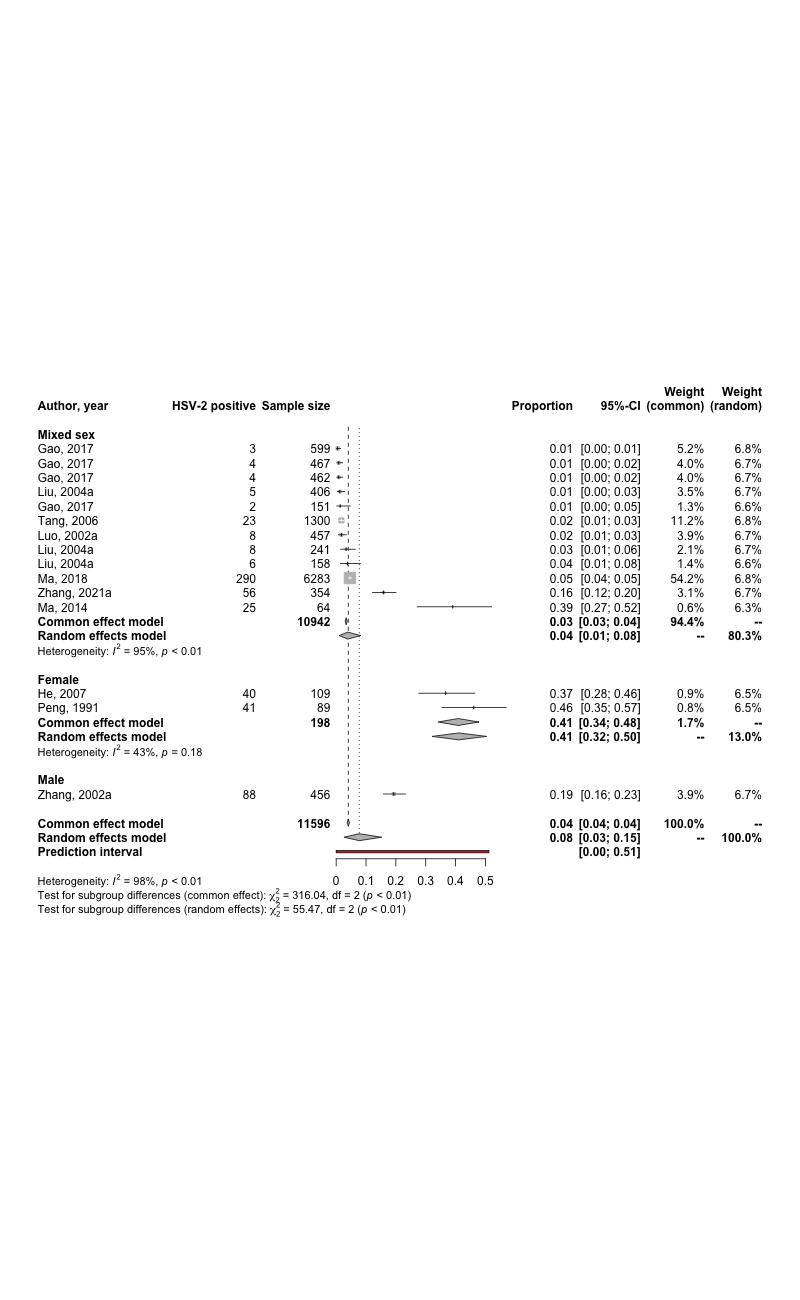


# **Supplementary Table S10.** Summary of precision assessment and risk of bias (ROB) assessment for studies reporting HSV-2 seroprevalence in China.

| **Quality assessment** | **HSV-2 seroprevalence measures** | |  |  |
| --- | --- | --- | --- | --- |
|  | **Total**  **N (%)** | **English**  **N (%)** | **Chinese**  **N (%)** | **p-value ^c^** |
| **Precision of seroprevalence measures^a^** | | |  |  |
| Low precision (sample size < 200) | 61 (15.2) | 6 (10.0) | 55 (16.1) | 0.010 |
| High precision (sample size ≥ 200) | 341 (84.8) | 54 (90.0) | 287 (83.9) |  |
| **Risk of bias quality domain** | | |  |  |
| **Sampling method** | | |  | <0.001 |
| Low risk of bias (probability-based sampling) | 27 (6.7) | 11 (18.3) | 16 (4.7) |  |
| High risk of bias (non-probability-based sampling) | 375 (93.3) | 49 (81.7) | 326 (95.3) |  |
| **Response rate** | | |  | 0.001 |
| Low risk of bias (≥ 80%) | 84 (20.9) | 16 (26.7) | 68 (19.9) |  |
| High risk of bias (< 80%) | 9 (2.2) | 5 (8.3) | 4 (1.2) |  |
| Unclear risk of bias (unclear) **^b^** | 309 (76.9) | 39 (65.0) | 270 (78.9) |  |
| **Summary of the risk of bias assessment** | | |  |  |
| **Low risk of bias** |  |  |  |  |
| In at least one quality domain | 99 (24.6) | 22 (36.7) | 77 (22.5) | 0.029 |
| In both quality domains | 12 (3.0) | 5 (8.3) | 7 (2.1) | 0.021 |
| **High risk of bias** |  |  |  |  |
| In at least one quality domain | 376 (93.5) | 49 (81.7) | 327 (95.6) | <0.001 |
| In both quality domains | 9 (2.2) | 5 (8.3) | 4 (1.2) | 0.001 |
| **Total count of seroprevalence studies** | **402 (100)** | **60 (100)** | **342 (100)** |  |

^a^ Precision was assessed based on the overall sample size (not each stratum subsample size) of the study as reported in the record/publication.

^b^ The response rate was unclear if a study used preexisting medical records.

^c^ P-value was calculated using Chi-square tests or Fisher’s exact tests.

Abbreviations: HSV-2 = Herpes simplex virus type 2.
